# Supplementary material for: Another Angle on Benchmarking Noncovalent Interactions
Source: J Chem Theory Comput. 2025 Feb 26;21(5):2311–24. doi: 10.1021/acs.jctc.4c01512 (PMC11912214; doi:10.1021/acs.jctc.4c01512)
Supplement: Supplementary file 1 — ct4c01512_si_001.pdf [file ct4c01512_si_001.pdf]

Supporting Information: Another Angle on Benchmarking Noncovalent Interactions

Vladimir Fishman,† Michał Lesiuk,‡ Jan M. L. Martin,*,† and A. Daniel Boese*,¶

†*Department of Molecular Chemistry and Materials Science, Weizmann Institute of Science, 7610001 Reḥovot, Israel*

‡*Quantum Chemistry Laboratory, Faculty of Chemistry, University of Warsaw, L. Pasteura 1 St., 02-093 Warsaw, Poland*

¶*Department of Chemistry, University of Graz, Heinrichstrasse 28/IV, 8010 Graz, Austria*

E-mail: gershom@weizmann.ac.il; adrian_daniel.boese@uni-graz.at

Contents

L7 Set and Discrepancies between two Reference Methods **S-3**

Raw CCSD(T) Correlation Data: Basis Set Effects and Local Correlation **S-6**

Raw Hartree-Fock, RPA and DFT-SAPT Data: Basis Set Effects **S-47**

Electronic Structure Methods **S-69**

Rank-reduced Calculations for the Benzene and Naphtalene Dimers **S-81**

Best Estimates of Slopes **S-82**

Assessment of the Results when extrapolating to larger Molecules **S-83**

Strong Correlation Diagnostic of the Acene Species **S-91**

L7 Set and Discrepancies between two Reference Methods

Here, we report our DFT-SAPT values with different basis sets using both PBE0-AC as well as PBE-AC in detail. The best DFT-SAPT values using the largest basis sets are each in the last row and the second column with the DFT-SAPT (using the PBE0-AC functional).

Table S1: Interaction energies of different DFT-SAPT values for the benzene dimer in millihartree using PBE0-AC as underlying density functional. The respective values from the electrostatic and induction energies are taken from the larger basis sets for the extrapolated basis sets.

Basis Set	all	elec+ind	dispersion
DZ	0.024	7.861	-7.837
aDZ	-3.450	7.613	-10.063
TZ	-2.841	7.769	-10.610
DTZ	-9.427	7.769	-17.197
aTZ	-4.253	7.690	-11.942
aDTZ	-6.342	7.690	-14.032
QZ	-3.899	7.747	-11.646
TQZ	-5.318	7.747	-13.066
aQZ	-4.525	7.688	-12.213
aTQZ	-4.896	7.688	-12.584

Table S2: Interaction energies of different DFT-SAPT values for the guanine-cytosine (tetramer) dimer of the L7 Set in millihartree. The respective values from the electrostatic and induction energies are taken from the larger basis sets for the extrapolated basis sets.

	PBE0-AC			PBE-AC		
Basis Set	all	elec+ind	dispersion	all	elec+ind	dispersion
DZ	-14.323	22.093	-36.416	-12.128	25.108	-37.236
aDZ	-17.047	21.931	-38.978	-15.008	24.884	-39.892
TZ	-18.484	21.915	-40.400	-16.403	25.009	-41.412
DTZ	-20.161	21.915	-42.077	-18.161	25.009	-43.170
aTZ	-19.537	21.855	-41.391	-17.499	24.938	-42.437
aDTZ	-20.553	21.855	-42.077	-18.571	24.938	-43.509
QZ	-19.791	21.856	-41.647	-17.758	24.952	-42.710
TQZ	-20.701	21.856	-42.558	-18.706	24.952	-43.658
aQZ	-20.277	21.828	-42.105	-18.271	24.906	-43.177
aTQZ	-20.798	21.828	-42.626	-18.810	24.906	-43.716
5Z	-20.389	21.798	-42.189	-18.387	24.871	-43.257
Q5Z	-20.958	21.798	-42.756	-18.961	24.871	-43.831
a5Z	-20.563	21.787	-42.350	-18.558	24.867	-43.424
aQ5Z	-20.820	21.787	-42.607	-18.818	24.867	-43.685

Table S3: Interaction energies of different DFT-SAPT values for the coronene dimer of the L7 Set in millihartree. The respective values from the electrostatic and induction energies are taken from the larger basis sets for the extrapolated basis sets.

	PBE0-AC			PBE-AC		
Basis Set	all	elec+ind	dispersion	all	elec+ind	dispersion
DZ	-14.189	25.793	-39.982	-11.712	28.305	-40.018
aDZ				-24.605	27.996	-52.601
TZ	-24.900	25.961	-50.861	-22.630	28.452	-51.082
DTZ	-29.481	25.961	-55.442	-27.288	28.452	-55.740
aTZ	-29.056	25.775	-54.830	-27.364	28.189	-55.553
aDTZ				-18.571	28.189	-56.796
QZ	-24.284	25.858	-54.142	-26.144	28.299	-54.443
TQZ	-30.678	25.858	-56.537	-28.596	28.299	-56.895
aQZ				-27.968	28.125	-56.093
aTQZ				-28.361	28.125	-56.486
5Z	-29.718	25.677	-55.395	-27.678	28.005	-55.683
Q5Z	-31.032	25.677	-56.709	-28.979	28.005	-56.984

Table S4: Interaction energies of different DFT-SAPT values for the circumcoronene-adenine dimer of the L7 Set in millihartree. The respective values from the electrostatic and induction energies are taken from the larger basis sets for the extrapolated basis sets.

	PBE0-AC			PBE-AC		
Basis Set	all	elec+ind	dispersion	all	elec+ind	dispersion
DZ	-16.305	16.279	-32.584	-14.076	18.858	-32.934
TZ	-21.108	16.483	-32.591	-19.106	19.019	-38.124
DTZ	-23.217	16.483	-39.699	-21.291	19.019	-40.310
QZ	-22.658	16.454	-39.112	-20.715	18.979	-39.695
TQZ	-23.768	16.454	-40.222	-21.861	18.979	-40.840

Table S5: Interaction energies of different DFT-SAPT values for the circumcoronene-gc dimer of the L7 Set in millihartree. The respective values from the electrostatic and induction energies are taken from the larger basis sets for the extrapolated basis sets. The best estimate value in parentheses includes the (D,T)Z basis set incompleteness estimation from the PBE-AC functional.

	PBE0-AC			PBE-AC		
Basis Set	all	elec+ind	dispersion	all	elec+ind	dispersion
DZ	-25.919	32.671	-58.590	-22.415	36.767	-59.182
TZ	-35.010	32.341	-67.351	-31.728	36.502	-68.230
DTZ	-38.699	32.341	-71.039	-35.538	36.502	-72.040
QZ				-34.600	36.470	-71.070
TQZ	(-39.8)			-36.673	36.470	-73.143

Table S6: Interaction energies of different DFT-SAPT values for the buckycatcher dimer in millihartree. The respective values from the electrostatic and induction energies are taken from the larger basis sets for the extrapolated basis sets. The best estimate value in parentheses includes the (D,T)Z basis set incompleteness estimation from the PBE-AC functional.

	PBE0-AC			PBE-AC		
Basis Set	all	elec+ind	dispersion	all	elec+ind	dispersion
DZ	-28-108	89.747	-117.85	-20.949	96.496	-117.45
TZ				-41.109	98.162	-139.27
DTZ	(-60.1)			-50.298	98.162	-148.46

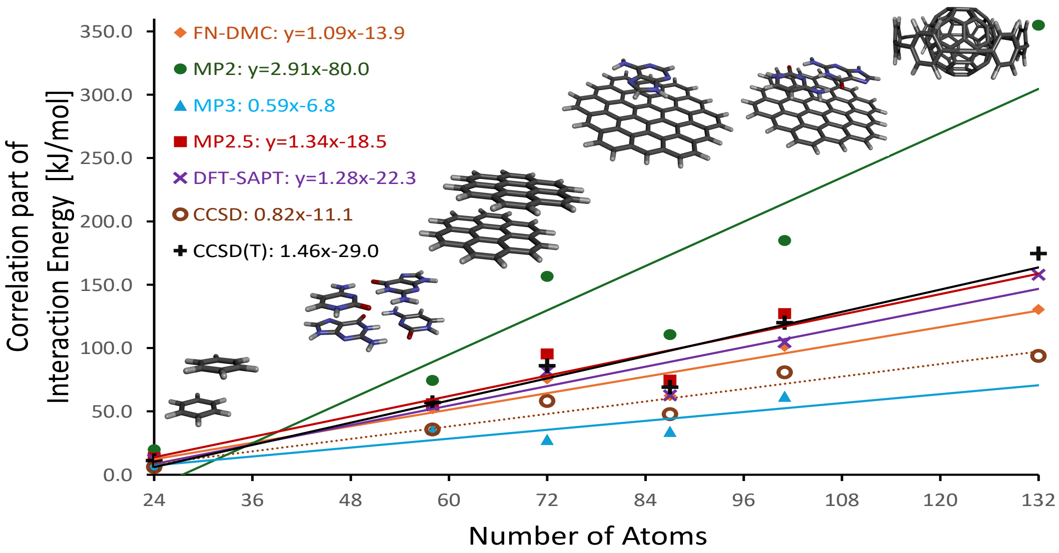

Figure S1: Correlation energies in kJ/mol vs. number of atoms.

Raw CCSD(T) Correlation Data: Basis Set Effects and Local Correlation

In this section, all CCSD, CCSD(T), as well as LNO-CCSD, LNO-CCSD(T), DLPNO-CCSD, and DLPNO-CCSD(T) raw data for various basis sets (cc-pVXZ with X=(D,T,Q,5) and aug-cc-pVYZ with Y=(T,Q,5)) is displayed.

Table S7: CCSD(T) correlation part of the interaction energies for different acene dimers for the cc-pVDZ basis set in millihartree (cp-corrected).

DZ		CCSD(T) cor	LNO-CCSD(T) cor				DLPNO-CCSD(T) Tight	
System	Atoms	canonical	vvTight	vTight	Tight	Normal	TcutPNO=1e-6	TcutPNO=1e-7
c_c	12	-5.821	-5.952	-6.005	-6.559	-7.155	-5.698	-5.917
c2_c2	36	-12.169	-12.483	-12.573	-13.594	-15.180	-12.256	-12.443
c3_c1	36	-11.711	-11.884	-11.982	-12.588	-14.720	-11.622	-11.929
c3_c3	48	-18.607	-19.053	-19.229	-20.951	-23.791	-18.885	-19.327
c4_c2	48	-18.049	-18.490	-18.661	-19.779	-21.745	-18.125	-18.582
cc_c	48	-16.712						
c5_c1	48	-12.593						
c4_c4	60	-25.049	-25.754	-25.962	-27.966	-31.397	-25.748	-26.283
c5_c3	60	-25.075	-25.729	-25.996	-27.524	-30.661	-25.384	-26.121
c6_c2	60	-19.403						
c7_c1	60	-12.829						
c5_c5	72	-31.620	-32.541	-32.956	-35.290	-39.899	-32.620	-33.457
c6_c4	72	-31.287	-32.150	-32.494	-34.691	-38.914	-31.980	-32.821
c6_c6	84		-39.302	-39.870	-42.865	-48.215	-39.685	-40.574
c7_c5	84		-40.673	-41.153	-43.696	-48.936		

Table S8: CCSD correlation part of the interaction energies for different acene dimers for the cc-pVDZ basis set in millihartree (cp-corrected).

DZ		CCSD cor	LNO-CCSD cor				DLPNO-CCSD Tight	
System	Atoms	canonical	vvTight	vTight	Tight	Normal	TcutPNO=1e-6	TcutPNO=1e-7
c_c	12	-4.954	-5.090	-5.099	-5.441	-5.207	-5.004	-5.113
c2_c2	36	-10.223	-10.570	-10.574	-11.189	-11.543	-10.782	-10.738
c3_c1	36	-9.826	-10.064	-10.097	-10.299	-10.723	-10.222	-10.283
c3_c3	48	-15.530	-16.029	-16.059	-16.912	-17.384	-16.686	-16.707
c4_c2	48	-15.036	-15.534	-15.565	-15.921	-17.797	-15.999	-16.039
cc_c	48	-13.978					-14.899	-14.949
c5_c1	48	-10.526					-11.385	-11.511
c4_c4	60	-20.817	-21.532	-21.534	-22.360	-22.736	-22.797	-22.780
c5_c3	60	-20.810	-21.505	-21.574	-22.062	-23.510	-22.462	-22.581
c6_c2	60	-16.116					-17.634	-17.733
c7_c1	60	-10.715					-11.709	-11.856
c5_c5	72	-26.201	-27.104	-27.180	-27.943	-28.739	-28.939	-29.012
c6_c4	72	-25.900	-26.772	-26.835	-27.621	-28.209	-28.337	-28.420
c7_c3	72						-24.022	-24.221
c8_c2	72							
c9_c1	72							
c6_c6	84		-32.682	-32.791	-33.817	-35.270	-35.177	-35.211
c7_c5	84		-33.830	-33.922	-34.687	-37.036		

Table S9: CCSD(T) correlation part of the interaction energies for different acene dimers for the aug-cc-pVDZ basis set in millihartree (cp-corrected).

aDZ		CCSD(T) cor	LNO-CCSD(T) cor				DLPNO-CCSD(T) Tight	
System	Atoms	canonical	vvTight	vTight	Tight	Normal	TcutPNO=1e-6	TcutPNO=1e-7
c_c	12	-8.056	-8.152	-8.186	-8.323	-8.939	-7.835	-8.004
c2_c2	36	-16.552	-16.723	-16.804	-17.303	-18.611	-16.161	-16.520
c3_c1	36						-15.739	-16.168
c3_c3	48	-25.126	-25.534	-25.699	-26.607	-28.542		
c4_c2	48						-24.334	-25.091
c4_c4	60		-34.368	-34.576	-35.645	-38.730	-33.908	
c5_c5	72			-43.480	-44.902	-48.877		
c6_c6	84			-52.278	-54.181	-59.058		

Table S10: CCSD correlation part of the interaction energies for different acene dimers for the aug-cc-pVDZ basis set in millihartree (cp-corrected).

aDZ		CCSD cor	LNO-CCSD cor				DLPNO-CCSD Tight	
System	Atoms	canonical	vvTight	vTight	Tight	Normal	TcutPNO=1e-6	TcutPNO=1e-7
c_c	12	-6.688	-6.852	-6.895	-6.819	-6.731	-6.761	-6.785
c2_c2	36	-13.584	-13.887	-13.959	-14.049	-13.852	-13.938	-13.924
c3_c1	36						-13.597	-13.620
c3_c3	48	-20.495	-21.075	-21.173	-21.416	-21.079		
c4_c2	48						-21.088	-21.187
c4_c4	60		-28.208	-28.281	-28.517	-28.467	-29.364	
c5_c5	72			-35.430	-35.790	-35.733		
c6_c6	84			-42.477	-42.951	-43.174		

Table S11: CCSD(T) correlation part of the interaction energies for different acene dimers for the cc-pVTZ basis set in millihartree (cp-corrected).

TZ		CCSD(T) cor	LNO-CCSD(T) cor				DLPNO-CCSD(T) Tight	
System	Atoms	canonical	vvTight	vTight	Tight	Normal	TcutPNO=1e-6	TcutPNO=1e-7
c_c	12	-7.256	-7.344	-7.358	-7.855	-9.164	-7.197	-7.296
c2_c2	36	-15.122	-15.435	-15.462	-16.778	-18.898	-15.014	-15.266
c3_c1	36	-14.899	-15.167	-15.219	-15.851	-17.512	-14.634	-15.015
c3_c3	48	-23.114	-23.698	-23.771	-25.500	-28.045	-23.180	-23.706
c4_c2	48	-22.785	-23.300	-23.392	-24.509	-27.434	-22.618	-23.279
c5_c1	48	-16.098						
c4_c4	60	-31.097	-32.000	-32.138	-33.706	-37.840	-31.375	-32.079
c5_c3	60		-32.349	-32.502	-34.248	-38.026	-31.517	-32.369
c5_c5	72			-40.537	-42.850	-48.244	-39.680	
c6_c4	72			-40.489	-42.636	-47.514	-39.516	
c6_c6	84			-48.986	-51.597	-58.205		
c7_c5	84			-51.117	-53.791	-59.943		

Table S12: CCSD correlation part of the interaction energies for different acene dimers for the cc-pVTZ basis set in millihartree (cp-corrected).

TZ		CCSD cor	LNO-CCSD cor				DLPNO-CCSD Tight	
System	Atoms	canonical	vvTight	vTight	Tight	Normal	TcutPNO=1e-6	TcutPNO=1e-7
c_c	12	-5.984	-6.149	-6.167	-6.372	-6.719	-6.202	-6.163
c2_c2	36	-12.343	-12.787	-12.796	-13.480	-14.033	-12.956	-12.860
c3_c1	36	-12.188	-12.592	-12.625	-12.879	-13.152	-12.671	-12.677
c3_c3	48	-18.764	-19.524	-19.532	-20.340	-20.547	-20.054	-20.003
c4_c2	48	-18.511	-19.205	-19.241	-19.694	-20.327	-19.612	-19.663
cc_c	48						-18.622	-18.566
c5_c1	48						-14.066	-14.091
c4_c4	60	-25.155	-26.249	-26.292	-26.795	-27.480	-27.218	-27.126
c5_c3	60		-26.550	-26.608	-27.342	-27.874	-27.388	-27.382
c7_c1	60						-14.459	-14.504
c5_c5	72			-33.043	-33.858	-34.894	-34.488	
c6_c4	72			-33.026	-33.877	-34.698	-34.419	
c6_c6	84			-39.780	-40.747	-42.243	69.296	
c7_c5	84			-41.648	-42.667	-43.857		

Table S13: CCSD(T) correlation part of the interaction energies for different acene dimers for the aug-cc-pVTZ basis set in millihartree (cp-corrected).

aTZ		CCSD(T) cor	LNO-CCSD(T) cor				DLPNO-CCSD(T) Tight	
System	Atoms	canonical	vvTight	vTight	Tight	Normal	TcutPNO=1e-6	TcutPNO=1e-7
c_c	12	-8.180	-8.265	-8.288	-8.488	-9.037	-7.990	-8.133
c2_c2	36	-16.816	-17.111	-17.156	-17.613	-18.785	-16.592	-16.880
c3_c1	36	-16.624591	-16.815	-16.873	-17.171	-18.251	-16.313	-16.669
c3_c3	48		-26.170	-26.173	-26.971	-29.102	-25.548	
c4_c2	48			-25.772	-26.396	-28.170	-25.146	-25.748
c4_c4	60			-35.214	-36.246	-39.353	-34.729	
c5_c3	60			-35.687	-36.589	-39.324	-34.818	
c5_c5	72			-44.300	-45.820	-49.745		
c6_c4	72				-45.523	-49.184	-43.588	
c6_c6	84			-48.986	-55.743	-60.272		
c7_c5	84				-57.470	-62.219		

Table S14: CCSD correlation part of the interaction energies for different acene dimers for the aug-cc-pVTZ basis set in millihartree (cp-corrected).

aTZ		CCSD cor	LNO-CCSD cor				DLPNO-CCSD Tight	
System	Atoms	canonical	vvTight	vTight	Tight	Normal	TcutPNO=1e-6	TcutPNO=1e-7
c_c	12	-6.668	-6.847	-6.862	-6.930	-6.695	-6.854	-6.805
c2_c2	36	-13.594	-14.041	-14.078	-14.127	-13.838	-14.223	-14.100
c3_c1	36	-13.472	-13.828	-13.867	-13.872	-13.629	-14.015	-13.945
c3_c3	48		-21.362	-21.312	-21.467	-21.277	-21.971	
c4_c2	48			-20.999	-21.072	-20.711	-21.658	-21.589
c4_c4	60			-28.507	-28.707	-28.540	-29.932	
c5_c3	60			-28.934	-29.073	-28.825	-30.071	
c5_c5	72			-35.788	-36.144	-36.069		
c6_c4	72				-35.989	-35.766	-37.661	
c6_c6	84				-43.866	-43.429		
c7_c5	84				-45.361	-45.338		

Table S15: CCSD(T) correlation part of the interaction energies for different acene dimers for the cc-pVQZ basis set in millihartree (cp-corrected).

QZ		CCSD(T)	LNO-CCSD(T)				DLPNO-CCSD(T) Tight	
System	Atoms	canonical	vvTight	vTight	Tight	Normal	TcutPNO=1e-6	TcutPNO=1e-7
c_c	12	-7.827	-7.917	-7.970	-8.411	-9.311	-7.744	-7.839
c2_c2	36	-16.240	-16.504	-16.587	-17.357	-18.996	-16.097	-16.343
c3_c1	36		-16.308	-16.397	-16.778	-18.247		
c3_c3	48			-25.423	-26.544	-28.825		
c4_c2	48		-25.455	-25.081	-25.819	-28.229		
c4_c4	60			-34.265	-35.721	-39.043		
c5_c3	60			-34.767	-35.918	-39.136		
c5_c5	72			-43.195	-45.023	-49.271		
c6_c4	72				-44.776	-48.982		
c6_c6	84				-54.188	-59.574		
c7_c5	84				-56.569	-61.730		

Table S16: CCSD correlation part of the interaction energies for different acene dimers for the cc-pVQZ basis set in millihartree (cp-corrected).

QZ		CCSD cor	LNO-CCSD cor				DLPNO-CCSD Tight	
System	Atoms	canonical	vvTight	vTight	Tight	Normal	TcutPNO=1e-6	TcutPNO=1e-7
c_c	12	-6.389	-6.573	-6.616	-6.820	-6.797	-6.633	-6.573
c2_c2	36	-13.136	-13.576	-13.630	-13.850	-13.868	-13.817	-13.676
c3_c1	36		-13.454	-13.511	-13.556	-13.500		
c3_c3	48			-20.736	-21.001	-20.955		
c4_c2	48		-20.411	-20.488	-20.597	-20.627		
c4_c4	60			-27.815	-28.180	-28.194		
c5_c3	60			-28.276	-28.470	-28.575		
c5_c5	72			-34.937	-35.410	-35.520		
c6_c4	72				-35.340	-35.522		
c6_c6	84				-42.498	-42.897		
c7_c5	84				-44.588	-44.859		

Table S17: CCSD(T) correlation part of the interaction energies for different acene dimers for the aug-cc-pVQZ basis set in millihartree (cp-corrected).

aQZ		CCSD(T)	LNO-CCSD(T)			
System	Atoms	canonical	vvTight	vTight	Tight	Normal
c_c	12	-8.236	-8.312	-8.345	-8.488	-9.106
c2_c2	36			-17.248	-17.701	-19.095
c3_c1	36			-16.990	-17.361	-18.550
c3_c3	48			-26.297	-27.116	-29.207
c4_c2	48			-25.934	-26.602	-28.557
c4_c4	60			-35.356	-36.259	-39.598
c5_c3	60				-37.078	-39.993
c5_c5	72				-45.859	-49.840
c6_c4	72				-45.769	-49.706
c6_c6	84				-55.340	-60.366
c7_c5	84				-57.764	-62.548

Table S18: CCSD correlation part of the interaction energies for different acene dimers for the aug-cc-pVQZ basis set in millihartree (cp-corrected).

aQZ		CCSD cor	LNO-CCSD cor			
System	Atoms	canonical	vvTight	vTight	Tight	Normal
c_c	12	-6.690	-6.869	-6.898	-6.869	-6.681
c2_c2	36			-14.115	-14.141	-13.960
c3_c1	36			-13.951	-13.982	-13.842
c3_c3	48			-21.373	-21.482	-21.316
c4_c2	48			-21.102	-21.126	-20.995
c4_c4	60			-28.585	-28.593	-28.620
c5_c3	60				-29.345	-29.233
c5_c5	72				-36.021	-35.939
c6_c4	72				-35.997	-36.122
c6_c6	84				-43.315	-43.455
c7_c5	84				-45.376	-45.465

Table S19: CCSD(T) correlation part of the interaction energies for different acene dimers for the cc-pV5Z basis set in millihartree (cp-corrected).

5Z		CCSD(T) cor	LNO-CCSD(T) cor			
System	Atoms	canonical	vvTight	vTight	Tight	Normal
c_c	12	-8.118	-8.294	-8.321	-8.625	-9.289
c2_c2	36			-17.075	-17.769	-19.398
c3_c1	36			-16.856	-17.320	-18.602
c3_c3	48			-26.105	-27.072	-29.599
c4_c2	48			-25.772	-26.595	-28.717
c4_c4	60			-35.088	-36.252	-39.725
c5_c3	60				-36.807	-39.830
c5_c5	72				-45.838	-49.853
c6_c4	72				-46.152	-50.108
c6_c6	84				-55.328	-60.690
c7_c5	84				-57.757	-62.575

Table S20: CCSD correlation part of the interaction energies for different acene dimers for the cc-pV5Z basis set in millihartree (cp-corrected).

5Z		CCSD cor	LNO-CCSD cor			
System	Atoms	canonical	vvTight	vTight	Tight	Normal
c_c	12	-6.598	-6.851	-6.853	-6.958	-6.767
c2_c2	36			-13.968	-14.109	-14.109
c3_c1	36			-13.829	-13.880	-13.754
c3_c3	48			-21.194	-21.371	-21.450
c4_c2	48			-20.962	-21.075	-20.958
c4_c4	60			-28.383	-28.511	-28.710
c5_c3	60				-29.041	-29.013
c5_c5	72				-35.904	-35.932
c6_c4	72				-36.210	-36.302
c6_c6	84				-43.145	-43.678
c7_c5	84				-45.280	-45.463

Table S21: CCSD(T) correlation part of the interaction energies for different acene dimers for the aug-cc-pV5Z basis set in millihartree (cp-corrected).

a5Z		CCSD(T) cor	LNO-CCSD(T) cor			
System	Atoms	canonical	vvTight	vTight	Tight	Normal
c_c	12		-8.345	-8.412	-8.640	-9.178
c2_c2	36			-17.281	-17.739	-19.117
c3_c1	36			-17.058	-17.405	-18.628
c3_c3	48			-26.333	-27.080	-29.387
c4_c2	48				-26.695	-28.764
c4_c4	60				-36.370	-39.693
c5_c3	60				-36.789	-39.815
c5_c5	72				-46.135	-50.140
c6_c4	72				-45.917	-49.954
c6_c6	84				-55.443	-60.520
c7_c5	84				-57.937	-62.943

Table S22: CCSD correlation part of the interaction energies for different acene dimers for the aug-cc-pV5Z basis set in millihartree (cp-corrected).

a5Z		CCSD cor	LNO-CCSD cor			
System	Atoms	canonical	vvTight	vTight	Tight	Normal
c_c	12		-6.893	-6.939	-6.993	-6.790
c2_c2	36			-14.115	-14.194	-14.036
c3_c1	36			-13.981	-14.013	-13.891
c3_c3	48			-21.368	-21.480	-21.435
c4_c2	48				-21.255	-21.155
c4_c4	60				-28.717	-28.696
c5_c3	60				-29.159	-29.080
c5_c5	72				-36.236	-36.152
c6_c4	72				-36.151	-36.312
c6_c6	84				-43.410	-43.651
c7_c5	84				-45.536	-45.676

Table S23: CCSD(T) correlation part of the interaction energies for different acene dimers for the cc-pVDZ basis set in millihartree (not cp-corrected).

DZ		CCSD(T) cor	LNO-CCSD(T) cor				DLPNO-CCSD(T) Tight	
System	Atoms	canonical	vvTight	vTight	Tight	Normal	TcutPNO=1e-6	TcutPNO=1e-7
c_c	12	-6.344	-6.509	-6.561	-6.829	-7.394	-6.183	-6.333
c2_c2	36	-13.701	-14.125	-14.224	-14.810	-15.999	-13.542	-13.766
c3_c1	36	-13.778	-14.026	-14.116	-14.691	-16.042	-13.682	-13.969
c3_c3	48	-21.155	-21.745	-21.916	-23.100	-24.936	-21.099	-21.504
c4_c2	48	-21.114	-21.651	-21.823	-22.791	-24.696	-20.944	-21.400
cc_c	48	-20.459	-20.785	-20.940	-21.646	-23.740		
c5_c1	48	-14.881	-15.171	-15.305	-15.901	-17.369		
c4_c4	60	-28.598	-29.482	-29.707	-31.443	-34.086	-28.522	-29.041
c5_c3	60	-29.323	-30.144	-30.390	-31.785	-34.437	-29.266	-29.945
c6_c2	60	-22.830	-23.441	-23.687	-24.627	-26.743		
c7_c1	60	-15.154	-15.459	-15.615	-16.193	-17.739		
cc_cc	72		-45.395	-46.002	-48.283	-53.433		
c5_c5	72	-36.185	-37.295	-37.684	-39.848	-43.279	-36.071	-36.880
c6_c4	72	-36.441	-37.468	-37.802	-39.734	-43.396	-36.193	-37.058
c7_c3	72		-31.857	-32.186	-33.618	-36.572		
c8_c2	72		-23.836	-24.100	-25.069	-27.120		
c9_c1	72		-15.595	-15.712	-16.418	-17.900		
c6_c6	84		-45.057	-45.582	-48.119	-52.360	-43.784	-44.534
c7_c5	84		-47.506	-47.964	-50.278	-54.906		
c8_c4	84		-40.507	-40.882	-42.895	-46.688		
ccc_c	84		-23.272	-23.676	-24.742	-27.328		
c10_c4	96					-68.222		
ccc_cc	108				-82.038	-90.821		
ccc_ccc	144				-127.135	-142.896		

Table S24: CCSD correlation part of the interaction energies for different acene dimers for the cc-pVDZ basis set in millihartree (not cp-corrected).

DZ		CCSD cor	LNO-CCSD cor				DLPNO-CCSD Tight	
System	Atoms	canonical	vvTight	vTight	Tight	Normal	TcutPNO=1e-6	TcutPNO=1e-7
c_c	12	-5.338	-5.493	-5.503	-5.507	-5.251	-5.402	-5.430
c2_c2	36	-11.405	-11.831	-11.848	-11.808	-11.580	-11.857	-11.794
c3_c1	36	-11.449	-11.738	-11.758	-11.842	-11.454	-11.949	-11.943
c3_c3	48	-17.516	-18.126	-18.127	-18.361	-17.871	-18.545	-18.483
c4_c2	48	-17.448	-18.026	-18.045	-18.177	-17.778	-18.369	-18.334
cc_c	48						-17.947	-17.950
c5_c1	48	-12.331					-13.226	
c4_c4	60	-23.596	-24.464	-24.465	-24.908	-24.420	-25.141	-25.036
c5_c3	60	-24.161	-24.988	-25.017	-25.276	-24.676	-25.741	-25.740
c6_c2	60	-18.825					-20.354	-20.351
c7_c1	60	-12.549					-13.608	-13.644
cc_cc	72							
c5_c5	72	-29.786	-30.846	-30.880	-31.300	-30.743	-31.877	-31.845
c6_c4	72	-29.968	-30.966	-31.006	-31.408	-31.031	-31.925	-31.921
c7_c3	72						-27.698	
c6_c6	84		-37.212	-37.274	-37.756	-37.377	-38.720	-38.537
c7_c5	84		-39.238	-39.290	-39.691	-39.339		

Table S25: CCSD(T) correlation part of the interaction energies for different acene dimers for the aug-cc-pVDZ basis set in millihartree (not cp-corrected).

aDZ		CCSD(T) cor	LNO-CCSD(T) cor				DLPNO-CCSD(T) Tight	
System	Atoms	canonical	vvTight	vTight	Tight	Normal	TcutPNO=1e-6	TcutPNO=1e-7
c_c	12	-10.428	-10.566	-10.597	-10.839	-11.472	-10.134	-10.308
c2_c2	36	-22.160	-22.326	-22.400	-22.982	-24.371	-21.503	-21.865
c3_c1	36						-21.901	-22.378
c3_c3	48	-34.096	-34.532	-34.674	-35.604	-37.677		
c4_c2	48						-33.526	-34.285
c4_c4	60		-46.985	-47.232	-48.198	-51.311	-45.239	
c5_c5	72			-59.985	-61.298	-65.132		
c6_c6	84			-72.297	-73.909	-78.925		

Table S26: CCSD correlation part of the interaction energies for different acene dimers for the aug-cc-pVDZ basis set in millihartree (not cp-corrected).

aDZ		CCSD cor	LNO-CCSD cor				DLPNO-CCSD Tight	
System	Atoms	canonical	vvTight	vTight	Tight	Normal	TcutPNO=1e-6	TcutPNO=1e-7
c_c	12	-8.720	-8.930	-8.961	-9.013	-8.894	-8.791	-8.784
c2_c2	36	-18.371	-18.688	-18.742	-18.901	-18.719	-18.595	-18.552
c3_c1	36						-18.942	-18.950
c3_c3	48	-28.132		-28.815	-29.036	-28.845		
c4_c2	48						-29.076	-29.108
c4_c4	60		-38.952	-39.058	-39.141	-39.068	-39.339	
c5_c5	72			-49.486	-49.673	-49.451		
c6_c6	84			-59.495	-59.672	-59.901		

Table S27: CCSD(T) correlation part of the interaction energies for different acene dimers for the cc-pVTZ basis set in millihartree (not cp-corrected).

TZ		CCSD(T) cor	LNO-CCSD(T) cor				DLPNO-CCSD(T) Tight	
System	Atoms	canonical	vvTight	vTight	Tight	Normal	TcutPNO=1e-6	TcutPNO=1e-7
c_c	12	-7.891	-8.009	-8.026	-8.385	-8.971	-7.787	-7.863
c2_c2	36	-16.699	-17.017	-17.075	-17.815	-19.179	-16.511	-16.696
c3_c1	36	-16.715	-16.926	-17.000	-17.463	-18.789	-16.497	-16.818
c3_c3	48	-25.682	-26.217	-26.361	-27.506	-29.636	-25.425	-25.918
c4_c2	48	-25.588	-26.048	-26.188	-26.918	-29.213	-25.213	-25.769
cc_c	48	-24.452	-24.682	-24.801	-25.481	-27.605		
c5_c1	48	-18.087	-18.332	-18.478	-19.016	-20.465		
c4_c4	60	-34.640	-35.452	-35.670	-36.828	-40.177	-34.065	-34.767
c5_c3	60		-36.121	-36.361	-37.564	-40.655	-35.054	-35.828
c6_c2	60			-28.390	-29.256	-31.349		
c7_c1	60			-18.840	-18.879	-20.847		
cc_cc	72			-54.322	-56.332	-61.586		
c5_c5	72			-45.007	-46.700	-51.115	-43.150	
c6_c4	72			-45.222	-46.602	-50.789	-43.298	
c7_c3	72			-38.445	-39.672	-42.974		
c8_c2	72			-28.872	-29.691	-31.939		
c9_c1	72			-19.066	-19.538	-21.138		
c6_c6	84			-54.350	-56.276	-61.500	-52.178	
c7_c5	84				-59.009	-64.169		
c8_c4	84				-50.250	-54.603		
ccc_c	84				-29.201	-31.488		
ccc_cc	108				-95.237	-104.645		
ccc_ccc	144				-146.575			

Table S28: CCSD correlation part of the interaction energies for different acene dimers for the cc-pVTZ basis set in millihartree (not cp-corrected).

TZ		CCSD cor	LNO-CCSD cor				DLPNO-CCSD Tight	
System	Atoms	canonical	vvTight	vTight	Tight	Normal	TcutPNO=1e-6	TcutPNO=1e-7
c_c	12	-6.491	-6.668	-6.666	-6.730	-6.572	-6.711	-6.634
c2_c2	36	-13.634	-14.061	-14.081	-14.219	-14.022	-14.221	-14.043
c3_c1	36	-13.673	-13.712	-14.042	-14.071	-13.929	-14.267	-14.184
c3_c3	48	-20.884	-21.557	-21.590	-21.817	-21.512	-22.041	-21.921
c4_c2	48	-20.816	-21.423	-21.474	-21.505	-21.395	-21.833	-21.774
cc_c	48						-20.944	-20.778
c5_c1	48						-15.711	
c4_c4	60	-28.090	-29.054	-29.100	-29.180	-29.078	-29.631	-29.484
c5_c3	60		-29.610	-29.688	-29.862	-29.649	-30.474	-30.359
c7_c1	60						-16.096	-16.043
c5_c5	72			-36.626	-36.835	-36.858	-37.654	
c6_c4	72			-36.811	-36.910	-36.899	-37.747	
c7_c3	72						-32.627	
c6_c6	84			-44.112	-44.366	-44.409	-45.548	
c7_c5	84			-46.482	-46.694	-46.768		

Table S29: CCSD(T) correlation part of the interaction energies for different acene dimers for the aug-cc-pVTZ basis set in millihartree (not cp-corrected).

aTZ		CCSD(T) cor	LNO-CCSD(T) cor				DLPNO-CCSD(T) Tight	
System	Atoms	canonical	vvTight	vTight	Tight	Normal	TcutPNO=1e-6	TcutPNO=1e-7
c_c	12	-9.429	-9.513	-9.552	-9.739	-10.373	-9.253	-9.385
c2_c2	36	-19.937	-20.175	-20.246	-20.709	-22.227	-19.760	-19.945
c3_c1	36	-19.597	-19.781	-19.881	-20.201	-21.289	-19.347	-19.654
c3_c3	48			-31.154	-31.889	-34.020	-30.096	
c4_c2	48			-30.630	-31.232	-32.485	-29.764	-30.252
cc_c	48			-29.025	-29.592	-31.644		
c5_c1	48			-21.665	-22.090	-23.358		
c4_c4	60			-42.079	-43.047	-46.138	-40.399	
c5_c3	60			-42.469	-43.296	-46.086	-40.968	
c6_c2	60			-33.212	-33.866	-36.049		
c7_c1	60			-22.184	-22.586	-24.005		
cc_cc	72				-65.720	-70.536		
c5_c5	72				-54.365	-58.439		
c6_c4	72				-54.033	-57.773	-50.719	
c7_c3	72				-45.899	-49.110		
c8_c2	72				-34.529	-36.795		
c9_c1	72				-22.697	-24.581		
c6_c6	84				-65.605	-70.756		
c7_c5	84				-68.000	-73.096		
c8_c4	84				-58.249	-62.526		
ccc_c	84				-34.323	-37.133		

Table S30: CCSD correlation part of the interaction energies for different acene dimers for the aug-cc-pVTZ basis set in millihartree (not cp-corrected).

aTZ		CCSD cor	LNO-CCSD cor				DLPNO-CCSD Tight	
System	Atoms	canonical	vvTight	vTight	Tight	Normal	TcutPNO=1e-6	TcutPNO=1e-7
c_c	12	-7.811	-7.990	-8.015	-8.045	-7.881	-8.040	-7.964
c2_c2	36	-16.449	-16.842	-16.884	-16.937	-16.813	-17.176	-16.946
c3_c1	36	-16.191	-16.538	-16.601	-16.596	-16.325	-16.847	-16.693
c3_c3	48			-25.834	-25.896	-25.637	-26.273	
c4_c2	48			-25.417	-25.436	-22.959	-25.983	-25.758
c4_c4	60			-34.749	-34.863	-34.641	-35.363	
c5_c3	60			-35.096	-35.120	-34.864	-35.858	
c5_c5	72			-43.674	-43.866	-43.825		
c6_c4	72				-43.643	-43.390	-44.434	
c6_c6	84				-52.764	-52.702		
c7_c5	84				-54.850	-54.968		

Table S31: CCSD(T) correlation part of the interaction energies for different acene dimers for the cc-pVQZ basis set in millihartree (not cp-corrected).

QZ		CCSD(T) cor	LNO-CCSD(T) cor				DLPNO-CCSD(T) Tight	
System	Atoms	canonical	vvTight	vTight	Tight	Normal	TcutPNO=1e-6	TcutPNO=1e-7
c_c	12	-8.112	-8.221	-8.266	-8.629	-9.251	-8.007	-8.096
c2_c2	36	-16.912	-17.191	-17.281	-17.976	-19.467	-16.630	-16.821
c3_c1	36		-17.044	-17.135	-17.492	-18.858		
c3_c3	48			-26.512	-27.475	-29.640		
c4_c2	48			-26.243	-26.851	-29.078		
cc_c	48			-24.886	-25.661	-27.614		
c5_c1	48			-18.568	-19.087	-20.500		
c4_c4	60			-35.751	-37.035	-40.120		
c5_c3	60				-37.391	-40.392		
c6_c2	60				-29.202	-31.650		
c7_c1	60				-19.455	-20.955		
cc_cc	72				-56.305	-61.118		
c5_c5	72				-46.676	-50.812		
c6_c4	72				-46.550	-50.550		
c7_c3	72				-39.552	-42.812		
c8_c2	72				-29.735	-32.036		
c9_c1	72				-19.650	-21.124		
c6_c6	84				-56.213	-61.556		
c7_c5	84				-58.816	-63.811		
c8_c4	84				-50.188	-54.489		
ccc_c	84				-29.307	-31.984		

Table S32: CCSD correlation part of the interaction energies for different acene dimers for the cc-pVQZ basis set in millihartree (not cp-corrected).

TZ		CCSD cor	LNO-CCSD cor				DLPNO-CCSD Tight	
System	Atoms	canonical	vvTight	vTight	Tight	Normal	TcutPNO=1e-6	TcutPNO=1e-7
c_c	12	-6.491	-6.668	-6.666	-6.730	-6.572	-6.711	-6.634
c2_c2	36	-13.634	-14.061	-14.081	-14.219	-14.022	-14.221	-14.043
c3_c1	36	-13.673	-13.712	-14.042	-14.071	-13.929	-14.267	-14.184
c3_c3	48	-20.884	-21.557	-21.590	-21.817	-21.512	-22.041	-21.921
c4_c2	48	-20.816	-21.423	-21.474	-21.505	-21.395	-21.833	-21.774
cc_c	48						-20.944	-20.778
c5_c1	48						-15.711	
c4_c4	60	-28.090	-29.054	-29.100	-29.180	-29.078	-29.631	-29.484
c5_c3	60		-29.610	-29.688	-29.862	-29.649	-30.474	-30.359
c7_c1	60						-16.096	-16.043
c5_c5	72			-36.626	-36.835	-36.858	-37.654	
c6_c4	72			-36.811	-36.910	-36.899	-37.747	
c7_c3	72						-32.627	
c6_c6	84			-44.112	-44.366	-44.409	-45.548	
c7_c5	84			-46.482	-46.694	-46.768		

Table S33: CCSD(T) correlation part of the interaction energies for different acene dimers for the aug-cc-pVQZ basis set in millihartree (not cp-corrected).

aQZ		CCSD(T) cor	LNO-CCSD(T) cor			
System	Atoms	canonical	vvTight	vTight	Tight	Normal
c_c	12	-8.631	-8.713	-8.761	-8.969	-9.640
c2_c2	36			-18.167	-18.633	-20.002
c3_c1	36			-17.903	-18.277	-19.416
c3_c3	48			-27.656	-28.561	-30.776
c4_c2	48			-27.367	-28.116	-30.285
cc_c	48			-24.672	-26.416	-28.424
c5_c1	48			-19.392	-19.957	-21.295
c4_c4	60			-37.174	-38.134	-41.704
c5_c3	60				-38.769	-41.638
c6_c2	60				-30.435	-32.709
c7_c1	60				-20.207	-21.674
cc_cc	72				-58.062	-62.318
c5_c5	72				-48.112	-52.130
c6_c4	72				-48.055	-52.171
c7_c3	72				-40.965	-44.132
c8_c2	72				-30.892	-32.846
c9_c1	72				-20.384	-21.858
c6_c6	84				-58.269	-62.894
c7_c5	84				-60.715	-65.702
c8_c4	84				-51.874	-56.266

Table S34: CCSD correlation part of the interaction energies for different acene dimers for the aug-cc-pVQZ basis set in millihartree (not cp-corrected).

aQZ		CCSD cor	LNO-CCSD cor			
System	Atoms	canonical	vvTight	vTight	Tight	Normal
c_c	12	-7.045	-7.224	-7.251	-7.298	-7.118
c2_c2	36			-14.918	-14.952	-14.651
c3_c1	36			-14.744	-14.770	-14.519
c3_c3	48			-22.571	-22.705	-22.532
c4_c2	48			-22.355	-22.417	-22.310
c4_c4	60			-30.174	-30.234	-30.234
c5_c3	60				-30.796	-30.525
c5_c5	72				-37.974	-37.747
c6_c4	72				-37.977	-38.049
c6_c6	84				-45.765	-45.276
c7_c5	84				-47.891	-47.889

Table S35: CCSD(T) correlation part of the interaction energies for different acene dimers for the cc-pV5Z basis set in millihartree (not cp-corrected).

5Z		CCSD(T) cor	LNO-CCSD(T) cor			
System	Atoms	canonical	vvTight	vTight	Tight	Normal
c_c	12	-8.260	-8.368	-8.403	-8.715	-9.384
c2_c2	36			-17.450	-18.080	-19.556
c3_c1	36			-17.226	-17.651	-18.877
c3_c3	48			-26.656	-27.588	-29.925
c4_c2	48			-26.355	-27.069	-29.299
cc_c	48			-24.903	-25.608	-27.702
c5_c1	48				-19.104	-20.587
c4_c4	60				-36.977	-40.260
c5_c3	60				-37.445	-40.541
c6_c2	60				-29.320	-31.582
c7_c1	60				-19.534	-20.982
cc_cc	72				-56.141	-61.248
c5_c5	72				-46.790	-50.740
c6_c4	72				-46.812	-50.663
c7_c3	72				-39.591	-42.781
c8_c2	72				-29.467	-32.050
c9_c1	72				-19.722	-21.261
c6_c6	84				-56.559	-61.349
c7_c5	84				-58.968	-63.841
c8_c4	84				-50.324	-54.289

Table S36: CCSD correlation part of the interaction energies for different acene dimers for the cc-pV5Z basis set in millihartree (not cp-corrected).

5Z		CCSD cor	LNO-CCSD cor			
System	Atoms	canonical	vvTight	vTight	Tight	Normal
c_c	12	-6.713	-6.904	-6.910	-7.010	-6.818
c2_c2	36			-14.257	-14.373	-14.164
c3_c1	36			-14.120	-14.142	-13.922
c3_c3	48			-21.633	-21.768	-21.675
c4_c2	48			-21.411	-21.428	-21.328
c4_c4	60			-28.967	-29.053	-29.034
c5_c3	60				-29.517	-29.476
c5_c5	72				-36.603	-36.549
c6_c4	72				-36.661	-36.654
c6_c6	84				-44.075	-44.109
c7_c5	84				-46.176	-46.328

Table S37: CCSD(T) correlation part of the interaction energies for different acene dimers for the aug-cc-pV5Z basis set in millihartree (not cp-corrected).

a5Z		CCSD(T) cor	LNO-CCSD(T) cor			
System	Atoms	canonical	vvTight	vTight	Tight	Normal
c_c	12		-8.505	-8.558	-8.745	-9.306
c2_c2	36			-17.649	-18.171	-19.386
c3_c1	36			-17.408	-17.710	-18.909
c3_c3	48			-26.865	-27.701	-29.911
c4_c2	48				-27.140	-29.422
cc_c	48				-25.614	-27.612
c5_c1	48				-19.298	-21.029
c4_c4	60				-36.986	-40.590
c5_c3	60				-37.733	-40.436
c6_c2	60				-29.414	-32.090
c7_c1	60				-19.754	-21.464
cc_cc	72				-55.895	-60.846
c5_c5	72				-47.357	-51.736
c6_c4	72				-46.671	-50.929
c7_c3	72				-39.957	-43.468
c8_c2	72				-29.949	-32.317
c9_c1	72				-19.848	-21.206
c6_c6	84				-56.451	-61.291
c7_c5	84				-59.430	-64.722
c8_c4	84				-50.580	-55.222

Table S38: CCSD correlation part of the interaction energies for different acene dimers for the aug-cc-pV5Z basis set in millihartree (not cp-corrected).

a5Z		CCSD cor	LNO-CCSD cor			
System	Atoms	canonical	vvTight	vTight	Tight	Normal
c_c	12		-7.031	-7.066	-7.075	-6.857
c2_c2	36			-14.427	-14.539	-14.229
c3_c1	36			-14.274	-14.246	-14.083
c3_c3	48			-21.807	-21.976	-21.803
c4_c2	48				-21.585	-21.645
c4_c4	60				-29.160	-29.267
c5_c3	60				-29.866	-29.498
c5_c5	72				-37.108	-37.252
c6_c4	72				-36.680	-36.916
c6_c6	84				-44.115	-44.025
c7_c5	84				-46.642	-46.854

Table S39: Correlation part of the interaction energies for different acene$_x$ dimers and (extrapolated) basis sets (cp-corrected) in kJ/mol using LNO-CCSD(T) with tight criteria.

	DZ	aDZ	TZ	DTZ	aTZ	aDTZ	QZ	TQZ	aQZ	aTQZ	5Z	Q5Z	a5Z	aQ5Z
1	-17.22	-21.85	-20.62	-22.05	-22.28	-22.47	-22.08	-23.15	-22.29	-22.29	-22.64	-23.23	-22.69	-23.10
2	-35.69	-45.43	-44.05	-47.57	-46.24	-46.59	-45.57	-46.68	-46.47	-46.64	-46.65	-47.79	-46.57	-46.68
3	-55.01	-69.86	-66.95	-71.98	-70.81	-71.22	-69.69	-71.69	-71.19	-71.47	-71.08	-72.53	-71.10	-71.00
4	-73.43	-93.59	-88.49	-94.84	-95.16	-95.83	-93.79	-97.65	-95.20	-95.22	-95.18	-96.64	-95.49	-95.80
5	-92.66	-117.89	-112.50	-120.86	-120.30	-121.32	-118.21	-122.37	-120.40	-120.48	-120.35	-122.59	-121.13	-121.88
6	-112.54	-142.25	-135.47	-145.12	-146.35	-148.08	-142.27	-147.23	-145.29	-144.52	-145.26	-148.40	-145.56	-145.85

Table S40: Correlation part of the interaction energies for different acene$_x$ dimers and (extrapolated) basis sets (cp-corrected) in kJ/mol using LNO-CCSD(T) with very tight criteria.

	DZ	aDZ	TZ	DTZ	aTZ	aDTZ	QZ	TQZ	aQZ	aTQZ	5Z	Q5Z	a5Z	aQ5Z
1	-15.77	-21.49	-19.32	-20.81	-21.76	-21.87	-20.93	-22.10	-21.91	-22.02	-21.85	-22.81	-22.08	-22.27
2	-33.01	-44.12	-40.59	-43.79	-45.04	-45.43	-43.55	-45.71	-45.29	-45.46	-44.83	-46.17	-45.37	-45.46
3	-50.49	-67.47	-62.41	-67.43	-68.72	-69.24	-66.75	-69.91	-69.04	-69.28	-68.54	-70.42	-69.14	-69.24
4	-68.16	-90.78	-84.38	-91.20	-92.45	-93.16	-89.96	-94.04	-92.83	-93.10				
5	-86.53	-114.16	-106.43	-114.81										
6	-104.68		-137.25	-150.97										

Table S41: Correlation part of the interaction energies for different acene$_x$ dimers and (extrapolated) basis sets (non-cp-corrected) in kJ/mol using LNO-CCSD(T) with tight criteria.

	DZ	TZ	DTZ	aTZ	QZ	TQZ	aQZ	aTQZ	5Z	Q5Z	a5Z	aQ5Z
1	-17.93	-22.01	-23.73	-25.57	-22.66	-23.12	-23.55	-22.07	-22.88	-23.12	-22.96	-22.34
2	-38.88	-46.77	-50.10	-54.37	-47.20	-47.50	-48.92	-44.94	-47.47	-47.76	-47.71	-46.44
3	-60.65	-72.22	-77.09	-83.72	-72.14	-72.08	-74.99	-68.61	-72.43	-72.75	-72.73	-70.36
4	-82.55	-96.69	-102.64	-113.02	-97.24	-97.63	-100.12	-90.71	-97.08	-96.93	-97.11	-93.95
5	-104.62	-122.61	-130.19	-142.73	-122.55	-122.50	-126.32	-114.34	-122.85	-123.16	-124.34	-122.26
6	-126.34	-147.75	-156.77	-172.24	-147.59	-147.47	-152.99	-138.93	-148.50	-149.45	-148.21	-143.20

Table S42: Correlation part of the interaction energies for different acene dimers for the cc-pVDZ basis set in millihartree (cp-corrected).

DZ		PNO-LCCSD		PNO-LCCSD(T)	
System	Rings	Tight	vTight	Tight	vTight
c1_c1	1	-4.835	-4.881	-5.626	-5.700
c2_c2	2	-10.022	-10.113	-11.747	-11.918
c3_c3	3	-15.187	-15.347	-17.847	-18.150
c4_c4	4	-20.563	-20.754	-24.192	-24.570
c5_c5	5	-25.805	-26.103	-30.355	-30.927
c6_c6	6	-31.140	-31.436	-36.661	-37.267

Table S43: Correlation part of the interaction energies for different acene dimers for the cc-pVDZ basis set in millihartree (not cp-corrected).

DZ		PNO-LCCSD		PNO-LCCSD(T)	
System	Rings	Tight	vTight	Tight	vTight
c1_c1	1	-5.158	-5.217	-6.067	-6.160
c2_c2	2	-10.842	-10.935	-12.827	-13.013
c3_c3	3	-16.390	-16.575	-19.419	-19.760
c4_c4	4	-22.394	-22.597	-26.559	-26.969
c5_c5	5	-28.088	-28.379	-33.268	-33.848
c6_c6	6	-33.777	-34.075	-40.020	-40.643

Table S44: Correlation part of the interaction energies for different Polyene stack dimers in Relaxed geometry for the cc-pVDZ basis set in millihartree (cp-corrected).

DZ		CCSD(T) cor	LNO-CCSD(T) cor				DLPNO-CCSD(T) Tight	
System	D-Bonds	canonical	vvTight	vTight	Tight	Normal	TcutPNO=1e-6	TcutPNO=1e-7
c2_c2	2	-0.677	-0.684	-0.687	-0.711	-0.723	-0.701	-0.699
c4_c4	4	-2.765	-2.810	-2.805	-2.989	-3.210	-2.785	-2.842
c6_c6	6	-5.018	-5.110	-5.129	-5.420	-5.829	-5.070	-5.196
c8_c8	8	-7.494	-7.630	-7.677	-8.079	-8.801	-7.572	-7.739
c10_c10	10	-9.850	-10.081	-10.134	-10.769	-11.636	-9.959	-10.166
c12_c12	12	-12.079	-12.364	-12.454	-13.176	-14.273	-12.220	-12.464
c14_c14	14	-14.816	-15.186	-15.282	-16.170	-17.535	-14.881	-15.201

Table S45: Correlation part of the interaction energies for different Polyene stack dimers in Relaxed geometry for the aug-cc-pVDZ basis set in millihartree (cp-corrected).

aDZ		CCSD(T) cor	LNO-CCSD(T) cor				DLPNO-CCSD(T) Tight	
System	D-Bonds	canonical	vvTight	vTight	Tight	Normal	TcutPNO=1e-6	TcutPNO=1e-7
c2_c2	2	-1.084	-1.090	-1.087	-1.119	-1.313	-1.106	-1.108
c4_c4	4	-4.044	-4.060	-4.057	-4.181	-4.358	-3.957	-3.995
c6_c6	6	-7.186	-7.252	-7.265	-7.421	-7.747	-7.009	-7.145
c8_c8	8		-10.909	-10.905	-10.884	-11.574	-10.299	-10.528
c10_c10	10		-14.045	-14.047	-14.267	-15.196	-13.559	-13.852
c12_c12	12		-17.213	-17.205	-17.508	-18.589	-16.614	-16.982
c14_c14	14		-20.980	-20.969	-21.340	-22.678		-20.725

Table S46: Correlation part of the interaction energies for different Polyene stack dimers in Relaxed geometry for the cc-pVTZ basis set in millihartree (cp-corrected).

TZ		CCSD(T) cor	LNO-CCSD(T) cor				DLPNO-CCSD(T) Tight	
System	D-Bonds	canonical	vvTight	vTight	Tight	Normal	TcutPNO=1e-6	TcutPNO=1e-7
c2_c2	2	-0.922	-0.929	-0.927	-0.973	-1.164	-0.966	-0.987
c4_c4	4	-3.572	-3.615	-3.597	-3.833	-4.329	-3.601	-3.651
c6_c6	6	-6.417	-6.522	-6.524	-6.877	-7.587	-6.382	-6.509
c8_c8	8	-9.524	-9.735	-9.706	-9.711	-11.235	-9.383	-9.620
c10_c10	10	-12.485	-12.794	-12.740	-13.461	-14.838	-12.263	-12.601
c12_c12	12	-15.284	-15.657	-15.618	-16.582	-18.231	-15.038	-15.461
c14_c14	14	-18.697	-19.106	-19.111	-20.248	-22.231	-18.368	-18.889

Table S47: Correlation part of the interaction energies for different Polyene stack dimers in Relaxed geometry for the aug-cc-pVTZ basis set in millihartree (cp-corrected).

aTZ		CCSD(T) cor	LNO-CCSD(T) cor				DLPNO-CCSD(T) Tight	
System	D-Bonds	canonical	vvTight	vTight	Tight	Normal	TcutPNO=1e-6	TcutPNO=1e-7
c2_c2	2	-1.108	-1.110	-1.110	-1.137	-1.406	-1.138	-1.135
c4_c4	4	-4.115	-4.141	-4.146	-4.230	-4.483	-4.087	-4.106
c6_c6	6	-7.301	-7.376	-7.402	-7.549	-7.865	-7.204	-7.296
c8_c8	8		-10.909	-10.905	-11.068	-11.647	-10.564	
c10_c10	10		-14.282	-14.260	-14.563	-15.373	-13.804	-14.090
c12_c12	12		-17.498	-17.460	-17.907	-18.904		
c14_c14	14		-21.353	-21.304	-21.771	-23.000	-20.633	-21.136

Table S48: Correlation part of the interaction energies for different Polyene stack dimers in Relaxed geometry for the cc-pVQZ basis set in millihartree (cp-corrected).

QZ		CCSD(T) cor	LNO-CCSD(T) cor				DLPNO-CCSD(T) Tight	
System	D-Bonds	canonical	vvTight	vTight	Tight	Normal	TcutPNO=1e-6	TcutPNO=1e-7
c2_c2	2	-1.026	-1.027	-1.025	-1.109	-1.181	-1.036	-1.039
c4_c4	4	-3.898	-3.946	-3.931	-4.132	-4.377	-3.879	-3.909
c6_c6	6	-6.968	-7.080	-7.084	-7.414	-7.886	-6.897	-6.987
c8_c8	8	-10.317	-10.510	-10.480	-10.984	-11.692	-10.112	-10.322
c10_c10	10	-13.503	-13.785	-13.752	-14.400	-15.335	-13.274	-13.542
c12_c12	12		-16.876	-16.829	-17.651	-18.859	-16.253	-16.566
c14_c14	14		-20.667	-20.567	-21.502	-23.072	-19.811	-20.209

Table S49: Correlation part of the interaction energies for different Polyene stack dimers in Relaxed geometry for the aug-cc-pVQZ basis set in millihartree (cp-corrected).

aQZ		CCSD(T) cor	LNO-CCSD(T) cor				DLPNO-CCSD(T) Tight	
System	D-Bonds	canonical	vvTight	vTight	Tight	Normal	TcutPNO=1e-6	TcutPNO=1e-7
c2_c2	2	-1.112	-1.118	-1.116	-1.164	-1.260	-1.144	-1.143
c4_c4	4	-4.138	-4.173	-4.166	-4.221	-4.459	-4.114	-4.153
c6_c6	6		-7.431	-7.462	-7.620	-7.992	-7.227	
c8_c8	8		-10.983	-10.995	-11.208	-11.884	-10.605	-10.813
c10_c10	10			-14.379	-14.660	-15.559	-13.888	
c12_c12	12			-17.571	-17.961	-19.047		
c14_c14	14			-21.452	-21.899	-23.227		

Table S50: Correlation part of the interaction energies for different Polyene stack dimers in Relaxed geometry for the cc-pV5Z basis set in millihartree (cp-corrected).

5Z		CCSD(T) cor	LNO-CCSD(T) cor				DLPNO-CCSD(T) Tight	
System	D-Bonds	canonical	vvTight	vTight	Tight	Normal	TcutPNO=1e-6	TcutPNO=1e-7
c2_c2	2	-1.077	-1.083	-1.086	-1.130	-1.245	-1.120	-1.114
c4_c4	4	-4.057	-4.106	-4.097	-4.255	-4.502	-4.074	-4.072
c6_c6	6		-7.316	-7.342	-7.610	-8.091		
c8_c8	8		-10.852	-10.855	-11.224	-11.967		

Table S51: Correlation part of the interaction energies for different Polyene stack dimers in Relaxed geometry for the aug-cc-pV5Z basis set in millihartree (cp-corrected).

a5Z		CCSD(T) cor	LNO-CCSD(T) cor				DLPNO-CCSD(T) Tight	
System	D-Bonds	canonical	vvTight	vTight	Tight	Normal	TcutPNO=1e-6	TcutPNO=1e-7
c2_c2	2	-1.117	-1.128	-1.117	-1.164	-1.232	-1.151	-1.156
c4_c4	4	-4.149	-4.186	-4.178	-4.272	-4.565	-4.137	-4.173
c6_c6	6		-7.445	-7.477	-7.645	-8.060		
c8_c8	8		-10.997	-11.009	-11.264	-12.082		

Table S52: Correlation part of the interaction energies for different Polyene stack dimers in Fixed geometry for the cc-pVDZ basis set in millihartree (cp-corrected).

DZ		CCSD(T) cor	LNO-CCSD(T) cor				DLPNO-CCSD(T) Tight	
System	D-Bonds	canonical	vvTight	vTight	Tight	Normal	TcutPNO=1e-6	TcutPNO=1e-7
c2_c2	2	-0.677	-0.684	-0.687	-0.711	-0.723	-0.701	-0.699
c4_c4	4	-1.560	-1.576	-1.594	-1.709	-2.028	-1.638	-1.665
c6_c6	6	-2.531	-2.583	-2.623	-2.829	-3.067	-2.684	-2.743
c8_c8	8	-3.534	-3.640	-3.707	-3.992	-0.822	-3.764	-3.863
c10_c10	10	-4.555	-4.713	-4.789	-5.208	-5.568	-4.852	-4.997
c12_c12	12	-5.582	-5.780	-5.878	-6.376	-6.861	-5.962	-6.119
c14_c14	14	-6.615	-6.848	-6.966	-7.542	-8.161	-7.105	-7.283

Table S53: Correlation part of the interaction energies for different Polyene stack dimers in Fixed geometry for the aug-cc-pVDZ basis set in millihartree (cp-corrected).

aDZ		CCSD(T) cor	LNO-CCSD(T) cor				DLPNO-CCSD(T) Tight	
System	D-Bonds	canonical	vvTight	vTight	Tight	Normal	TcutPNO=1e-6	TcutPNO=1e-7
c2_c2	2	-1.084	-1.090	-1.087	-1.119	-1.313	-1.106	-1.108
c4_c4	4	-2.418	-2.440	-2.442	-2.578	-2.966	-2.484	-2.520
c6_c6	6	-3.862	-3.897	-3.931	-4.121	-4.495		
c8_c8	8		-5.430	-5.481	-5.683	-6.213		
c10_c10	10		-6.982	-7.041	-7.361	-7.879		
c12_c12	12		-8.508	-8.593	-8.944	-9.610		
c14_c14	14		-10.114	-10.176	-10.575	-11.458		

Table S54: Correlation part of the interaction energies for different Polyene stack dimers in Fixed geometry for the cc-pVTZ basis set in millihartree (cp-corrected).

TZ		CCSD(T) cor	LNO-CCSD(T) cor				DLPNO-CCSD(T) Tight	
System	D-Bonds	canonical	vvTight	vTight	Tight	Normal	TcutPNO=1e-6	TcutPNO=1e-7
c2_c2	2	-0.922	-0.929	-0.927	-0.973	-1.164	-0.966	-0.987
c4_c4	4	-2.070	-2.114	-2.117	-2.323	-2.625	-2.160	-2.212
c6_c6	6	-3.329	-3.428	-3.462	-3.724	-4.074	-3.497	-3.601
c8_c8	8	-4.625	-4.771	-4.816	-5.262	-5.512	-4.845	-5.021
c10_c10	10	-5.940	-6.171	-6.235	-6.667	-7.217	-6.273	-6.481
c12_c12	12	-7.262	-7.556	-7.640	-8.169	-8.948	-7.675	-7.943
c14_c14	14	-8.590	-8.892	-9.013	-9.699	-10.600	-9.098	-9.421

Table S55: Correlation part of the interaction energies for different Polyene stack dimers in Fixed geometry for the aug-cc-pVTZ basis set in millihartree (cp-corrected).

aTZ		CCSD(T) cor	LNO-CCSD(T) cor				DLPNO-CCSD(T) Tight	
System	D-Bonds	canonical	vvTight	vTight	Tight	Normal	TcutPNO=1e-6	TcutPNO=1e-7
c2_c2	2	-1.108	-1.110	-1.110	-1.137	-1.406	-1.138	-1.135
c4_c4	4	-2.434	-2.453	-2.461	-2.582	-2.974	-2.510	-2.521
c6_c6	6	-3.870	-3.916	-3.956	-4.115	-4.341		
c8_c8	8		-5.430	-5.476	-5.672	-6.055		
c10_c10	10		-6.959	-7.004	-7.297	-7.880		
c12_c12	12		-8.513	-8.574	-8.928	-9.665		
c14_c14	14		-10.077	-10.146	-10.591	-11.465		

Table S56: Correlation part of the interaction energies for different Polyene stack dimers in Fixed geometry for the cc-pVQZ basis set in millihartree (cp-corrected).

QZ		CCSD(T) cor	LNO-CCSD(T) cor				DLPNO-CCSD(T) Tight	
System	D-Bonds	canonical	vvTight	vTight	Tight	Normal	TcutPNO=1e-6	TcutPNO=1e-7
c2_c2	2	-1.026	-1.027	-1.025	-1.109	-1.181	-1.036	-1.039
c4_c4	4	-2.281	-2.305	-2.310	-2.556	-2.854	-2.337	-2.365
c6_c6	6	-3.651	-3.714	-3.744	-4.082	-4.529	-3.822	-3.888
c8_c8	8	-5.059	-5.178	-5.221	-5.673	-6.123	-5.304	-5.408
c10_c10	10	-6.488	-6.655	-6.715	-7.237	-7.740	-6.819	-6.968
c12_c12	12		-8.185	-8.256	-8.928	-8.609	-8.352	-8.531
c14_c14	14		-9.675	-9.768	-10.442	-10.953	-9.891	-10.107

Table S57: Correlation part of the interaction energies for different Polyene stack dimers in Fixed geometry for the aug-cc-pVQZ basis set in millihartree (cp-corrected).

aQZ		CCSD(T) cor	LNO-CCSD(T) cor				DLPNO-CCSD(T) Tight	
System	D-Bonds	canonical	vvTight	vTight	Tight	Normal	TcutPNO=1e-6	TcutPNO=1e-7
c2_c2	2	-1.112	-1.118	-1.116	-1.164	-1.260	-1.144	-1.143
c4_c4	4	-2.432	-2.456	-2.462	-2.595	-2.724	-2.524	-2.530
c6_c6	6		-3.933	-3.966	-4.165	-4.436		-4.072
c8_c8	8		-5.439	-5.489	-5.727	-6.019		
c10_c10	10		-6.969	-7.036	-7.343	-7.900		
c12_c12	12		-8.545	-8.587	-8.973	-9.680		
c14_c14	14			-10.131	-10.601	-11.462		

Table S58: Correlation part of the interaction energies for different Polyene stack dimers in Fixed geometry for the cc-pV5Z basis set in millihartree (cp-corrected).

5Z		CCSD(T) cor	LNO-CCSD(T) cor				DLPNO-CCSD(T) Tight	
System	D-Bonds	canonical	vvTight	vTight	Tight	Normal	TcutPNO=1e-6	TcutPNO=1e-7
c2_c2	2	-1.077	-1.083	-1.086	-1.130	-1.245	-1.120	-1.114
c4_c4	4	-2.373	-2.402	-2.404	-2.589	-2.725	-2.485	-2.483
c6_c6	6		-3.846	-3.887	-4.176	-4.537		
c8_c8	8		-5.335	-5.398	-5.823	-6.362		

Table S59: Correlation part of the interaction energies for different Polyene stack dimers in Fixed geometry for the aug-cc-pV5Z basis set in millihartree (cp-corrected).

a5Z		CCSD(T) cor	LNO-CCSD(T) cor				DLPNO-CCSD(T) Tight	
System	D-Bonds	canonical	vvTight	vTight	Tight	Normal	TcutPNO=1e-6	TcutPNO=1e-7
c2_c2	2	-1.117	-1.128	-1.117	-1.164	-1.232	-1.151	-1.156
c4_c4	4	-2.436	-2.466	-2.464	-2.597	-2.815	-2.524	-2.540
c6_c6	6		-3.922	-3.957	-4.165	-4.477		
c8_c8	8		-5.428	-5.484	-5.747	-6.401		

Table S60: Correlation part of the interaction energies for different Polyene stack dimers in Relaxed geometry for the cc-pVDZ basis set in millihartree (not cp-corrected).

DZ		CCSD(T) cor	LNO-CCSD(T) cor				DLPNO-CCSD(T) Tight	
System	D-Bonds	canonical	vvTight	vTight	Tight	Normal	TcutPNO=1e-6	TcutPNO=1e-7
c2_c2	2	-0.624	-0.626	-0.628	-0.657	-0.688	-0.632	-0.631
c4_c4	4	-2.807	-2.883	-2.880	-3.023	-3.172	-2.799	-2.827
c6_c6	6	-5.269	-5.380	-5.396	-5.578	-5.870	-5.265	-5.359
c8_c8	8	-8.017	-8.199	-8.247	-8.462	-8.942	-7.998	-8.117
c10_c10	10	-10.632	-10.884	-10.951	-11.276	-11.967	-10.593	-10.729
c12_c12	12	-13.082	-13.432	-13.524	-13.944	-14.752	-13.004	-13.186
c14_c14	14	-16.006	-16.467	-16.583	-17.046	-18.128	-15.778	-16.019

Table S61: Correlation part of the interaction energies for different Polyene stack dimers in Relaxed geometry for the aug-cc-pVDZ basis set in millihartree (not cp-corrected).

aDZ		CCSD(T) cor	LNO-CCSD(T) cor				DLPNO-CCSD(T) Tight	
System	D-Bonds	canonical	vvTight	vTight	Tight	Normal	TcutPNO=1e-6	TcutPNO=1e-7
c2_c2	2	-1.412	-1.416	-1.410	-1.434	-1.502	-1.425	-1.427
c4_c4	4	-5.278	-5.271	-5.269	-5.380	-5.624	-5.161	-5.206
c6_c6	6	-9.462	-9.528	-9.541	-9.726	-10.034	-9.140	-9.303
c8_c8	8		-12.604	-12.622	-14.389	-15.034	-13.490	-13.766
c10_c10	10		-18.656	-18.650	-18.964	-19.721	-17.707	-18.045
c12_c12	12		-22.896	-22.866	-23.258	-24.333	-21.645	-22.055
c14_c14	14		-27.745	-27.757	-28.314	-29.495		-26.675

Table S62: Correlation part of the interaction energies for different Polyene stack dimers in Relaxed geometry for the cc-pVTZ basis set in millihartree (not cp-corrected).

TZ		CCSD(T) cor	LNO-CCSD(T) cor				DLPNO-CCSD(T) Tight	
System	D-Bonds	canonical	vvTight	vTight	Tight	Normal	TcutPNO=1e-6	TcutPNO=1e-7
c2_c2	2	-0.904	-0.910	-0.909	-0.947	-1.072	-0.935	-0.958
c4_c4	4	-3.777	-3.819	-3.808	-3.965	-4.171	-3.750	-3.809
c6_c6	6	-6.881	-6.985	-6.997	-7.222	-7.634	-6.759	-6.866
c8_c8	8	-10.283	-10.479	-10.470	-10.814	-11.398	-10.003	-10.194
c10_c10	10	-13.523	-13.801	-13.771	-14.205	-15.008	-13.077	-13.356
c12_c12	12	-16.578	-16.903	-16.890	-17.444	-18.515	-16.029	-16.383
c14_c14	14	-20.221	-20.561	-20.590	-21.204	-22.537	-19.516	-19.967

Table S63: Correlation part of the interaction energies for different Polyene stack dimers in Relaxed geometry for the aug-cc-pVTZ basis set in millihartree (not cp-corrected).

aTZ		CCSD(T) cor	LNO-CCSD(T) cor				DLPNO-CCSD(T) Tight	
System	D-Bonds	canonical	vvTight	vTight	Tight	Normal	TcutPNO=1e-6	TcutPNO=1e-7
c2_c2	2	-1.236	-1.237	-1.239	-1.281	-1.357	-1.276	-1.275
c4_c4	4	-4.704	-4.734	-4.744	-4.827	-4.828	-4.688	-4.698
c6_c6	6	-8.413	-8.504	-8.541	-8.688	-8.847	-8.263	-8.322
c8_c8	8		-12.604	-12.622	-12.851	-13.442	-12.162	
c10_c10	10		-16.495	-16.544	-16.803	-17.462	-15.794	-16.007
c12_c12	12		-20.189	-20.268	-20.676	-21.525	-19.267	
c14_c14	14		-24.608	-24.639	-25.052	-26.252	-23.380	-23.804

Table S64: Correlation part of the interaction energies for different Polyene stack dimers in Relaxed geometry for the cc-pVQZ basis set in millihartree (not cp-corrected).

QZ		CCSD(T) cor	LNO-CCSD(T) cor				DLPNO-CCSD(T) Tight	
System	D-Bonds	canonical	vvTight	vTight	Tight	Normal	TcutPNO=1e-6	TcutPNO=1e-7
c2_c2	2	-1.028	-1.032	-1.030	-1.079	-1.140	-1.026	-1.032
c4_c4	4	-4.000	-4.054	-4.042	-4.171	-4.434	-3.952	-3.972
c6_c6	6	-7.185	-7.299	-7.306	-7.563	-7.945	-7.051	-7.127
c8_c8	8	-10.664	-10.857	-10.835	-11.172	-11.808	-10.345	-10.534
c10_c10	10	-13.974	-14.246	-14.226	-14.681	-15.571	-13.606	-13.830
c12_c12	12		-17.440	-17.412	-18.011	-19.121	-16.665	-16.925
c14_c14	14		-21.247	-21.198	-21.842	-23.301	-20.259	-20.587

Table S65: Correlation part of the interaction energies for different Polyene stack dimers in Relaxed geometry for the aug-cc-pVQZ basis set in millihartree (not cp-corrected).

aQZ		CCSD(T) cor	LNO-CCSD(T) cor				DLPNO-CCSD(T) Tight	
System	D-Bonds	canonical	vvTight	vTight	Tight	Normal	TcutPNO=1e-6	TcutPNO=1e-7
c2_c2	2	-1.158	-1.164	-1.160	-1.196	-1.294	-1.209	-1.190
c4_c4	4	-4.320	-4.354	-4.351	-4.451	-4.624	-4.337	-4.346
c6_c6	6		-7.770	-7.799	-7.972	-8.276	-7.613	
c8_c8	8		-11.492	-11.517	-11.780	-12.196	-11.137	-11.289
c10_c10	10			-15.073	-15.425	-16.355	-14.555	
c12_c12	12			-18.422	-18.866	-20.022		
c14_c14	14			-22.407	-22.849	-24.029		

Table S66: Correlation part of the interaction energies for different Polyene stack dimers in Relaxed geometry for the cc-pV5Z basis set in millihartree (not cp-corrected).

5Z		CCSD(T) cor	LNO-CCSD(T) cor				DLPNO-CCSD(T) Tight	
System	D-Bonds	canonical	vvTight	vTight	Tight	Normal	TcutPNO=1e-6	TcutPNO=1e-7
c2_c2	2	-1.093	-1.100	-1.103	-1.140	-1.258	-1.118	-1.109
c4_c4	4	-4.126	-4.174	-4.164	-4.293	-4.524	-4.131	-4.142
c6_c6	6	-7.353	-7.442	-7.474	-7.680	-8.124		
c8_c8	8		-11.052	-11.058	-11.377	-12.037		

Table S67: Correlation part of the interaction energies for different Polyene stack dimers in Relaxed geometry for the aug-cc-pV5Z basis set in millihartree (not cp-corrected).

a5Z		CCSD(T) cor	LNO-CCSD(T) cor				DLPNO-CCSD(T) Tight	
System	D-Bonds	canonical	vvTight	vTight	Tight	Normal	TcutPNO=1e-6	TcutPNO=1e-7
c2_c2	2	-1.138	-1.146	-1.140	-1.164	-1.232	-1.188	-1.186
c4_c4	4	-4.236	-4.273	-4.268	-4.376	-4.647	-4.257	-4.271
c6_c6	6		-7.600	-7.647	-7.820	-8.173		
c8_c8	8		-11.229	-11.253	-11.536	-12.276		

Table S68: Correlation part of the interaction energies for different Polyene stack dimers in Fixed geometry for the cc-pVDZ basis set in millihartree (not cp-corrected).

DZ		CCSD(T) cor	LNO-CCSD(T) cor				DLPNO-CCSD(T) Tight	
System	D-Bonds	canonical	vvTight	vTight	Tight	Normal	TcutPNO=1e-6	TcutPNO=1e-7
c2_c2	2	-0.624	-0.626	-0.628	-0.657	-0.688	-0.632	-0.631
c4_c4	4	-1.513	-1.559	-1.577	-1.671	-1.756	-1.566	-1.590
c6_c6	6	-2.497	-2.584	-2.631	-2.783	-2.930	-2.613	-2.655
c8_c8	8	-3.516	-3.635	-3.700	-3.914	-4.167	-3.687	-3.774
c10_c10	10	-4.552	-4.709	-4.799	-5.121	-5.474	-4.800	-4.890
c12_c12	12	-5.596	-5.815	-5.919	-6.332	-6.752	-5.923	-6.026
c14_c14	14	-6.644	-6.897	-7.049	-7.475	-8.036	-7.035	-7.160

Table S69: Correlation part of the interaction energies for different Polyene stack dimers in Fixed geometry for the aug-cc-pVDZ basis set in millihartree (not cp-corrected).

aDZ		CCSD(T) cor	LNO-CCSD(T) cor				DLPNO-CCSD(T) Tight	
System	D-Bonds	canonical	vvTight	vTight	Tight	Normal	TcutPNO=1e-6	TcutPNO=1e-7
c2_c2	2	-1.412	-1.416	-1.410	-1.434	-1.502	-1.425	-1.427
c4_c4	4	-3.241	-3.240	-3.241	-3.382	-3.532	-3.261	-3.306
c6_c6	6	-5.243	-5.282	-5.318	-5.532	-5.720		
c8_c8	8		-7.453	-7.503	-7.757	-8.070		
c10_c10	10		-9.661	-9.718	-10.104	-10.494		
c12_c12	12		-11.846	-11.952	-12.303	-12.990		
c14_c14	14		-14.122	-14.191	-14.766	-15.515		

Table S70: Correlation part of the interaction energies for different Polyene stack dimers in Fixed geometry for the cc-pVTZ basis set in millihartree (not cp-corrected).

TZ		CCSD(T) cor	LNO-CCSD(T) cor				DLPNO-CCSD(T) Tight	
System	D-Bonds	canonical	vvTight	vTight	Tight	Normal	TcutPNO=1e-6	TcutPNO=1e-7
c2_c2	2	-0.904	-0.910	-0.909	-0.947	-1.072	-0.935	-0.958
c4_c4	4	-2.112	-2.142	-2.146	-2.311	-2.496	-2.172	-2.225
c6_c6	6	-3.439	-3.503	-3.537	-3.766	-4.017	-3.535	-3.638
c8_c8	8	-4.803	-4.916	-4.964	-5.264	-5.628	-4.938	-5.085
c10_c10	10	-6.188	-6.347	-6.408	-6.780	-7.271	-6.367	-6.561
c12_c12	12	-7.580	-7.775	-7.854	-8.344	-8.947	-7.780	-8.040
c14_c14	14	-8.977	-9.192	-9.303	-9.847	-10.579	-9.222	-9.544

Table S71: Correlation part of the interaction energies for different Polyene stack dimers in Fixed geometry for the aug-cc-pVTZ basis set in millihartree (not cp-corrected).

aTZ		CCSD(T) cor	LNO-CCSD(T) cor				DLPNO-CCSD(T) Tight	
System	D-Bonds	canonical	vvTight	vTight	Tight	Normal	TcutPNO=1e-6	TcutPNO=1e-7
c2_c2	2	-1.236	-1.237	-1.239	-1.281	-1.357	-1.276	-1.275
c4_c4	4	-2.843	-2.866	-2.876	-2.994	-3.147	-2.924	-2.928
c6_c6	6	-4.571	-4.631	-4.679	-4.797	-4.995		
c8_c8	8		-6.434	-6.497	-6.760	-6.920		
c10_c10	10		-8.239	-8.341	-8.654	-9.127		
c12_c12	12		-10.110	-10.220	-10.604	-11.202		
c14_c14	14		-11.967	-12.085	-12.500	-13.338		

Table S72: Correlation part of the interaction energies for different Polyene stack dimers in Fixed geometry for the cc-pVQZ basis set in millihartree (not cp-corrected).

QZ		CCSD(T) cor	LNO-CCSD(T) cor				DLPNO-CCSD(T) Tight	
System	D-Bonds	canonical	vvTight	vTight	Tight	Normal	TcutPNO=1e-6	TcutPNO=1e-7
c2_c2	2	-1.028	-1.032	-1.030	-1.079	-1.140	-1.026	-1.032
c4_c4	4	-2.327	-2.353	-2.360	-2.515	-2.797	-2.359	-2.377
c6_c6	6	-3.745	-3.808	-3.838	-4.051	-4.500	-3.824	-3.892
c8_c8	8	-5.204	-5.321	-5.366	-5.628	-6.097	-5.333	-5.425
c10_c10	10	-6.683	-6.847	-6.912	-7.284	-7.779	-6.869	-7.001
c12_c12	12		-8.387	-8.464	-8.965	-9.549	-8.423	-8.593
c14_c14	14		-9.907	-10.013	-10.585	-11.317	-9.978	-10.183

Table S73: Correlation part of the interaction energies for different Polyene stack dimers in Fixed geometry for the aug-cc-pVQZ basis set in millihartree (not cp-corrected).

aQZ		CCSD(T) cor	LNO-CCSD(T) cor				DLPNO-CCSD(T) Tight	
System	D-Bonds	canonical	vvTight	vTight	Tight	Normal	TcutPNO=1e-6	TcutPNO=1e-7
c2_c2	2	-1.158	-1.164	-1.160	-1.196	-1.294	-1.209	-1.190
c4_c4	4	-2.552	-2.576	-2.585	-2.758	-2.869	-2.646	-2.654
c6_c6	6		-4.137	-4.167	-4.380	-4.552		-4.261
c8_c8	8		-5.730	-5.784	-6.072	-6.358		
c10_c10	10		-7.350	-7.432	-7.736	-8.350		
c12_c12	12		-9.083	-9.073	-9.524	-10.248		
c14_c14	14			-10.709	-11.159	-12.097		

Table S74: Correlation part of the interaction energies for different Polyene stack dimers in Fixed geometry for the cc-pV5Z basis set in millihartree (not cp-corrected).

5Z		CCSD(T) cor	LNO-CCSD(T) cor				DLPNO-CCSD(T) Tight	
System	D-Bonds	canonical	vvTight	vTight	Tight	Normal	TcutPNO=1e-6	TcutPNO=1e-7
c2_c2	2	-1.093	-1.100	-1.103	-1.140	-1.258	-1.118	-1.109
c4_c4	4	-2.417	-2.443	-2.444	-2.623	-2.769	-2.509	-2.524
c6_c6	6	-3.857	-3.916	-3.955	-4.187	-4.564		
c8_c8	8		-5.438	-5.502	-5.840	-6.391		

Table S75: Correlation part of the interaction energies for different Polyene stack dimers in Fixed geometry for the aug-cc-pV5Z basis set in millihartree (not cp-corrected).

a5Z		CCSD(T) cor	LNO-CCSD(T) cor				DLPNO-CCSD(T) Tight	
System	D-Bonds	canonical	vvTight	vTight	Tight	Normal	TcutPNO=1e-6	TcutPNO=1e-7
c2_c2	2	-1.138	-1.146	-1.140	-1.164	-1.232	-1.188	-1.186
c4_c4	4	-2.493	-2.523	-2.524	-2.665	-2.883	-2.602	-2.597
c6_c6	6		-4.018	-4.058	-4.270	-4.490		
c8_c8	8		-5.562	-5.621	-5.917	-6.398		

Table S76: Correlation part of the interaction energies for different Polyene stack dimers in Relaxed geometry for the cc-pVDZ basis set in millihartree (cp-corrected).

DZ		CCSD cor	LNO-CCSD cor				DLPNO-CCSD Tight	
System	D-Bonds	canonical	vvTight	vTight	Tight	Normal	TcutPNO=1e-6	TcutPNO=1e-7
c2_c2	2	-0.607	-0.618	-0.618	-0.569	-0.529	-0.645	-0.638
c4_c4	4	-2.417	-2.475	-2.460	-2.483	-2.267	-2.513	-2.541
c6_c6	6	-4.338	-4.448	-4.424	-4.464	-4.247	-4.569	-4.629
c8_c8	8	-6.435	-6.599	-6.576	-6.657	-6.453	-6.831	-6.884
c10_c10	10	-8.424	-8.675	-8.644	-8.751	-8.510	-8.973	-9.034
c12_c12	12	-10.303	-10.608	-10.581	-10.669	-10.388	-11.014	-11.081
c14_c14	14	-12.610	-12.995	-12.947	-13.086	-12.747	-13.380	-13.466

Table S77: Correlation part of the interaction energies for different Polyene stack dimers in Relaxed geometry for the aug-cc-pVDZ basis set in millihartree (cp-corrected).

aDZ		CCSD cor	LNO-CCSD cor				DLPNO-CCSD Tight	
System	D-Bonds	canonical	vvTight	vTight	Tight	Normal	TcutPNO=1e-6	TcutPNO=1e-7
c2_c2	2	-0.943	-0.962	-0.960	-0.935	-0.994	-0.971	-0.964
c4_c4	4	-3.433	-3.494	-3.478	-3.489	-3.299	-3.469	-3.452
c6_c6	6	-6.038	-6.171	-6.174	-6.117	-5.826	-6.137	-6.142
c8_c8	8		-9.086	-9.060	-8.947	-8.677	-9.018	-9.025
c10_c10	10		-11.822	-11.807	-11.670	-11.367	-11.867	-11.869
c12_c12	12		-14.450	-14.403	-14.249	-13.798	-14.538	-14.548
c14_c14	14		-17.577	-17.521	-17.367	-16.856		-17.737

Table S78: Correlation part of the interaction energies for different Polyene stack dimers in Relaxed geometry for the cc-pVTZ basis set in millihartree (cp-corrected).

TZ		CCSD cor	LNO-CCSD cor				DLPNO-CCSD Tight	
System	D-Bonds	canonical	vvTight	vTight	Tight	Normal	TcutPNO=1e-6	TcutPNO=1e-7
c2_c2	2	-0.797	-0.813	-0.809	-0.802	-0.830	-0.848	-0.858
c4_c4	4	-3.015	-3.104	-3.070	-3.140	-3.178	-3.161	-3.163
c6_c6	6	-5.362	-5.535	-5.509	-5.675	-5.593	-5.594	-5.602
c8_c8	8	-7.915	-8.199	-8.147	-10.110	-8.267	-8.204	-8.251
c10_c10	10	-10.338	-10.730	-10.654	-10.893	-10.960	-10.726	-10.794
c12_c12	12	-12.624	-13.095	-13.019	-13.337	-13.372	-13.156	-13.247
c14_c14	14	-15.417	-15.965	-15.905	-16.307	-16.285	-16.062	-16.166

Table S79: Correlation part of the interaction energies for different Polyene stack dimers in Relaxed geometry for the aug-cc-pVTZ basis set in millihartree (cp-corrected).

aTZ		CCSD cor	LNO-CCSD cor				DLPNO-CCSD Tight	
System	D-Bonds	canonical	vvTight	vTight	Tight	Normal	TcutPNO=1e-6	TcutPNO=1e-7
c2_c2	2	-0.940	-0.959	-0.956	-0.930	-1.039	-0.983	-0.969
c4_c4	4	-3.424	-3.504	-3.504	-3.457	-3.376	-3.538	-3.494
c6_c6	6	-6.023	-6.184	-6.187	-6.096	-5.837	-6.244	-6.193
c8_c8	8		-9.086	-9.060	-8.902	-8.565	-9.158	
c10_c10	10		-11.843	-11.789	-11.705	-11.300	-11.964	-11.928
c12_c12	12		-14.477	-14.393	-14.330	-13.869		
c14_c14	12		-17.637	-17.537	-17.439	-16.928	-17.890	-17.881

Table S80: Correlation part of the interaction energies for different Polyene stack dimers in Relaxed geometry for the cc-pVQZ basis set in millihartree (cp-corrected).

QZ		CCSD cor	LNO-CCSD cor				DLPNO-CCSD Tight	
System	D-Bonds	canonical	vvTight	vTight	Tight	Normal	TcutPNO=1e-6	TcutPNO=1e-7
c2_c2	2	-0.875	-0.890	-0.887	-0.895	-0.831	-0.897	-0.889
c4_c4	4	-3.251	-3.346	-3.328	-3.376	-3.230	-3.364	-3.335
c6_c6	6	-5.758	-5.950	-5.926	-5.985	-5.822	-5.991	-5.954
c8_c8	8	-8.482	-8.781	-8.729	-8.829	-8.558	-8.778	-8.763
c10_c10	10	-11.064	-11.477	-11.413	-11.530	-11.155	-11.523	-11.496
c12_c12	12	-13.500	-14.000	-13.926	-14.083	-13.696	-14.114	-14.071
c14_c14	14		-17.106	-16.988	-17.170	-16.782	-17.189	-17.135

Table S81: Correlation part of the interaction energies for different Polyene stack dimers in Relaxed geometry for the aug-cc-pVQZ basis set in millihartree (cp-corrected).

aQZ		CCSD cor	LNO-CCSD cor				DLPNO-CCSD Tight	
System	D-Bonds	canonical	vvTight	vTight	Tight	Normal	TcutPNO=1e-6	TcutPNO=1e-7
c2_c2	2	-0.939	-0.962	-0.959	-0.955	-0.897	-0.986	-0.973
c4_c4	4	-3.429	-3.523	-3.513	-3.456	-3.361	-3.551	-3.525
c6_c6	6		-6.215	-6.217	-6.178	-5.930	-6.248	
c8_c8	8		-9.136	-9.105	-9.047	-8.737	-9.180	-9.142
c10_c10	10			-11.863	-11.792	-11.405	-12.023	
c12_c12	12			-14.458	-14.381	-13.938		
c14_c14	14			-17.632	-17.547	-17.060		

Table S82: Correlation part of the interaction energies for different Polyene stack dimers in Relaxed geometry for the cc-pV5Z basis set in millihartree (cp-corrected).

5Z		CCSD cor	LNO-CCSD cor				DLPNO-CCSD Tight	
System	D-Bonds	canonical	vvTight	vTight	Tight	Normal	TcutPNO=1e-6	TcutPNO=1e-7
c2_c2	2	-0.912	-0.934	-0.930	-0.922	-0.856	-0.969	-0.950
c4_c4	4	-3.366	-3.465	-3.441	-3.454	-3.367	-3.526	-3.461
c6_c6	6		-6.119	-6.113	-6.138	-5.936		
c8_c8	8		-9.026	-9.000	-9.001	-8.768		

Table S83: Correlation part of the interaction energies for different Polyene stack dimers in Relaxed geometry for the aug-cc-pV5Z basis set in millihartree (cp-corrected).

a5Z		CCSD cor	LNO-CCSD cor				DLPNO-CCSD Tight	
System	D-Bonds	canonical	vvTight	vTight	Tight	Normal	TcutPNO=1e-6	TcutPNO=1e-7
c2_c2	2	-0.942	-0.968	-0.964	-0.958	-0.910	-0.992	-0.984
c4_c4	4	-3.435	-3.526	-3.517	-3.492	-3.368	-3.574	-3.540
c6_c6	6		-6.219	-6.221	-6.160	-5.914		
c8_c8	8		-9.135	-9.124	-9.056	-8.801		

Table S84: Correlation part of the interaction energies for different Polyene stack dimers in Fixed geometry for the cc-pVDZ basis set in millihartree (cp-corrected).

DZ		CCSD cor	LNO-CCSD cor				DLPNO-CCSD Tight	
System	D-Bonds	canonical	vvTight	vTight	Tight	Normal	TcutPNO=1e-6	TcutPNO=1e-7
c2_c2	2	-0.607	-0.618	-0.618	-0.569	-0.529	-0.645	-0.638
c4_c4	4	-1.367	-1.402	-1.383	-1.326	-1.327	-1.505	-1.504
c6_c6	6	-2.193	-2.260	-2.231	-2.153	-2.001	-2.470	-2.482
c8_c8	8	-3.042	-3.152	-3.110	-2.995	-1.960	-3.474	-3.501
c10_c10	10	-3.903	-4.059	-3.997	-3.879	-3.550	-4.486	-4.531
c12_c12	12	-4.769	-4.958	-4.882	-4.764	-4.374	-5.500	-5.544
c14_c14	14	-5.639	-5.863	-5.774	-5.627	-5.149	-6.557	-6.608

Table S85: Correlation part of the interaction energies for different Polyene stack dimers in Fixed geometry for the aug-cc-pVDZ basis set in millihartree (cp-corrected).

aDZ		CCSD cor	LNO-CCSD cor				DLPNO-CCSD Tight	
System	D-Bonds	canonical	vvTight	vTight	Tight	Normal	TcutPNO=1e-6	TcutPNO=1e-7
c2_c2	2	-0.943	-0.962	-0.960	-0.935	-0.994	-0.971	-0.964
c4_c4	4	-2.050	-2.104	-2.091	-2.064	-2.099	-2.195	-2.196
c6_c6	6	-3.235	-3.319	-3.299	-3.245	-3.088		
c8_c8	8		-4.587	-4.572	-4.440	-4.242		
c10_c10	10		-5.869	-5.827	-5.701	-5.303		
c12_c12	12		-7.120	-7.065	-6.844	-6.413		
c14_c14	14		-8.439	-8.344	-8.065	-7.628		

Table S86: Correlation part of the interaction energies for different Polyene stack dimers in Fixed geometry for the cc-pVTZ basis set in millihartree (cp-corrected).

TZ		CCSD cor	LNO-CCSD cor				DLPNO-CCSD Tight	
System	D-Bonds	canonical	vvTight	vTight	Tight	Normal	TcutPNO=1e-6	TcutPNO=1e-7
c2_c2	2	-0.797	-0.813	-0.809	-0.802	-0.830	-0.848	-0.858
c4_c4	4	-1.748	-1.813	-1.801	-1.845	-1.759	-1.914	-1.934
c6_c6	6	-2.779	-2.912	-2.901	-2.902	-2.739	-3.117	-3.153
c8_c8	8	-3.835	-4.024	-3.995	-4.038	-3.622	-4.335	-4.411
c10_c10	10	-4.905	-5.176	-5.141	-5.076	-4.753	-5.620	-5.699
c12_c12	12	-5.979	-6.318	-6.274	-6.244	-5.803	-6.880	-6.996
c14_c14	14	-7.057	-7.417	-7.374	-7.339	-6.884	-8.155	-8.293

Table S87: Correlation part of the interaction energies for different Polyene stack dimers in Fixed geometry for the aug-cc-pVTZ basis set in millihartree (cp-corrected).

aTZ		CCSD cor	LNO-CCSD cor				DLPNO-CCSD Tight	
System	D-Bonds	canonical	vvTight	vTight	Tight	Normal	TcutPNO=1e-6	TcutPNO=1e-7
c2_c2	2	-0.940	-0.959	-0.956	-0.930	-1.039	-0.983	-0.969
c4_c4	4	-2.019	-2.077	-2.069	-2.032	-2.077	-2.192	-2.166
c6_c6	6	-3.176	-3.286	-3.278	-3.166	-2.882		
c8_c8	8		-4.513	-4.486	-4.317	-3.989		
c10_c10	10		-5.752	-5.692	-5.526	-5.200		
c12_c12	12		-7.016	-6.945	-6.744	-6.323		
c14_c14	14		-8.285	-8.195	-7.958	-7.468		

Table S88: Correlation part of the interaction energies for different Polyene stack dimers in Fixed geometry for the cc-pVQZ basis set in millihartree (cp-corrected).

QZ		CCSD cor	LNO-CCSD cor				DLPNO-CCSD Tight	
System	D-Bonds	canonical	vvTight	vTight	Tight	Normal	TcutPNO=1e-6	TcutPNO=1e-7
c2_c2	2	-0.875	-0.890	-0.887	-0.895	-0.831	-0.897	-0.889
c4_c4	4	-1.899	-1.959	-1.949	-1.982	-1.918	-2.045	-2.038
c6_c6	6	-3.005	-3.124	-3.109	-3.142	-3.000	-3.364	-3.362
c8_c8	8	-4.137	-4.318	-4.289	-4.339	-4.085	-4.688	-4.696
c10_c10	10	-5.283	-5.526	-5.488	-5.491	-5.138	-6.044	-6.064
c12_c12	12	-6.434	-6.773	-6.718	-6.748	-5.908	-7.418	-7.448
c14_c14	14		-7.977	-7.924	-7.883	-7.278	-8.797	-8.832

Table S89: Correlation part of the interaction energies for different Polyene stack dimers in Fixed geometry for the aug-cc-pVQZ basis set in millihartree (cp-corrected).

aQZ		CCSD cor	LNO-CCSD cor				DLPNO-CCSD Tight	
System	D-Bonds	canonical	vvTight	vTight	Tight	Normal	TcutPNO=1e-6	TcutPNO=1e-7
c2_c2	2	-0.939	-0.962	-0.959	-0.955	-0.897	-0.986	-0.973
c4_c4	4	-2.008	-2.074	-2.062	-2.029	-1.916	-2.199	-2.168
c6_c6	6		-3.285	-3.269	-3.213	-2.983		-3.509
c8_c8	8		-4.511	-4.480	-4.373	-3.964		
c10_c10	10		-5.752	-5.703	-5.572	-5.233		
c12_c12	12		-7.025	-6.936	-6.778	-6.403		
c14_c14	14			-8.166	-7.993	-7.566		

Table S90: Correlation part of the interaction energies for different Polyene stack dimers in Fixed geometry for the cc-pV5Z basis set in millihartree (cp-corrected).

5Z		CCSD cor	LNO-CCSD cor				DLPNO-CCSD Tight	
System	D-Bonds	canonical	vvTight	vTight	Tight	Normal	TcutPNO=1e-6	TcutPNO=1e-7
c2_c2	2	-0.912	-0.934	-0.930	-0.922	-0.856	-0.969	-0.950
c4_c4	4	-1.962	-2.028	-2.011	-2.031	-1.878	-2.174	-2.132
c6_c6	6		-3.214	-3.204	-3.212	-2.994		
c8_c8	8		-4.428	-4.407	-4.421	-4.179		

Table S91: Correlation part of the interaction energies for different Polyene stack dimers in Fixed geometry for the aug-cc-pV5Z basis set in millihartree (cp-corrected).

a5Z		CCSD cor	LNO-CCSD cor				DLPNO-CCSD Tight	
System	D-Bonds	canonical	vvTight	vTight	Tight	Normal	TcutPNO=1e-6	TcutPNO=1e-7
c2_c2	2	-0.942	-0.968	-0.964	-0.958	-0.910	-0.992	-0.984
c4_c4	4	-2.008	-2.077	-2.062	-2.043	-1.893	-2.200	-2.177
c6_c6	6		-3.271	-3.261	-3.205	-3.057		
c8_c8	8		-4.498	-4.480	-4.389	-4.295		

Table S92: Correlation part of the interaction energies for different Polyene stack dimers in Relaxed geometry for the cc-pVDZ basis set in millihartree (not cp-corrected).

DZ		CCSD cor	LNO-CCSD cor				DLPNO-CCSD Tight	
System	D-Bonds	canonical	vvTight	vTight	Tight	Normal	TcutPNO=1e-6	TcutPNO=1e-7
c2_c2	2	-0.548	-0.552	-0.551	-0.501	-0.455	-0.574	-0.567
c4_c4	4	-2.408	-2.485	-2.468	-2.414	-2.127	-2.494	-2.488
c6_c6	6	-4.480	-4.604	-4.575	-4.483	-4.088	-4.692	-4.708
c8_c8	8	-6.781	-6.989	-6.953	-6.801	-6.345	-7.129	-7.132
c10_c10	10	-8.965	-9.231	-9.184	-9.042	-8.592	-9.432	-9.422
c12_c12	12	-11.007	-11.384	-11.315	-11.146	-10.563	-11.580	-11.579
c14_c14	14	-13.455	-13.933	-13.859	-13.645	-12.958	-14.046	-14.044

Table S93: Correlation part of the interaction energies for different Polyene stack dimers in Relaxed geometry for the aug-cc-pVDZ basis set in millihartree (not cp-corrected).

aDZ		CCSD cor	LNO-CCSD cor				DLPNO-CCSD Tight	
System	D-Bonds	canonical	vvTight	vTight	Tight	Normal	TcutPNO=1e-6	TcutPNO=1e-7
c2_c2	2	-1.232	-1.249	-1.246	-1.213	-1.163	-1.259	-1.250
c4_c4	4	-4.505	-4.549	-4.543	-4.526	-4.422	-4.533	-4.517
c6_c6	6	-8.007	-8.144	-8.143	-8.114	-7.840	-8.018	-8.042
c8_c8	8		-10.642	-10.627	-11.967	-11.717	-11.838	-11.879
c10_c10	10		-15.806	-15.783	-15.721	-15.272	-15.559	-15.567
c12_c12	12		-19.362	-19.300	-19.171	-18.813	-19.010	-19.015
c14_c14	14		-23.442	-23.412	-23.410	-22.827		-22.991

Table S94: Correlation part of the interaction energies for different Polyene stack dimers in Relaxed geometry for the cc-pVTZ basis set in millihartree (not cp-corrected).

TZ		CCSD cor	LNO-CCSD cor				DLPNO-CCSD Tight	
System	D-Bonds	canonical	vvTight	vTight	Tight	Normal	TcutPNO=1e-6	TcutPNO=1e-7
c2_c2	2	-0.767	-0.785	-0.781	-0.762	-0.753	-0.809	-0.821
c4_c4	4	-3.160	-3.246	-3.219	-3.211	-3.047	-3.268	-3.278
c6_c6	6	-5.715	-5.881	-5.849	-5.836	-5.571	-5.893	-5.884
c8_c8	8	-8.506	-8.773	-8.729	-8.698	-8.275	-8.716	-8.718
c10_c10	10	-11.154	-11.516	-11.437	-11.400	-10.898	-11.399	-11.415
c12_c12	12	-13.645	-14.073	-13.989	-13.959	-13.422	-13.981	-14.008
c14_c14	14	-16.645	-17.128	-17.053	-17.006	-16.383	-17.046	-17.085

Table S95: Correlation part of the interaction energies for different Polyene stack dimers in Relaxed geometry for the aug-cc-pVTZ basis set in millihartree (not cp-corrected).

aTZ		CCSD cor	LNO-CCSD cor				DLPNO-CCSD Tight	
System	D-Bonds	canonical	vvTight	vTight	Tight	Normal	TcutPNO=1e-6	TcutPNO=1e-7
c2_c2	2	-1.056	-1.075	-1.072	-1.059	-1.000	-1.111	-1.097
c4_c4	4	-3.962	-4.046	-4.047	-4.003	-3.693	-4.104	-4.042
c6_c6	6	-7.041	-7.212	-7.225	-7.144	-6.679	-7.245	-7.156
c8_c8	8		-10.642	-10.627	-10.519	-10.126	-10.659	
c10_c10	10		-13.884	-13.887	-13.754	-13.134	-13.846	-13.722
c12_c12	12		-16.961	-16.979	-16.863	-16.211	-16.897	
c14_c14	14		-20.662	-20.629	-20.471	-19.828	-20.518	-20.403

Table S96: Correlation part of the interaction energies for different Polyene stack dimers in Relaxed geometry for the cc-pVQZ basis set in millihartree (not cp-corrected).

QZ		CCSD cor	LNO-CCSD cor				DLPNO-CCSD Tight	
System	D-Bonds	canonical	vvTight	vTight	Tight	Normal	TcutPNO=1e-6	TcutPNO=1e-7
c2_c2	2	-0.869	-0.887	-0.882	-0.868	-0.803	-0.884	-0.876
c4_c4	4	-3.323	-3.422	-3.405	-3.383	-3.238	-3.417	-3.381
c6_c6	6	-5.921	-6.111	-6.080	-6.074	-5.827	-6.118	-6.064
c8_c8	8	-8.749	-9.046	-8.982	-8.957	-8.621	-8.978	-8.939
c10_c10	10	-11.431	-11.834	-11.761	-11.722	-11.289	-11.817	-11.741
c12_c12	12	-13.959	-14.440	-14.358	-14.338	-13.827	-14.476	-14.376
c14_c14	14		-17.584	-17.482	-17.439	-16.895	-17.602	-17.490

Table S97: Correlation part of the interaction energies for different Polyene stack dimers in Relaxed geometry for the aug-cc-pVQZ basis set in millihartree (not cp-corrected).

aQZ		CCSD cor	LNO-CCSD cor				DLPNO-CCSD Tight	
System	D-Bonds	canonical	vvTight	vTight	Tight	Normal	TcutPNO=1e-6	TcutPNO=1e-7
c2_c2	2	-0.980	-1.003	-1.000	-0.981	-0.922	-1.046	-1.017
c4_c4	4	-3.593	-3.684	-3.676	-3.644	-3.500	-3.766	-3.703
c6_c6	6		-6.524	-6.518	-6.484	-6.191	-6.620	
c8_c8	8		-9.593	-9.573	-9.536	-8.961	-9.688	-9.591
c10_c10	10			-12.479	-12.443	-12.024	-12.663	
c12_c12	12			-15.209	-15.156	-14.687		
c14_c14	14			-18.500	-18.407	-17.662		

Table S98: Correlation part of the interaction energies for different Polyene stack dimers in Relaxed geometry for the cc-pV5Z basis set in millihartree (not cp-corrected).

5Z		CCSD cor	LNO-CCSD cor				DLPNO-CCSD Tight	
System	D-Bonds	canonical	vvTight	vTight	Tight	Normal	TcutPNO=1e-6	TcutPNO=1e-7
c2_c2	2	-0.923	-0.945	-0.941	-0.926	-0.859	-0.964	-0.942
c4_c4	4	-3.420	-3.517	-3.494	-3.486	-3.377	-3.575	-3.521
c6_c6	6	-6.046	-6.221	-6.213	-6.189	-5.955		
c8_c8	8		-9.184	-9.153	-9.110	-8.816		

Table S99: Correlation part of the interaction energies for different Polyene stack dimers in Relaxed geometry for the aug-cc-pV5Z basis set in millihartree (not cp-corrected).

a5Z		CCSD cor	LNO-CCSD cor				DLPNO-CCSD Tight	
System	D-Bonds	canonical	vvTight	vTight	Tight	Normal	TcutPNO=1e-6	TcutPNO=1e-7
c2_c2	2	-0.961	-0.985	-0.982	-0.958	-0.910	-1.029	-1.012
c4_c4	4	-3.513	-3.602	-3.593	-3.567	-3.421	-3.691	-3.632
c6_c6	6	-6.187	-6.351	-6.362	-6.295	-5.996		
c8_c8	8		-9.335	-9.325	-9.264	-8.915		

Table S100: Correlation part of the interaction energies for different Polyene stack dimers in Fixed geometry for the cc-pVDZ basis set in millihartree (not cp-corrected).

DZ		CCSD cor	LNO-CCSD cor				DLPNO-CCSD Tight	
System	D-Bonds	canonical	vvTight	vTight	Tight	Normal	TcutPNO=1e-6	TcutPNO=1e-7
c2_c2	2	-0.548	-0.552	-0.551	-0.501	-0.455	-0.574	-0.567
c4_c4	4	-1.306	-1.355	-1.342	-1.240	-1.099	-1.430	-1.424
c6_c6	6	-2.136	-2.221	-2.204	-2.043	-1.756	-2.393	-2.387
c8_c8	8	-2.989	-3.105	-3.070	-2.851	-2.513	-3.389	-3.402
c10_c10	10	-3.856	-3.988	-3.944	-3.735	-3.391	-4.410	-4.414
c12_c12	12	-4.728	-4.922	-4.860	-4.620	-4.164	-5.436	-5.441
c14_c14	14	-5.603	-5.823	-5.770	-5.453	-4.954	-6.456	-6.469

Table S101: Correlation part of the interaction energies for different Polyene stack dimers in Fixed geometry for the aug-cc-pVDZ basis set in millihartree (not cp-corrected).

aDZ		CCSD cor	LNO-CCSD cor				DLPNO-CCSD Tight	
System	D-Bonds	canonical	vvTight	vTight	Tight	Normal	TcutPNO=1e-6	TcutPNO=1e-7
c2_c2	2	-1.232	-1.249	-1.246	-1.213	-1.163	-1.259	-1.250
c4_c4	4	-2.767	-2.803	-2.796	-2.763	-2.617	-2.889	-2.896
c6_c6	6	-4.431	-4.523	-4.512	-4.468	-4.156		
c8_c8	8		-6.340	-6.323	-6.241	-5.847		
c10_c10	10		-8.187	-8.145	-8.069	-7.533		
c12_c12	12		-10.015	-9.983	-9.737	-9.349		
c14_c14	14		-11.908	-11.827	-11.701	-11.147		

Table S102: Correlation part of the interaction energies for different Polyene stack dimers in Fixed geometry for the cc-pVTZ basis set in millihartree (not cp-corrected).

TZ		CCSD cor	LNO-CCSD cor				DLPNO-CCSD Tight	
System	D-Bonds	canonical	vvTight	vTight	Tight	Normal	TcutPNO=1e-6	TcutPNO=1e-7
c2_c2	2	-0.767	-0.785	-0.781	-0.762	-0.753	-0.809	-0.821
c4_c4	4	-1.761	-1.815	-1.803	-1.802	-1.652	-1.912	-1.930
c6_c6	6	-2.839	-2.940	-2.921	-2.873	-2.656	-3.130	-3.166
c8_c8	8	-3.944	-4.099	-4.068	-3.981	-3.615	-4.389	-4.445
c10_c10	10	-5.063	-5.270	-5.225	-5.104	-4.674	-5.668	-5.745
c12_c12	12	-6.187	-6.438	-6.382	-6.257	-5.717	-6.936	-7.049
c14_c14	14	-7.315	-7.600	-7.541	-7.377	-6.808	-8.221	-8.365

Table S103: Correlation part of the interaction energies for different Polyene stack dimers in Fixed geometry for the aug-cc-pVTZ basis set in millihartree (not cp-corrected).

aTZ		CCSD cor	LNO-CCSD cor				DLPNO-CCSD Tight	
System	D-Bonds	canonical	vvTight	vTight	Tight	Normal	TcutPNO=1e-6	TcutPNO=1e-7
c2_c2	2	-1.056	-1.075	-1.072	-1.059	-1.000	-1.111	-1.097
c4_c4	4	-2.392	-2.453	-2.448	-2.407	-2.224	-2.584	-2.544
c6_c6	6	-3.816	-3.933	-3.934	-3.780	-3.455		
c8_c8	8		-5.433	-5.414	-5.289	-4.725		
c10_c10	10		-6.935	-6.922	-6.772	-6.278		
c12_c12	12		-8.484	-8.461	-8.267	-7.699		
c14_c14	14		-10.025	-9.974	-9.709	-9.117		

Table S104: Correlation part of the interaction energies for different Polyene stack dimers in Fixed geometry for the cc-pVQZ basis set in millihartree (not cp-corrected).

QZ		CCSD cor	LNO-CCSD cor				DLPNO-CCSD Tight	
System	D-Bonds	canonical	vvTight	vTight	Tight	Normal	TcutPNO=1e-6	TcutPNO=1e-7
c2_c2	2	-0.869	-0.887	-0.882	-0.868	-0.803	-0.884	-0.876
c4_c4	4	-1.926	-1.987	-1.977	-1.946	-1.878	-2.058	-2.041
c6_c6	6	-3.069	-3.185	-3.168	-3.107	-2.964	-3.353	-3.356
c8_c8	8	-4.240	-4.414	-4.376	-4.280	-4.046	-4.707	-4.701
c10_c10	10	-5.424	-5.658	-5.610	-5.493	-5.132	-6.081	-6.083
c12_c12	12	-6.613	-6.908	-6.844	-6.727	-6.287	-7.471	-7.490
c14_c14	14		-8.133	-8.078	-7.933	-7.399	-8.866	-8.887

Table S105: Correlation part of the interaction energies for different Polyene stack dimers in Fixed geometry for the aug-cc-pVQZ basis set in millihartree (not cp-corrected).

aQZ		CCSD cor	LNO-CCSD cor				DLPNO-CCSD Tight	
System	D-Bonds	canonical	vvTight	vTight	Tight	Normal	TcutPNO=1e-6	TcutPNO=1e-7
c2_c2	2	-0.980	-1.003	-1.000	-0.981	-0.922	-1.046	-1.017
c4_c4	4	-2.116	-2.180	-2.171	-2.161	-2.024	-2.317	-2.284
c6_c6	6		-3.472	-3.450	-3.399	-3.114		-3.691
c8_c8	8		-4.774	-4.744	-4.670	-4.262		
c10_c10	10		-6.094	-6.057	-5.926	-5.575		
c12_c12	12		-7.495	-7.368	-7.249	-6.843		
c14_c14	14			-8.670	-8.493	-8.063		

Table S106: Correlation part of the interaction energies for different Polyene stack dimers in Fixed geometry for the cc-pV5Z basis set in millihartree (not cp-corrected).

5Z		CCSD cor	LNO-CCSD cor				DLPNO-CCSD Tight	
System	D-Bonds	canonical	vvTight	vTight	Tight	Normal	TcutPNO=1e-6	TcutPNO=1e-7
c2_c2	2	-0.923	-0.945	-0.941	-0.926	-0.859	-0.964	-0.942
c4_c4	4	-1.996	-2.060	-2.044	-2.044	-1.895	-2.194	-2.167
c6_c6	6	-3.153	-3.269	-3.257	-3.226	-3.009		
c8_c8	8		-4.506	-4.484	-4.434	-4.189		

Table S107: Correlation part of the interaction energies for different Polyene stack dimers in Fixed geometry for the aug-cc-pV5Z basis set in millihartree (not cp-corrected).

a5Z		CCSD cor	LNO-CCSD cor				DLPNO-CCSD Tight	
System	D-Bonds	canonical	vvTight	vTight	Tight	Normal	TcutPNO=1e-6	TcutPNO=1e-7
c2_c2	2	-0.961	-0.985	-0.982	-0.958	-0.910	-1.029	-1.012
c4_c4	4	-2.060	-2.127	-2.113	-2.092	-1.936	-2.276	-2.232
c6_c6	6	-3.247	-3.354	-3.346	-3.288	-3.063		
c8_c8	8		-4.617	-4.592	-4.512	-4.251		

Table S108: Correlation part of the interaction energies for different Polyene stack dimers in Relaxed geometry for the cc-pVDZ basis set in millihartree (cp-corrected).

DZ		PNO-LCCSD		PNO-LCCSD(T)	
System	D-Bonds	Tight	vTight	Tight	vTight
c2_c2	2	-0.620	-0.612	-0.687	-0.678
c4_c4	4	-2.371	-2.397	-2.693	-2.731
c6_c6	6	-4.288	-4.343	-4.911	-4.993
c8_c8	8	-6.356	-6.415	-7.311	-7.410
c10_c10	10	-8.423	-8.504	-9.702	-9.841
c12_c12	12	-10.280	-10.379	-11.864	-12.032
c14_c14	14	-12.477	-12.586	-14.425	-14.621

Table S109: Correlation part of the interaction energies for different Polyene stack dimers in Relaxed geometry for the aug-cc-pVDZ basis set in millihartree (cp-corrected).

aDZ		PNO-LCCSD		PNO-LCCSD(T)	
System	D-Bonds	Tight	vTight	Tight	vTight
c2_c2	2	-0.942	-0.939	-1.077	-1.076
c4_c4	4	-3.366	-3.388	-3.932	-3.969
c6_c6	6	-5.910	-5.954	-6.960	-7.033
c8_c8	8	-8.652	-8.725	-10.225	-10.348
c10_c10	10	-11.267	-11.374	-13.344	-13.521
c12_c12	12	-13.749	-13.887	-16.303	-16.534
c14_c14	14	-16.717	-16.888	-19.842	-20.127

Table S110: Correlation part of the interaction energies for different Polyene stack dimers in Relaxed geometry for the cc-pVTZ basis set in millihartree (cp-corrected).

TZ		PNO-LCCSD		PNO-LCCSD(T)	
System	D-Bonds	Tight	vTight	Tight	vTight
c2_c2	2	-0.797	-0.801	-0.916	-0.922
c4_c4	4	-2.999	-3.008	-3.519	-3.543
c6_c6	6	-5.331	-5.347	-6.305	-6.352
c8_c8	8	-7.847	-7.882	-9.308	-9.396
c10_c10	10	-10.250	-10.304	-12.176	-12.305
c12_c12	12	-12.510	-12.584	-14.878	-15.047
c14_c14	14	-15.275	-15.369	-18.182	-18.393

Table S111: Correlation part of the interaction energies for different Polyene stack dimers in Relaxed geometry for the cc-pVDZ basis set in millihartree (not cp-corrected).

DZ		PNO-LCCSD		PNO-LCCSD(T)	
System	D-Bonds	Tight	vTight	Tight	vTight
c2_c2	2	-0.530	-0.550	-0.600	-0.619
c4_c4	4	-2.328	-2.346	-2.691	-2.721
c6_c6	6	-4.353	-4.396	-5.059	-5.129
c8_c8	8	-6.621	-6.699	-7.718	-7.839
c10_c10	10	-8.773	-8.858	-10.231	-10.375
c12_c12	12	-10.687	-10.792	-12.479	-12.657
c14_c14	14	-12.880	-13.015	-15.048	-15.276

Table S112: Correlation part of the interaction energies for different Polyene stack dimers in Relaxed geometry for the aug-cc-pVDZ basis set in millihartree (not cp-corrected).

aDZ		PNO-LCCSD		PNO-LCCSD(T)	
System	D-Bonds	Tight	vTight	Tight	vTight
c2_c2	2	-1.147	-1.139	-1.306	-1.299
c4_c4	4	-3.928	-3.947	-4.572	-4.608
c6_c6	6	-6.726	-6.769	-7.903	-7.977
c8_c8	8	-9.808	-9.871	-11.568	-11.680
c10_c10	10	-12.724	-12.819	-15.038	-15.203
c12_c12	12	-15.475	-15.607	-18.309	-18.534
c14_c14	14	-18.700	-18.864	-22.128	-22.405

Table S113: Correlation part of the interaction energies for different Polyene stack dimers in Relaxed geometry for the cc-pVTZ basis set in millihartree (not cp-corrected).

TZ		PNO-LCCSD		PNO-LCCSD(T)	
System	D-Bonds	Tight	vTight	Tight	vTight
c2_c2	2	-0.746	-0.754	-0.872	-0.882
c4_c4	4	-3.078	-3.090	-3.642	-3.671
c6_c6	6	-5.478	-5.495	-6.512	-6.562
c8_c8	8	-8.064	-8.099	-9.600	-9.689
c10_c10	10	-10.517	-10.574	-12.533	-12.664
c12_c12	12	-12.836	-12.915	-15.310	-15.484
c14_c14	14	-15.608	-15.710	-18.599	-18.818

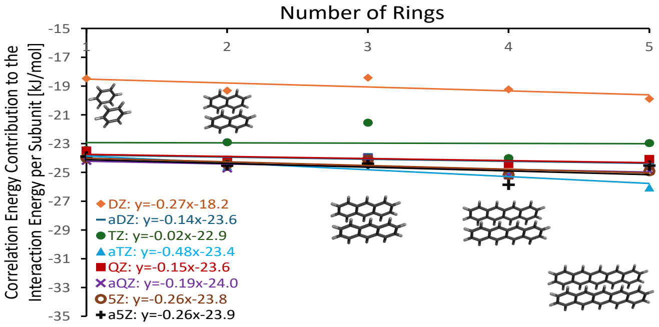

(a) Counterpoise-corrected energies for the acene series.

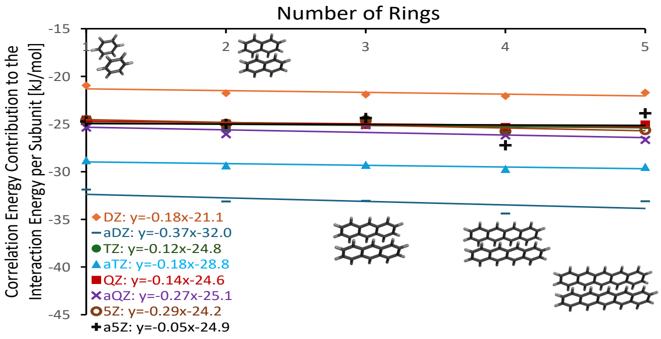

(b) Counterpoise-uncorrected energies for the acene series.

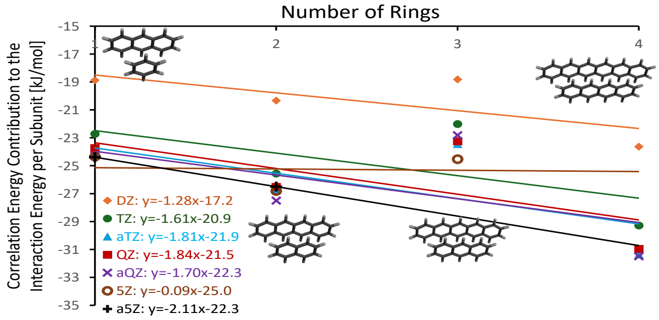

(c) Counterpoise-corrected energies for the acene-(acene-2) series.

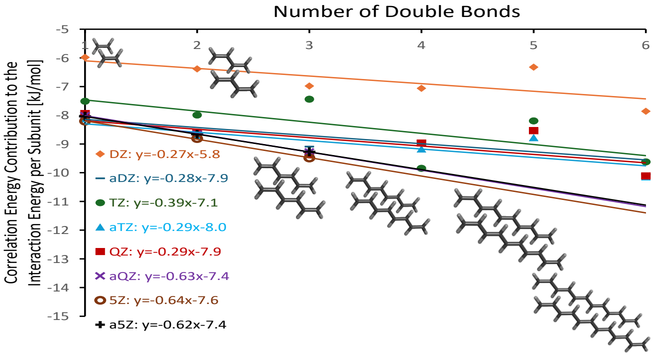

(d) Counterpoise-corrected energies for the ethylene stack series (relaxed geometry).

Figure S2: Differences in Correlation energies in kJ/mol vs. number of rings (acenes), number of atoms (acenes with acenes-2), or double-bonds (ethylene stacks) using the cc-pVnZ (X=D-5) and aug-cc-pVmZ (m=D-5) basis sets.

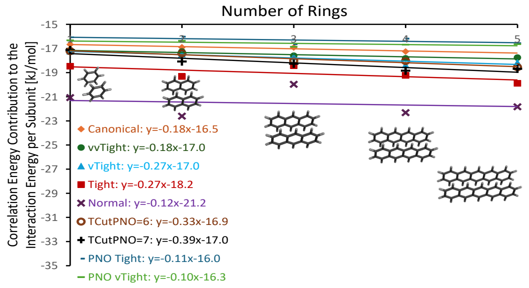

(a) Counterpoise-corrected DZ energies for the acene series.

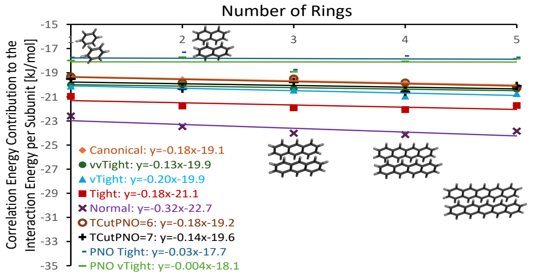

(b) Counterpoise-uncorrected DZ energies for the acene series.

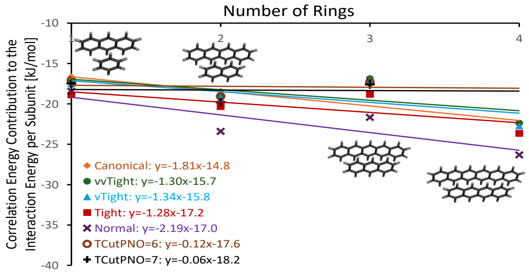

(c) Counterpoise-corrected DZ energies for the acene-acene-2 series.

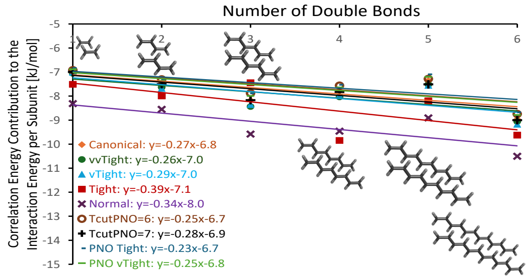

(d) Counterpoise corrected TZ Energies for the ethylene stack series (relaxed geometry).

Figure S3: Differences in Correlation energies (kJ/mol) derived from various coupled cluster methods in as a function of system size: per number of rings (acenes), per number of atoms (acenes with acenes-2), or per number of double bonds (ethylene stacks).

Table S114: Slopes (in kJ/mol per segment) and Intercepts (in kJ/mol) for Figure 1.

Subfigure		basis	Slope	Intercept
a	cp-corrected	DZ	-19.03	2.2
		aDZ	-24.09	2.5
		TZ	-22.89	2.1
		aTZ	-24.77	3.2
		QZ	-24.08	2.4
		aQZ	-24.60	2.6
		5Z	-24.52	2.3
		a5Z	-24.70	2.6
b	cp-uncorrected	DZ	-21.75	4.3
		aDZ	-33.22	5.6
		TZ	-25.16	3.4
		aTZ	-29.36	4.2
		QZ	-25.02	2.7
		aQZ	-25.84	2.6
		5Z	-25.11	2.7
		a5Z	-25.16	2.5
c	cp-corrected	DZ	-20.25	-11.9
		TZ	-24.68	-15.8
		aTZ	-26.18	-17.6
		QZ	-25.87	-16.8
		aQZ	-26.25	-18.2
		5Z	-25.39	-19.8
		a5Z	-25.45	-19.9
d	cp-corrected	DZ	-6.76	5.5
		aDZ	-8.83	6.5
		TZ	-8.43	6.8
		aTZ	-9.03	6.8
		QZ	-8.93	6.8
		aQZ	-8.65	6.0
		5Z	-8.83	6.2
		a5Z	-8.66	6.0

Table S115: Slopes (in kJ/mol per segment) and Intercepts (in kJ/mol) for Figure S1.

Subfigure		basis	Slope	Intercept
a	cp-corrected	DZ	-0.27	-18.2
		aDZ	-0.14	-23.6
		TZ	-0.02	-22.9
		aTZ	-0.48	-23.4
		QZ	-0.15	-23.6
		aQZ	-0.19	-24.0
		5Z	-0.26	-23.8
		a5Z	-0.26	-23.9
b	cp-corrected	DZ	-0.18	-21.1
		aDZ	-0.37	-32.0
		TZ	-0.12	-24.8
		aTZ	-0.18	-28.8
		QZ	-0.14	-24.6
		aQZ	-0.27	-25.1
		5Z	-0.29	-24.2
		a5Z	-0.05	-24.9
c	cp-corrected	DZ	-1.28	-17.2
		TZ	-1.61	-20.9
		aTZ	-1.81	-21.9
		QZ	-1.84	-21.5
		aQZ	-1.70	-22.3
		5Z	-0.09	-25.0
		a5Z	-2.11	-22.3
d	cp-corrected	DZ	-0.27	-5.8
		aDZ	-0.28	-7.9
		TZ	-0.39	-7.1
		aTZ	-0.29	-8.0
		QZ	-0.29	-7.9
		aQZ	-0.63	-7.4
		5Z	-0.64	-7.6
		a5Z	-0.62	-7.4

Table S116: Slopes (in kJ/mol per segment) and Intercepts (in kJ/mol) for Figure 2.

Subfigure		CCSD(T) method	Slope	Intercept
a	DZ cp-corrected	Canonical	-16.93	1.8
		LNO vvTight	-17.53	2.2
		LNO vTight	-17.79	2.5
		LNO Tight	-19.03	2.2
		LNO Normal	-21.53	2.9
		DLPNO Tcut=1e-6	-17.85	3.4
		DLPNO Tcut=1e-7	-18.25	3.5
		PNO Tight	-16.30	1.7
		PNO vTight	-16.60	1.9
b	DZ cp-uncorrected	Canonical	-19.58	3.1
		LNO vvTight	-20.25	3.4
		LNO vTight	-20.50	3.6
		LNO Tight	-21.75	4.3
		LNO Normal	-23.69	5.0
		DLPNO Tcut=1e-6	-19.73	3.8
		DLPNO Tcut=1e-7	-20.10	3.8
		PNO Tight	-17.87	2.1
		PNO vTight	-18.16	2.1
c	aTZ cp-corrected	Canonical	-22.67	1.2
		LNO vvTight	-23.50	1.9
		LNO vTight	-22.05	-1.6
		LNO Tight	-24.77	3.2
		LNO Normal	-26.95	4.1
		DLPNO Tcut=1e-6	-23.41	2.8
		DLPNO Tcut=1e-7	-22.96	1.6
d	DZ cp-corrected	Canonical	-17.54	-12.9
		LNO vvTight	-18.70	-11.6
		LNO vTight	-18.95	-11.6
		LNO Tight	-20.25	-11.9
		LNO Normal	-22.47	-14.0
		DLPNO Tcut=1e-6	-17.94	-12.3
		DLPNO Tcut=1e-7	-18.44	-12.6
e	TZ cp-corrected	Canonical	-7.77	6.0
		LNO vvTight	-7.96	6.2
		LNO vTight	-7.95	6.2
		LNO Tight	-8.43	6.8
		LNO Normal	-9.21	7.0
		DLPNO Tcut=1e-6	-7.59	5.6
		DLPNO Tcut=1e-7	-7.82	5.9
		PNO Tight	-7.54	5.7
		PNO vTight	-7.63	5.8

Table S117: Slopes (in kJ/mol per segment) and Intercepts (in kJ/mol) for Figure S2.

Subfigure		CCSD(T) method	Slope	Intercept
a	DZ cp-corrected	Canonical	-0.18	-16.5
		LNO vvTight	-0.18	-17.0
		LNO vTight	-0.27	-17.0
		LNO Tight	-0.27	-18.2
		LNO Normal	-0.12	-21.2
		DLPNO Tcut=1e-6	-0.33	-16.9
		DLPNO Tcut=1e-7	-0.39	-17.0
		PNO Tight	-0.11	-16.0
		PNO vTight	-0.10	-16.3
b	DZ cp-uncorrected	Canonical	-0.18	-19.1
		LNO vvTight	-0.13	-19.9
		LNO vTight	-0.20	-19.9
		LNO Tight	-0.18	-21.1
		LNO Normal	-0.32	-22.7
		DLPNO Tcut=1e-6	-0.18	-19.2
		DLPNO Tcut=1e-7	-0.14	-19.6
		PNO Tight	-0.03	-17.7
		PNO vTight	-0.004	-18.1
c	DZ cp-corrected	Canonical	-1.81	-14.8
		LNO vvTight	-1.30	-15.7
		LNO vTight	-1.34	-15.8
		LNO Tight	-1.28	-17.2
		LNO Normal	-2.19	-17.0
		DLPNO Tcut=1e-6	-0.12	-17.6
		DLPNO Tcut=1e-7	-0.06	-18.2
d	TZ cp-corrected	Canonical	-0.27	-6.8
		LNO vvTight	-0.26	-7.0
		LNO vTight	-0.29	-7.0
		LNO Tight	-0.39	-7.1
		LNO Normal	-0.34	-8.0
		DLPNO Tcut=1e-6	-0.25	-6.7
		DLPNO Tcut=1e-7	-0.28	-6.9
		PNO Tight	-0.23	-6.7
		PNO vTight	-0.25	-6.8

Raw Hartree-Fock, RPA and DFT-SAPT Data: Basis Set Effects

All raw Hartree-Fock, RPA (which uses the PBE exchange functional), as well as DFT-SAPT exchange part are reported in this section. Concerning DFT-SAPT, we are using the PBE0-AC

functional with electrostatic+exchange+induction+dHF contributions, whereas the asymptotic correction for the PBE0 functional have been determined by PBE0 ionization potentials and orbital energies of the individual monomers.

Table S118: Hartree-Fock, RPA, DFT-SAPT(electrostatic+exchange+induction) part of the interaction energies in kJ/mol, cp-corrected cc-pVDZ basis set for the acene series.

DZ		method		
System	Atoms	HF	DFT-SAPT ele	RPA exch
c_c	12	15.79	13.01	15.44
c2_c2	36	28.68	24.90	28.05
c3_c1	36	66.56	23.34	25.11
c3_c3	48	40.79	36.34	39.74
c4_c2	48	36.10	33.71	35.51
cc_c	48	30.45	30.28	30.28
c5_c1	48	25.20	23.50	24.90
c4_c4	60	52.43	47.47	50.95
c5_c3	60	49.07	46.44	48.14
c6_c2	60	36.54	34.57	35.88
c7_c1	60	25.14	23.63	24.81
cc_cc	72	78.21	66.88	69.87
c5_c5	72	64.06	58.72	62.19
c6_c4	72	59.51	56.59	58.20
c7_c3	72	48.70	46.55	47.70
c8_c2	72	36.39	34.67	35.69
c9_c1	72	25.11	23.79	24.80
c6_c6	84	75.66	69.89	73.28
c7_c5	84	75.60	72.74	73.90
c8_c4	84	61.69	59.37	60.32
ccc_c	84	29.07		
ccc_cc	108	92.74		
ccc_ccc	144	160.24		

Table S119: Hartree-Fock, RPA, DFT-SAPT(electrostatic+exchange+induction) part of the interaction energies in kJ/mol, cp-corrected cc-pVTZ basis set for the acene series.

TZ		method		
System	Atoms	HF	DFT-SAPT ele	RPA exch
c_c	12	15.40	12.43	14.95
c2_c2	36	27.77	24.06	27.69
c3_c1	36	24.67	22.65	25.00
c3_c3	48	39.90	35.33	39.69
c4_c2	48	35.39	32.85	35.66
cc_c	48	30.30	29.75	30.93
c5_c1	48	24.77	22.94	25.07
c4_c4	60	51.57	46.31	51.20
c5_c3	60	48.43	45.44	48.67
c6_c2	60	36.17	33.89	36.41
c7_c1	60	25.14	23.12	25.08
cc_cc	72	72.88		72.06
c5_c5	72	63.24	57.39	23.90
c6_c4	72	22.40	21.09	58.91
c7_c3	72	48.37	45.74	48.58
c8_c2	72	36.11	34.05	36.33
c9_c1	72	24.82		
c6_c6	84	74.86	74.14	68.46
c7_c5	84	75.20	75.29	71.49
c8_c4	84	61.46	58.43	
ccc_c	84	29.97		
ccc_cc	108	92.74		
ccc_ccc	144	164.93		

Table S120: Hartree-Fock part of the interaction energies in kJ/mol, cp-corrected aug-cc-pVTZ basis set for the acene series.

aTZ		method
System	Atoms	HF
c_c	12	14.70
c2_c2	36	27.58
c3_c1	36	24.41
c3_c3	48	39.76
c4_c2	48	35.14
cc_c	48	30.04
c5_c1	48	24.57
c4_c4	60	51.46
c5_c3	60	48.21
c6_c2	60	35.97
c7_c1	60	24.61
cc_cc	72	72.71
c5_c5	72	63.21
c6_c4	72	58.59
c7_c3	72	48.18
c8_c2	72	35.92
c9_c1	72	24.63
c6_c6	84	74.79
c7_c5	84	75.01
c8_c4	84	61.27
ccc_c	84	29.92
ccc_cc	108	95.39

Table S121: Hartree-Fock, RPA, DFT-SAPT(electrostatic+exchange+induction) part of the interaction energies in kJ/mol, cp-corrected cc-pVQZ basis set for the acene series.

QZ		method		
System	Atoms	HF	DFT-SAPT ele	RPA exch
c_c	12	14.82	12.33	14.92
c2_c2	36	27.70	23.94	27.78
c3_c1	36	24.55	22.47	25.01
c3_c3	48	39.87	35.19	39.88
c4_c2	48	35.29	32.65	35.75
cc_c	48	30.17		
c5_c1	48	24.69	22.83	25.14
c4_c4	60	51.57	46.10	51.48
c5_c3	60	48.36	45.20	48.86
c6_c2	60	36.11	33.73	36.57
c7_c1	60	24.74	23.02	25.16
cc_cc	72	72.80		
c5_c5	72	63.26	57.12	74.59
c6_c4	72	58.75	55.08	59.18
c7_c3	72	48.33	45.52	48.81
c8_c2	72	36.07		
c9_c1	72	24.75		
c6_c6	84	74.92		74.59
c7_c5	84	75.17		
c8_c4	84	61.43		
ccc_c	84	29.99		
ccc_cc	108	95.53		

Table S122: Hartree-Fock part of the interaction energies in kJ/mol, cp-corrected aug-cc-pVQZ basis set for the acene series.

aQZ		method
System	Atoms	HF
c_c	12	14.67
c2_c2	36	27.54
c3_c1	36	24.39
c3_c3	48	39.70
c4_c2	48	35.10
cc_c	48	30.03
c5_c1	48	24.55
c4_c4	60	51.40
c5_c3	60	48.17
c6_c2	60	35.94
c7_c1	60	24.60
cc_cc	72	72.64
c5_c5	72	63.13
c6_c4	72	58.54
c7_c3	72	48.14
c8_c2	72	35.92
c9_c1	72	24.61
c6_c6	84	74.71
c7_c5	84	74.94
c8_c4	84	61.22
ccc_c	84	29.91
ccc_cc	108	95.35

Table S123: Hartree-Fock part of the interaction energies in kJ/mol, cp-corrected cc-pV5Z basis set for the acene series.

5Z		method
System	Atoms	HF
c_c	12	14.69
c2_c2	36	27.56
c3_c1	36	24.40
c3_c3	48	39.72
c4_c2	48	35.11
cc_c	48	30.03
c5_c1	48	24.56
c4_c4	60	51.41
c5_c3	60	48.17
c6_c2	60	35.95
c7_c1	60	24.60
cc_cc	72	72.64
c5_c5	72	63.14
c6_c4	72	58.20
c7_c3	72	48.15
c8_c2	72	35.92
c9_c1	72	24.61
c6_c6	84	74.71
c7_c5	84	74.94
c8_c4	84	61.22
ccc_c	84	29.91

Table S124: Hartree-Fock part of the interaction energies in kJ/mol, cp-corrected aug-cc-pV5Z basis set for the acene series.

a5Z		method
System	Atoms	HF
c_c	12	14.66
c2_c2	36	27.54
c3_c1	36	24.39
c3_c3	48	39.70
c4_c2	48	35.10
cc_c	48	30.03
c5_c1	48	24.55
c4_c4	60	51.39
c5_c3	60	48.16
c6_c2	60	35.93
c7_c1	60	24.60
cc_cc	72	72.63
c5_c5	72	63.12
c6_c4	72	58.42
c7_c3	72	48.13
c8_c2	72	35.91
c9_c1	72	24.61
c6_c6	84	74.70
c7_c5	84	74.93
c8_c4	84	61.21
ccc_c	84	29.83

Table S125: Hartree-Fock, RPA part of the interaction energies in kJ/mol, cc-pVDZ basis set for the acene series.

DZ		method	
System	Atoms	HF	RPA exch
c_c	12	11.56	11.56
c2_c2	36	22.31	22.06
c3_c1	36	19.08	19.31
c3_c3	48	32.28	31.77
c4_c2	48	27.67	27.71
cc_c	48	22.99	
c5_c1	48	19.03	19.22
c4_c4	60	41.80	41.03
c5_c3	60	38.25	38.16
c6_c2	60	28.13	28.15
c7_c1	60	18.93	19.12
cc_cc	72	58.94	
c5_c5	72	51.40	50.32
c6_c4	72	46.61	46.30
c7_c3	72	37.98	37.87
c8_c2	72	27.88	
c9_c1	72	18.86	
c6_c6	84	60.78	59.48
c7_c5	84	59.89	59.47
c8_c4	84	48.59	48.33
ccc_c	84	21.89	
ccc_cc	108	71.28	
ccc_ccc	144	131.36	

Table S126: Hartree-Fock, RPA part of the interaction energies in kJ/mol, cc-pVTZ basis set for the acene series.

TZ		method	
System	Atoms	HF	RPA exch
c_c	12	13.39	13.55
c2_c2	36	25.40	25.61
c3_c1	36	22.22	22.88
c3_c3	48	36.79	36.99
c4_c2	48	32.18	32.91
cc_c	48	27.65	
c5_c1	48	22.44	23.10
c4_c4	60	47.71	47.91
c5_c3	60	44.41	45.26
c6_c2	60	33.08	33.83
c7_c1	60	22.44	23.09
cc_cc	72	67.85	
c5_c5	72	58.53	58.86
c6_c4	72	54.05	54.91
c7_c3	72	44.51	45.37
c8_c2	72	33.00	33.73
c9_c1	72	22.44	
c6_c6	84	69.50	69.65
c7_c5	84	69.47	70.52
c8_c4	84	56.79	
ccc_c	84	27.35	
ccc_cc	108	88.52	
ccc_ccc	144	154.49	

Table S127: Hartree-Fock part of the interaction energies in kJ/mol, aug-cc-pVTZ basis set for the acene series.

aTZ		method
System	Atoms	HF
c_c	12	13.93
c2_c2	36	25.94
c3_c1	36	22.89
c3_c3	48	37.36
c4_c2	48	32.83
cc_c	48	27.85
c5_c1	48	22.93
c4_c4	60	48.29
c5_c3	60	45.12
c6_c2	60	33.50
c7_c1	60	22.96
cc_cc	72	68.18
c5_c5	72	59.30
c6_c4	72	54.77
c7_c3	72	44.93
c8_c2	72	33.45
c9_c1	72	22.97
c6_c6	84	70.15
c7_c5	84	70.35
c8_c4	84	57.23
ccc_c	84	27.30
ccc_cc	108	88.65

Table S128: Hartree-Fock, RPA part of the interaction energies in kJ/mol, cc-pVQZ basis set for the acene series.

QZ		method	
System	Atoms	HF	RPA exch
c_c	12	14.17	14.45
c2_c2	36	26.74	27.21
c3_c1	36	23.51	24.41
c3_c3	48	38.63	39.27
c4_c2	48	33.95	35.08
cc_c	48	29.07	
c5_c1	48	23.73	24.67
c4_c4	60	50.05	50.84
c5_c3	60	46.71	48.12
c6_c2	60	34.85	36.04
c7_c1	60	23.75	24.70
cc_cc	72	70.87	
c5_c5	72	61.56	62.44
c6_c4	72	56.82	58.41
c7_c3	72	46.78	48.25
c8_c2	72	34.80	
c9_c1	72	23.76	
c6_c6	84	72.81	
c7_c5	84	72.87	
c8_c4	84	59.56	
ccc_c	84	29.32	
ccc_cc	108	92.69	

Table S129: Hartree-Fock part of the interaction energies in kJ/mol, aug-cc-pVQZ basis set for the acene series.

aQZ		method
System	Atoms	HF
c_c	12	14.46
c2_c2	36	27.16
c3_c1	36	24.02
c3_c3	48	39.17
c4_c2	48	34.58
cc_c	48	29.51
c5_c1	48	24.15
c4_c4	60	50.73
c5_c3	60	47.49
c6_c2	60	35.36
c7_c1	60	24.18
cc_cc	72	71.72
c5_c5	72	62.31
c6_c4	72	56.82
c7_c3	72	47.42
c8_c2	72	35.34
c9_c1	72	24.20
c6_c6	84	73.75
c7_c5	84	73.96
c8_c4	84	60.35
ccc_c	84	29.32
ccc_cc	108	93.94

Table S130: Hartree-Fock part of the interaction energies in kJ/mol, cc-pV5Z basis set for the acene series.

5Z		method
System	Atoms	HF
c_c	12	14.58
c2_c2	36	27.38
c3_c1	36	24.21
c3_c3	48	39.48
c4_c2	48	34.87
cc_c	48	29.84
c5_c1	48	24.39
c4_c4	60	51.13
c5_c3	60	47.88
c6_c2	60	35.73
c7_c1	60	24.44
cc_cc	72	72.30
c5_c5	72	62.82
c6_c4	72	58.20
c7_c3	72	47.88
c8_c2	72	35.70
c9_c1	72	24.45
c6_c6	84	74.34
c7_c5	84	74.53
c8_c4	84	60.90
ccc_c	84	29.73

Table S131: Hartree-Fock part of the interaction energies in kJ/mol, aug-cc-pV5Z basis set for the acene series.

a5Z		
System	Atoms	HF
c_c	12	14.63
c2_c2	36	27.49
c3_c1	36	24.34
c3_c3	48	39.63
c4_c2	48	35.04
cc_c	48	29.96
c5_c1	48	24.50
c4_c4	60	51.31
c5_c3	60	48.07
c6_c2	60	35.86
c7_c1	60	24.44
cc_cc	72	72.51
c5_c5	72	63.02
c6_c4	72	58.42
c7_c3	72	48.04
c8_c2	72	35.84
c9_c1	72	24.56
c6_c6	84	74.57
c7_c5	84	74.80
c8_c4	84	61.10
ccc_c	84	29.91

Table S132: Hartree-Fock, RPA, DFT-SAPT(electrostatic+exchange+induction) part of the interaction energies in kJ/mol, cp-corrected cc-pVDZ basis set for the Polyene stack Relaxed series.

DZ		method		
System	D-Bonds	HF	DFT-SAPT ele	RPA exch
c2_c2	1	2.38	1.86	2.44
c4_c4	2	8.20	7.26	8.34
c6_c6	3	13.64	11.24	13.79
c8_c8	4	19.53	16.28	19.65
c10_c10	5	24.83	20.79	24.91
c12_c12	6	29.67	24.83	29.75
c14_c14	7	36.40	30.31	36.28

Table S133: Hartree-Fock, RPA, DFT-SAPT(electrostatic+exchange+induction) part of the interaction energies in kJ/mol, cp-corrected cc-pVTZ basis set for the Polyene stack Relaxed series.

TZ		method		
System	D-Bonds	HF	DFT-SAPT ele	RPA exch
c2_c2	1	2.64	2.03	2.74
c4_c4	2	8.12	7.20	8.36
c6_c6	3	13.32	11.05	13.65
c8_c8	4	19.02	15.94	19.41
c10_c10	5	24.16	20.34	24.59
c12_c12	6	28.87	24.25	29.31
c14_c14	7	35.31	29.60	35.78

Table S134: Hartree-Fock, RPA, DFT-SAPT(electrostatic+exchange+induction) part of the interaction energies in kJ/mol, cp-corrected cc-pVQZ basis set for the Polyene stack Relaxed series.

QZ		method		
System	D-Bonds	HF	DFT-SAPT ele	RPA exch
c2_c2	1	2.62	2.00	2.73
c4_c4	2	8.05	7.14	8.33
c6_c6	3	13.25	10.96	13.63
c8_c8	4	18.95	15.84	19.41
c10_c10	5	24.08	20.22	24.61
c12_c12	6	28.73	24.11	29.33
c14_c14	7	35.16		35.83

Table S135: Hartree-Fock, RPA, DFT-SAPT(electrostatic+exchange+induction) part of the interaction energies in kJ/mol, cp-corrected cc-pVQZ basis set for the Polyene stack Relaxed series.

QZ		method		
System	D-Bonds	HF	DFT-SAPT ele	RPA exch
c2_c2	1	2.62	2.00	2.73
c4_c4	2	8.05	7.14	8.33
c6_c6	3	13.25	10.96	13.63
c8_c8	4	18.95	15.84	19.41
c10_c10	5	24.08	20.22	24.61
c12_c12	6	28.73	24.11	29.33
c14_c14	7	35.16		35.83

Table S136: Hartree-Fock, RPA part of the interaction energies in kJ/mol, cp-corrected aug-cc-pVQZ basis set for the Polyene stack Relaxed series.

aQZ		method	
System	D-Bonds	HF	RPA exch
c2_c2	1	2.57	2.70
c4_c4	2	7.97	8.25
c6_c6	3	13.14	13.52
c8_c8	4	18.81	19.27
c10_c10	5	23.92	
c12_c12	6	28.54	
c14_c14	7	34.94	

Table S137: Hartree-Fock, RPA, DFT-SAPT(electrostatic+exchange+induction) part of the interaction energies in kJ/mol, cp-corrected cc-pV5Z basis set for the Polyene stack Relaxed series.

5Z		method		
System	D-Bonds	HF	DFT-SAPT ele	RPA exch
c2_c2	1	2.58	1.95	2.68
c4_c4	2	7.98	7.03	8.23
c6_c6	3	13.15	10.81	13.49
c8_c8	4	18.83	15.65	19.23
c10_c10	5			24.39

Table S138: Hartree-Fock, RPA part of the interaction energies in kJ/mol, cp-corrected aug-cc-pV5Z basis set for the Polyene stack Relaxed series.

a5Z		method	
System	D-Bonds	HF	RPA exch
c2_c2	1	2.57	2.69
c4_c4	2	7.97	8.23
c6_c6	3	13.14	13.50
c8_c8	4	18.81	
c10_c10	5	23.92	
c12_c12	6	28.54	
c14_c14	7	34.95	

Table S139: Hartree-Fock, RPA part of the interaction energies in kJ/mol, cc-pVDZ basis set for the Polyene stack Relaxed series.

DZ		method	
System	D-Bonds	HF	RPA exch
c2_c2	1	1.33	1.39
c4_c4	2	5.12	5.26
c6_c6	3	9.11	9.28
c8_c8	4	13.63	13.80
c10_c10	5	17.68	17.85
c12_c12	6	21.31	21.50
c14_c14	7	26.74	26.81

Table S140: Hartree-Fock, RPA part of the interaction energies in kJ/mol, cc-pVTZ basis set for the Polyene stack Relaxed series.

TZ		method	
System	D-Bonds	HF	RPA exch
c2_c2	1	2.01	2.13
c4_c4	2	6.93	7.21
c6_c6	3	11.67	12.08
c8_c8	4	16.19	17.45
c10_c10	5	20.71	22.27
c12_c12	6	25.92	26.63
c14_c14	7	32.03	32.80

Table S141: Hartree-Fock, RPA part of the interaction energies in kJ/mol, cc-pVQZ basis set for the Polyene stack Relaxed series.

QZ		method	
System	D-Bonds	HF	RPA exch
c2_c2	1	2.39	2.52
c4_c4	2	7.63	7.98
c6_c6	3	12.66	13.18
c8_c8	4	18.20	18.88
c10_c10	5	23.19	24.01
c12_c12	6	27.70	28.67
c14_c14	7	34.10	35.17

Table S142: Hartree-Fock, RPA part of the interaction energies in kJ/mol, aug-cc-pVQZ basis set for the Polyene stack Relaxed series.

aQZ		method	
System	D-Bonds	HF	RPA exch
c2_c2	1	2.52	2.67
c4_c4	2	7.85	8.22
c6_c6	3	12.95	13.48
c8_c8	4	18.56	19.24
c10_c10	5	23.60	
c12_c12	6	28.17	
c14_c14	7	34.62	

Table S143: Hartree-Fock, RPA part of the interaction energies in kJ/mol, cc-pV5Z basis set for the Polyene stack Relaxed series.

5Z		method	
System	D-Bonds	HF	RPA exch
c2_c2	1	2.55	2.67
c4_c4	2	7.91	8.20
c6_c6	3	13.05	13.45
c8_c8	4	18.69	19.19
c10_c10	5		24.34

Table S144: Hartree-Fock, RPA part of the interaction energies in kJ/mol, aug-cc-pV5Z basis set for the Polyene stack Relaxed series.

a5Z		method	
System	D-Bonds	HF	RPA exch
c2_c2	1	2.56	2.68
c4_c4	2	7.95	8.23
c6_c6	3	13.11	13.51
c8_c8	4	18.78	
c10_c10	5	23.88	
c12_c12	6	28.49	
c14_c14	7	35.01	

Table S145: Hartree-Fock, RPA, DFT-SAPT(electrostatic+exchange+induction) part of the interaction energies in kJ/mol, cp-corrected cc-pVDZ basis set for the Polyene stack Fixed series.

DZ		method		
System	D-Bonds	HF	DFT-SAPT ele	RPA exch
c2_c2	1	2.38	1.86	2.44
c4_c4	2	4.04	3.25	4.14
c6_c6	3	5.68	4.59	5.81
c8_c8	4	7.31	5.92	7.47
c10_c10	5	8.96	7.25	9.16
c12_c12	6	10.61	8.57	10.85
c14_c14	7	12.33	9.88	12.54

Table S146: Hartree-Fock, RPA, DFT-SAPT(electrostatic+exchange+induction) part of the interaction energies in kJ/mol, cp-corrected cc-pVTZ basis set for the Polyene stack Fixed series.

TZ		method		
System	D-Bonds	HF	DFT-SAPT ele	RPA exch
c2_c2	1	2.64	2.03	2.74
c4_c4	2	4.23	3.40	4.38
c6_c6	3	5.80	4.72	6.00
c8_c8	4	7.37	6.03	7.63
c10_c10	5	8.96	7.35	9.27
c12_c12	6	10.60	8.65	10.92
c14_c14	7	12.21	9.93	12.58

Table S147: Hartree-Fock, RPA, DFT-SAPT(electrostatic+exchange+induction) part of the interaction energies in kJ/mol, cp-corrected cc-pVQZ basis set for the Polyene stack Fixed series.

QZ		method		
System	D-Bonds	HF	DFT-SAPT ele	RPA exch
c2_c2	1	2.62	2.00	2.73
c4_c4	2	4.16	3.33	4.32
c6_c6	3	5.68	4.61	5.91
c8_c8	4	7.21	5.88	7.50
c10_c10	5	8.76	7.16	9.11
c12_c12	6	10.31	8.42	10.72
c14_c14	7	11.86		12.34

Table S148: Hartree-Fock, RPA part of the interaction energies in kJ/mol, cp-corrected aug-cc-pVQZ basis set for the Polyene stack Fixed series.

aQZ		method	
System	D-Bonds	HF	RPA exch
c2_c2	1	2.57	2.70
c4_c4	2	4.07	4.25
c6_c6	3	5.57	5.80
c8_c8	4	7.07	7.35
c10_c10	5	8.59	
c12_c12	6	10.12	
c14_c14	7	11.65	

Table S149: Hartree-Fock, RPA, DFT-SAPT(electrostatic+exchange+induction) part of the interaction energies in kJ/mol, cp-corrected cc-pV5Z basis set for the Polyene stack Fixed series.

5Z		method		
System	D-Bonds	HF	DFT-SAPT ele	RPA exch
c2_c2	1	2.58	1.95	2.68
c4_c4	2	4.09	3.25	4.24
c6_c6	3	5.59	4.51	5.79
c8_c8	4	7.09	5.75	7.34
c10_c10	5			8.92

Table S150: Hartree-Fock, RPA part of the interaction energies in kJ/mol, cp-corrected aug-cc-pV5Z basis set for the Polyene stack Fixed series.

a5Z		method	
System	D-Bonds	HF	RPA exch
c2_c2	1	2.57	2.69
c4_c4	2	4.07	4.24
c6_c6	3	5.57	5.79
c8_c8	4	7.07	
c10_c10	5	8.59	
c12_c12	6	10.12	
c14_c14	7	11.65	

Table S151: Hartree-Fock, RPA part of the interaction energies in kJ/mol, cc-pVDZ basis set for the Polyene stack Fixed series.

DZ		method	
System	D-Bonds	HF	RPA exch
c2_c2	1	1.33	1.39
c4_c4	2	2.28	2.37
c6_c6	3	3.25	3.37
c8_c8	4	4.23	4.38
c10_c10	5	5.22	5.42
c12_c12	6	6.23	6.46
c14_c14	7	7.28	7.52

Table S152: Hartree-Fock, RPA part of the interaction energies in kJ/mol, cc-pVTZ basis set for the Polyene stack Fixed series.

TZ		method	
System	D-Bonds	HF	RPA exch
c2_c2	1	2.01	2.13
c4_c4	2	3.27	3.42
c6_c6	3	4.54	4.76
c8_c8	4	5.10	6.12
c10_c10	5	6.22	7.50
c12_c12	6	8.48	8.89
c14_c14	7	9.87	10.37

Table S153: Hartree-Fock, RPA part of the interaction energies in kJ/mol, cc-pVQZ basis set for the Polyene stack Fixed series.

QZ		method	
System	D-Bonds	HF	RPA exch
c2_c2	1	2.39	2.52
c4_c4	2	3.81	4.01
c6_c6	3	5.22	5.51
c8_c8	4	6.65	7.03
c10_c10	5	8.03	8.58
c12_c12	6	9.56	10.14
c14_c14	7	11.09	11.78

Table S154: Hartree-Fock, RPA part of the interaction energies in kJ/mol, aug-cc-pVQZ basis set for the Polyene stack Fixed series.

aQZ		method	
System	D-Bonds	HF	RPA exch
c2_c2	1	2.52	2.67
c4_c4	2	3.98	4.22
c6_c6	3	5.41	5.72
c8_c8	4	6.88	7.32
c10_c10	5	8.38	
c12_c12	6	9.87	
c14_c14	7	11.43	

Table S155: Hartree-Fock, RPA part of the interaction energies in kJ/mol, cc-pV5Z basis set for the Polyene stack Fixed series.

5Z		method	
System	D-Bonds	HF	RPA exch
c2_c2	1	2.55	2.67
c4_c4	2	4.03	4.21
c6_c6	3	5.52	5.76
c8_c8	4	6.99	7.30
c10_c10	5		8.88

Table S156: Hartree-Fock, RPA part of the interaction energies in kJ/mol, aug-cc-pV5Z basis set for the Polyene stack Fixed series.

a5Z		method	
System	D-Bonds	HF	RPA exch
c2_c2	1	2.56	2.68
c4_c4	2	4.06	4.23
c6_c6	3	5.52	5.75
c8_c8	4	7.03	
c10_c10	5	8.56	
c12_c12	6	10.09	
c14_c14	7	11.69	

Electronic Structure Methods

In this section, we show all raw data for the different series using different methods (other than CCSD(T)) and basis sets for the acene-acene, (acene)-(acene-2) series, as well as the polyene stack series using relaxed and fixed ethylene-ethylene distances. For DFT-SAPT using the PBE0-AC

functional as underlying method, the dispersion contribution (including exchange-dispersion) is reported.

Table S157: Interaction energies (in kJ/mol) for different methods for the acene series using a cp-corrected cc-pVDZ basis set.

	Cx_Cx		correlation							
System	Rings	HF	MP2	MP3	MP2.5	dRPA	LNO-CCSD(T)	DFT-SAPT	CCSD	CCSD(T)
							vvtight	disp		
c_c	1	15.79	-20.08	-12.92	-16.50	-14.28	-21.12	-11.20	-13.01	-15.28
c2_c2	2	28.68	-44.26	-24.82	-34.54	-29.82	-40.85	-27.11	-26.84	-31.95
c3_c3	3	40.79	-69.69	-35.74	-52.71	-45.55	-52.11	-42.37	-40.77	-48.85
c4_c4	4	52.43	-95.66	-46.00	-70.83	-61.28	-68.50	-57.65	-54.66	-65.77
c5_c5	5	64.06	-122.17	-56.03	-89.10	-77.20	-85.33	-73.04	-68.79	-83.02
c6_c6	6	75.66	-148.82	-65.84	-107.33	-93.21	-105.13	-88.36		

Table S158: Interaction energies (in kJ/mol) for more methods for the acene series using a cp-corrected cc-pVDZ basis set.

DZ	Cx_Cx	correlation				
System	Rings	CCSD(cT-fit)	CCSD(T)-L	CCSDT-2	CCSDT	CCSDT(Q)
c_c	1	-14.89	-15.13	-14.91	-14.46	-14.96
c2_c2	2	-30.98	-31.63	-31.08	-30.38	-31.28

Table S159: Interaction energies (in kJ/mol) for different methods for the (acene)-(acene-2) series using a cp-corrected cc-pVDZ basis set.

TZ	Cx_Cx		correlation								
System	Rings	HF	MP2	MP3	MP2.5	dRPA	LNO-CCSD(T)	DFT-SAPT	CCSD	CCSD(T)	CCSD(cT-fit)
							vvtight	disp			
c_c	1	15.40	-25.12	-15.79	-20.46	-17.67	-5.65	-16.91	-15.71	-19.05	-18.45
c2_c2	2	27.77	-54.72	-30.03	-42.37	-36.77	-15.85	-36.16	-32.41	-39.70	-38.26
c3_c3	3	39.90	-85.69	-43.08	-64.38	-56.14	-24.60	-55.61	-49.26	-60.68	-58.33
c4_c4	4	51.57	-117.24	-55.38	-86.31	-75.46		-75.01	-66.04	-81.65	-78.33
c5_c5	5	63.24	-149.38			-95.03		-94.52			
c6_c6	6	74.86	-181.68			-114.57		-113.95			

Table S160: Interaction energies (in kJ/mol) for different methods for the acene series using a cp-corrected cc-pVTZ basis set.

TZ	Cx_Cx		correlation								
System	Rings	HF	MP2	MP3	MP2.5	dRPA	LNO-CCSD(T)	DFT-SAPT	CCSD	CCSD(T)	CCSD(cT-fit)
							vvtight	disp			
c_c	1	15.40	-25.12	-15.79	-20.46	-17.67	-5.65	-16.91	-15.71	-19.05	-18.45
c2_c2	2	27.77	-54.72	-30.03	-42.37	-36.77	-15.85	-36.16	-32.41	-39.70	-38.26
c3_c3	3	39.90	-85.69	-43.08	-64.38	-56.14	-24.60	-55.61	-49.26	-60.68	-58.33
c4_c4	4	51.57	-117.24	-55.38	-86.31	-75.46		-75.01	-66.04	-81.65	-78.33
c5_c5	5	63.24	-149.38			-95.03		-94.52			
c6_c6	6	74.86	-181.68			-114.57		-113.95			

Table S161: Interaction energies (in kJ/mol) for different methods for the (acene)-(acene-2) series using a cp-corrected cc-pVTZ basis set.

TZ	Cx_C(x-2)		correlation					
System		HF	MP2	MP3	MP2.5	dRPA	LNO-CCSD(T)	DFT-SAPT
							vvtight	disp
c3_c1	36	9.40	-20.72	-11.18	-15.95	-13.73	-15.12	-14.29
c4_c2	48	13.48	-32.41	-16.03	-24.22	-21.02	-33.93	-21.69
c5_c3	60	18.45	-45.41	-21.34	-33.38	-29.14		-29.93
c6_c4	72	22.40	-57.02			-36.18		-37.01
c7_c5	84	28.64	-72.59			-45.83		-46.77

Table S162: Interaction energies (in kJ/mol) for different methods for the acene series using a cp-corrected cc-pVQZ basis set.

QZ			correlation								
System	Rings	HF	MP2	MP3	MP2.5	dRPA	LNO-CCSD(T)	DFT-SAPT	CCSD	CCSD(T)	CCSD(cT-fit)
							vvtight	disp			
c_c	1	14.82	-26.86	-17.07	-21.96	-18.97	-8.03	-18.65	-16.77	-20.55	-19.87
c2_c2	2	27.70	-58.09	-32.33	-45.21	-39.32	-16.80	-39.35	-34.49	-42.64	-41.04
c3_c3	3	39.87	-90.68			-59.85		-60.16			
c4_c4	4	51.57	-123.84			-80.32		-80.89			
c5_c5	5	63.26	-157.62			-100.94		-101.72			

Table S163: Interaction energies (in kJ/mol) for different methods for the acene series using a cc-pVDZ basis set.

DZ	Cx_Cx		correlation						
System	Rings	HF	MP2	MP3	MP2.5	dRPA	LNO-CCSD(T)	CCSD	CCSD(T)
							vvtight		
c_c	1	11.56	-21.81	-14.09	-17.95	-16.20	-17.09	-14.01	-16.66
c2_c2	2	22.31	-48.97	-28.23	-38.60	-35.20	-37.09	-29.94	-35.97
c3_c3	3	32.28	-77.39	-41.37	-59.38	-54.43	-57.09	-45.99	-55.54
c4_c4	4	41.80	-106.32	-53.83	-80.07	-73.63	-77.40	-61.95	-75.09
c5_c5	5	51.40	-135.79	-66.05	-100.92	-93.01	-97.92	-78.20	-95.00
c6_c6	6	60.78	-165.36	-78.00	-121.68	-112.46	-118.30		

Table S164: Interaction energies (in kJ/mol) for more methods for the acene series using a cc-pVDZ basis set.

System	Rings	CCSD(cT-fit)	CCSDT	CCSDT(Q)
c_c	1	-16.20	-15.65	-16.25
c2_c2	2	-34.84	-34.18	-35.18
c3_c3	3	-53.67		
c4_c4	4	-72.43		
c5_c5	5	-91.55		

Table S165: Interaction energies (in kJ/mol) for different methods for the (acene)-(acene-2) series using a cc-pVDZ basis set.

DZ	Cx_C(x-2)		correlation							
System	Atoms	HF	MP2	MP3	MP2.5	dRPA	LNO-CCSD(T)	CCSD	CCSD(T)	CCSD(cT-fit)
							vvtight			
c3_c1	36	7.27	-18.82	-10.79	-14.80	-13.64	-14.03	-11.45	-13.78	-13.34
c4_c2	48	10.54	-29.53	-15.67	-22.60	-20.86	-21.65	-17.45	-21.11	-20.39
c5_c3	60	14.57	-41.51	-21.07	-31.29	-29.01	-30.14	-24.16	-29.32	-28.28
c6_c4	72	17.75	-52.07	-25.45	-38.76	-35.93	-37.47	-29.97	-36.44	-35.11
c7_c5	84	22.81	-66.50	-31.73	-49.12	-45.74	-47.51			

Table S166: Interaction energies (in kJ/mol) for different methods for the acene series using a cc-pVTZ basis set.

TZ	Cx_Cx		correlation							
System	Rings	HF	MP2	MP3	MP2.5	dRPA	LNO-CCSD(T)	CCSD	CCSD(T)	CCSD(cT-fit)
							vvtight			
c_c	1	13.39	-26.99	-17.29	-22.14	-20.48	-21.03	-17.04	-20.72	-20.06
c2_c2	2	25.40	-59.22	-33.75	-46.48	-43.60	-44.68	-35.80	-43.84	-42.31
c3_c3	3	36.79	-92.92	-49.11	-71.02	-67.10	-68.83	-54.83	-67.43	-64.93
c4_c4	4	47.71	-127.19	-63.70	-95.45	-90.57		-73.75	-90.95	-87.44
c5_c5	5	61.56	-163.71			-114.26				
c6_c6	6	69.50	-197.01			-137.87				

Table S167: Interaction energies (in kJ/mol) for different methods for the (acene)-(acene-2) series using a cc-pVTZ basis set.

TZ	Cx_C(x-2)		correlation							
System	Atoms	HF	MP2	MP3	MP2.5	dRPA	LNO-CCSD(T)	CCSD	CCSD(T)	CCSD(cT-fit)
							vvtight			
c3_c1	36	8.46	-22.68	-12.81	-17.74	-16.67	-16.93	-13.67	-16.71	-16.13
c4_c2	48	12.26	-35.40	-18.53	-26.96	-25.52	-26.05	-20.82	-25.59	-24.64
c5_c3	60	16.91	-49.56	-24.81	-37.18	-35.37				
c6_c4	72	20.59	-62.14			-43.89				
c7_c5	84	26.46	-79.09			-55.64				

Table S168: Interaction energies (in kJ/mol) for different methods for the acene series using a cc-pVQZ basis set.

QZ	Cx_Cx		correlation							
System	Rings	HF	MP2	MP3	MP2.5	dRPA	LNO-CCSD(T)	CCSD	CCSD(T)	CCSD(cT-fit)
							vvtight			
c_c	1	14.17	-27.82	-17.72	-22.77	-20.59	-21.09	-17.36	-21.30	-20.58
c2_c2	2	26.74	-60.30	-33.88	-47.09	-43.06	-44.12	-35.91	-44.40	-42.74
c3_c3	3	38.63	-94.18			-65.84				
c4_c4	4	50.05	-128.64			-88.57				
c5_c5	5	61.56	-163.71			-111.45				
c6_c6	6					-134.45				

Table S169: Interaction energies (in kJ/mol) for different methods for the Polyene stack series in Relaxed geometry using a cp-corrected cc-pVDZ basis set.

	Cx_Cx		correlation							
System	D-Bonds	HF	MP2	MP3	MP2.5	dRPA	LNO-CCSD(T)	DFT-SAPT	CCSD	CCSD(T)
							vvTight	disp		
c2_c2	1	2.38	-1.92	-1.74	-1.83	-1.69	-1.80	-1.13	-1.59	-1.78
c4_c4	2	8.20	-8.45	-6.94	-7.70	-6.98	-7.38	-5.98	-6.33	-7.24
c6_c6	3	13.64	-15.89	-12.30	-14.10	-12.81	-13.42	-10.81	-11.36	-13.15
c8_c8	4	19.53	-24.23	-18.04	-21.14	-19.29	-20.03	-16.70	-16.86	-19.63
c10_c10	5	24.83	-32.29	-23.41	-27.85	-25.51	-26.47	-22.40	-22.07	-25.81
c12_c12	6	29.67	-39.92	-28.49	-34.21	-31.46	-32.46	-27.81	-26.99	-31.65
c14_c14	7	36.40	-49.34	-34.80	-42.07	-38.73	-39.87	-34.42	-33.11	-38.90

Table S170: Interaction energies (in kJ/mol) for more methods for the Polyene stack series in Relaxed geometry using a cp-corrected cc-pVDZ basis set.

DZ	Cx_Cx	correlation				
System	D-Bonds	CCSD(T)-L	CCSDT-2	CCSDT-3	CCSDT	CCSDT(Q)
c2_c2	1	-1.76	-1.75	-1.75	-1.76	-1.78
c4_c4	2	-7.16	-7.10	-7.11	-7.11	-7.27
c6_c6	3	-12.99	-12.86	-12.88	-12.85	
c8_c8	4	-19.39	-19.18	-19.23		

Table S171: Interaction energies (in kJ/mol) for different methods for the Polyene stack series in Relaxed geometry using a cp-corrected cc-pVTZ basis set.

	Cx_Cx		correlation							
System	D-Bonds	HF	MP2	MP3	MP2.5	dRPA	LNO-CCSD(T)	DFT-SAPT	CCSD	CCSD(T)
							vvTight	disp		
c2_c2	1	2.64	-2.68	-2.38	-2.53	-2.26	-2.44	-1.82	-2.09	-2.42
c4_c4	2	8.12	-11.08	-8.88	-9.98	-8.94	-9.49	-8.78	-7.91	-9.37
c6_c6	3	13.32	-20.49	-15.49	-17.99	-16.25	-17.12	-15.37	-14.07	-16.83
c8_c8	4	19.02	-30.95	-22.55	-26.75	-24.28	-25.56	-23.25	-20.78	-25.01
c10_c10	5	24.16	-41.03	-29.15	-35.09	-32.03	-33.59	-30.83	-27.14	-32.78
c12_c12	6	28.87	-50.56	-35.38	-42.97	-39.38	-41.11	-38.03	-33.14	-40.13
c14_c14	7	35.31	-62.15	-42.95	-52.55	-48.30	-50.16	-46.72	-40.48	-49.09

Table S172: Interaction energies (in kJ/mol) for different methods for the Polyene stack series in Relaxed geometry using a cp-corrected cc-pVQZ basis set.

	Cx_Cx		correlation							
System	D-Bonds	HF	MP2	MP3	MP2.5	dRPA	LNO-CCSD(T)	DFT-SAPT	CCSD	CCSD(T)
							vvTight	disp		
c2_c2	1	2.62	-2.99	-2.66	-2.83	-2.50	-2.70	-2.16	-2.29	-2.69
c4_c4	2	8.05	-12.04	-9.69	-10.87	-9.73	-10.36	-9.89	-8.54	-10.23
c6_c6	3	13.25	-22.11	-16.82	-19.46	-17.58	-18.59	-17.09	-15.12	-18.29
c8_c8	4	18.95	-33.27	-24.40	-28.83	-26.18	-27.59	-25.67	-22.27	-27.09
c10_c10	5	24.08	-44.00			-34.45	-36.19	-33.90	-29.05	-35.45
c12_c12	6	28.73	-54.16			-42.31	-44.31	-41.71	-35.44	
c14_c14	7					-51.88	-54.26			

Table S173: Interaction energies (in kJ/mol) for different methods for the Polyene stack series in Relaxed geometry using a cp-corrected cc-pV5Z basis set.

	Cx_Cx		correlation							
System	D-Bonds	HF	MP2	MP3	MP2.5	dRPA	LNO-CCSD(T)	DFT-SAPT	CCSD	CCSD(T)
							vvTight	disp		
c2_c2	1	2.58	-3.13			-2.63	-2.84	-2.35	-2.39	-2.83
c4_c4	2	7.98	-12.49			-10.14	-10.78	-10.46	-8.84	-10.65
c6_c6	3	13.15	-22.83			-18.23	-19.21	-17.92		
c8_c8	4	18.83				-27.13	-28.49	-26.82		

Table S174: Interaction energies (in kJ/mol) for different methods for the Polyene stack series in Fixed geometry using a cp-corrected cc-pVDZ basis set.

	Cx_Cx		correlation							
System	D-Bonds	HF	MP2	MP3	MP2.5	dRPA	LNO-CCSD(T)	DFT-SAPT	CCSD	CCSD(T)
							vvTight	disp		
c2_c2	1	2.38	-1.92	-1.74	-1.83	-1.69	-1.80	-1.13	-1.59	-1.78
c4_c4	2	4.04	-4.80	-3.93	-4.36	-3.93	-4.14	-3.11	-3.58	-4.09
c6_c6	3	5.68	-8.08	-6.24	-7.16	-6.47	-6.78	-5.42	-5.75	-6.64
c8_c8	4	7.31	-11.52	-8.58	-10.05	-9.14	-9.56	-7.86	-7.98	-9.27
c10_c10	5	8.96	-15.04	-10.94	-12.99	-11.92	-12.37	-10.55	-10.23	-11.94
c12_c12	6	10.61	-18.59	-13.31	-15.95	-14.72	-15.17	-12.91	-12.51	-14.64
c14_c14	7	12.33	-22.18	-15.70	-18.94	-17.57	-17.98	-15.47	-14.81	-17.37

Table S175: Interaction energies (in kJ/mol) for more methods for the Polyene stack series in Fixed geometry using a cp-corrected cc-pVDZ basis set.

DZ	Cx_Cx	correlation				
System	D-Bonds	CCSD(T)-L	CCSDT-2	CCSDT-3	CCSDT	CCSDT(Q)
c2_c2	-1.78	-1.76	-1.75	-1.75	-1.76	-1.78
c4_c4	-4.09	-4.05	-4.01	-4.01	-4.01	-4.10
c6_c6	-6.64	-6.56	-6.48	-6.50	-6.47	
c8_c8	-9.27	-9.16	-9.04	-9.06		

Table S176: Interaction energies (in kJ/mol) for different methods for the Polyene stack series in Fixed geometry using a cp-corrected cc-pVTZ basis set.

	Cx_Cx		correlation							
System	D-Bonds	HF	MP2	MP3	MP2.5	dRPA	LNO-CCSD(T)	DFT-SAPT	CCSD	CCSD(T)
							vvTight	disp		
c2_c2	1	2.64	-2.68	-2.38	-2.53	-2.26	-2.44	-1.82	-2.09	-2.42
c4_c4	2	4.23	-6.45	-5.16	-5.81	-5.14	-5.55	-4.69	-4.58	-5.43
c6_c6	3	5.80	-10.68	-8.09	-9.39	-8.37	-9.00	-7.92	-7.29	-8.73
c8_c8	4	7.37	-15.10	-11.06	-13.08	-11.76	-12.53	-11.28	-10.07	-12.14
c10_c10	5	8.96	-19.60	-14.02	-16.81	-15.25	-16.20	-14.72	-12.88	-15.59
c12_c12	6	10.60	-24.13	-17.00	-20.56	-18.80	-19.84	-18.19	-15.70	-19.07
c14_c14	7	12.21	-28.68	-19.97	-24.33	-22.34	-23.35	-21.67	-18.53	-22.55

Table S177: Interaction energies (in kJ/mol) for different methods for the Polyene stack series in Fixed geometry using a cp-corrected cc-pVQZ basis set.

	Cx_Cx		correlation							
System	D-Bonds	HF	MP2	MP3	MP2.5	dRPA	LNO-CCSD(T)	DFT-SAPT	CCSD	CCSD(T)
							vvTight	disp		
c2_c2	1	2.62	-2.99	-2.66	-2.83	-2.50	-2.70	-2.16	-2.29	-2.69
c4_c4	2	4.16	-7.08	-5.70	-6.39	-5.61	-6.05	-5.38	-4.99	-5.99
c6_c6	3	5.68	-11.64	-8.88	-10.26	-9.11	-9.75	-8.95	-7.89	-9.58
c8_c8	4	7.21	-16.38	-12.09	-14.23	-12.75	-13.59	-12.67	-10.86	-13.28
c10_c10	5	8.76	-21.21			-16.49	-17.47	-16.45	-13.87	-17.03
c12_c12	6	10.31	-26.07			-20.29	-21.49	-20.26		
c14_c14	7					-24.11	-25.40			

Table S178: Interaction energies (in kJ/mol) for different methods for the Polyene stack series in Fixed geometry using a cp-corrected cc-pV5Z basis set.

	Cx_Cx		correlation							
System	D-Bonds	HF	MP2	MP3	MP2.5	dRPA	LNO-CCSD(T)	DFT-SAPT	CCSD	CCSD(T)
							vvTight	disp		
c2_c2	1	2.58	-3.13			-2.63	-2.84	-2.35	-2.39	-2.83
c4_c4	2	4.09	-7.34			-5.85	-6.31	-5.71	-5.15	-6.23
c6_c6	3	5.59	-12.01			-9.44	-10.10	-9.41		
c8_c8	4	7.09				-13.19	-14.01	-13.26		

Table S179: Interaction energies (in kJ/mol) for different methods for the Polyene stack series in Relaxed geometry using a cp-uncorrected cc-pVDZ basis set.

DZ	Cx_Cx		correlation							
System	D-Bonds	HF	MP2	MP3	MP2.5	dRPA	LNO-CCSD(T)	CCSD	CCSD(T)	CCSD(cT-fit)
							vvtight			
c2_c2	1	1.33	-1.87	-1.61	-1.74	-1.52	-1.64	-1.43	-1.63	-1.61
c4_c4	2	5.12	-8.90	-7.03	-7.97	-7.18	-7.57	-6.31	-7.35	-7.20
c6_c6	3	9.11	-17.07	-12.87	-14.97	-13.79	-14.12	-11.74	-13.80	-13.49
c8_c8	4	13.63	-26.30	-19.24	-22.77	-21.27	-21.53	-17.76	-21.00	-20.49
c10_c10	5	17.68	-35.18	-25.20	-30.19	-28.46	-28.57	-23.49	-27.86	-27.15
c12_c12	6	21.31	-43.55	-30.78	-37.16	-35.24	-35.27	-28.84	-34.28	-33.38
c14_c14	7	26.74	-53.81	-37.60	-45.71	-43.24	-43.23	-35.33	-42.02	-40.91

Table S180: Interaction energies (in kJ/mol) for more methods for the Polyene stack series in Relaxed geometry using a cp-uncorrected cc-pVDZ basis set.

DZ	Cx_Cx	correlation				
System	D-Bonds	CCSD(T)-L	CCSDT-2	CCSDT-3	CCSDT	CCSDT(Q)
c2_c2	1	-1.62	-1.61	-1.61	-1.61	-1.64
c4_c4	2	-7.27	-7.20	-7.21	-7.20	-7.36
c6_c6	3	-13.64	-13.49	-13.52	-13.46	
c8_c8	4	-20.74	-20.50	-20.56		

Table S181: Interaction energies (in kJ/mol) for different methods for the Polyene stack series in Relaxed geometry using a cp-uncorrected cc-pVTZ basis set.

TZ	Cx_Cx		correlation							
System	D-Bonds	HF	MP2	MP3	MP2.5	dRPA	LNO-CCSD(T)	CCSD	CCSD(T)	CCSD(cT-fit)
							vvtight			
c2_c2	1	2.01	-2.72	-2.31	-2.52	-2.22	-2.39	-2.01	-2.37	-2.32
c4_c4	2	6.93	-11.81	-9.33	-10.57	-9.88	-10.03	-8.28	-9.90	-9.66
c6_c6	3	11.67	-21.96	-16.57	-19.26	-18.35	-18.34	-14.99	-18.04	-17.57
c8_c8	4	16.19	-33.30	-24.35	-28.83	-27.69	-27.51	-22.33	-27.00	-26.25
c10_c10	5	20.71	-44.17	-31.62	-37.90	-36.69	-36.24	-29.29	-35.51	-34.49
c12_c12	6	25.92	-54.45	-38.46	-46.46	-45.14	-44.38	-35.82	-43.52	-42.25
c14_c14	7	32.03	-66.85	-46.65	-56.75	-55.14	-53.98	-43.70	-53.09	-51.52

Table S182: Interaction energies (in kJ/mol) for different methods for the Polyene stack series in Relaxed geometry using a cp-uncorrected cc-pVQZ basis set.

QZ	Cx_Cx		correlation							
System	D-Bonds	HF	MP2	MP3	MP2.5	dRPA	LNO-CCSD(T)	CCSD	CCSD(T)	CCSD(cT-fit)
							vvtight			
c2_c2	1	2.39	-3.04	-2.65	-2.84	-2.54	-2.71	-2.28	-2.69	-2.64
c4_c4	2	7.63	-12.45	-9.91	-11.18	-10.35	-10.64	-8.72	-10.50	-10.24
c6_c6	3	12.66	-22.90	-17.31	-20.11	-18.84	-19.16	-15.55	-18.87	-18.34
c8_c8	4	18.20	-34.48	-25.20	-29.84	-28.21	-28.51	-22.97	-28.00	-27.18
c10_c10	5	23.19	-45.61			-37.19	-37.40	-30.01	-36.69	-35.58
c12_c12	6	27.70	-56.14			-45.72	-45.79	-36.65		
c14_c14	7					-55.77	-55.79			

Table S183: Interaction energies (in kJ/mol) for different methods for the Polyene stack series in Relaxed geometry using a cp-uncorrected cc-pV5Z basis set.

5Z	Cx_Cx		correlation							
System	D-Bonds	HF	MP2	MP3	MP2.5	dRPA	LNO-CCSD(T)	CCSD	CCSD(T)	CCSD(cT-fit)
							vvtight			
c2_c2	1	2.55	-3.20			-2.75	-2.89	-2.42	-2.87	-2.81
c4_c4	2	7.91	-12.76			-10.64	-10.96	-8.98	-10.83	-10.56
c6_c6	3	13.05	-23.32			-19.17	-19.54			
c8_c8	4	18.69				-28.55	-29.02			

Table S184: Interaction energies (in kJ/mol) for different methods for the Polyene stack series in Fixed geometry using a cp-uncorrected cc-pVDZ basis set.

DZ	Cx_Cx		correlation							
System	D-Bonds	HF	MP2	MP3	MP2.5	dRPA	LNO-CCSD(T)	CCSD	CCSD(T)	CCSD(cT-fit)
							vvtight			
c2_c2	1	1.33	-1.87	-1.61	-1.74	-1.52	-1.64	-1.43	-1.63	-1.61
c4_c4	2	2.28	-4.85	-3.80	-4.33	-3.79	-4.09	-3.42	-3.96	-3.89
c6_c6	3	3.25	-8.24	-6.13	-7.19	-6.39	-6.78	-5.60	-6.55	-6.40
c8_c8	4	4.23	-11.79	-8.50	-10.15	-9.14	-9.54	-7.84	-9.22	-8.99
c10_c10	5	5.22	-15.42	-10.88	-13.15	-12.00	-12.36	-10.11	-11.93	-11.63
c12_c12	6	6.23	-19.09	-13.27	-16.18	-14.88	-15.27	-12.39	-14.67	-14.28
c14_c14	7	7.28	-22.85	-15.70	-19.28	-17.83	-18.11	-14.72	-17.45	-16.98

Table S185: Interaction energies (in kJ/mol) for more methods for the Polyene stack series in Fixed geometry using a cp-uncorrected cc-pVDZ basis set.

DZ	Cx_Cx	correlation				
System	D-Bonds	CCSD(T)-L	CCSDT-2	CCSDT-3	CCSDT	CCSDT(Q)
c2_c2	1	-1.62	-1.60	-1.61	-1.61	-1.64
c4_c4	2	-3.92	-3.88	-3.88	-3.88	-3.96
c6_c6	3	-6.47	-6.39	-6.40	-6.37	
c8_c8	4	-9.10	-8.98	-9.00		

Table S186: Interaction energies (in kJ/mol) for different methods for the Polyene stack series in Fixed geometry using a cp-uncorrected cc-pVTZ basis set.

TZ	Cx_Cx		correlation							
System	D-Bonds	HF	MP2	MP3	MP2.5	dRPA	LNO-CCSD(T)	CCSD	CCSD(T)	CCSD(cT-fit)
							vvtight			
c2_c2	1	2.01	-2.72	-2.31	-2.52	-2.22	-2.39	-2.01	-2.37	-2.32
c4_c4	2	3.27	-6.71	-5.23	-5.97	-5.35	-5.62	-4.62	-5.54	-5.40
c6_c6	3	4.54	-11.18	-8.31	-9.74	-8.89	-9.20	-7.44	-9.02	-8.76
c8_c8	4	5.10	-15.84	-11.43	-13.64	-12.58	-12.91	-10.36	-12.61	-12.23
c10_c10	5	6.22	-20.58	-14.56	-17.57	-16.39	-16.66	-13.29	-16.25	-15.74
c12_c12	6	8.48	-25.35	-17.68	-21.51	-20.22	-20.41	-16.24	-19.90	-19.27
c14_c14	7	9.87	-30.15	-20.78	-25.47	-24.08	-24.13	-19.21	-23.56	-22.80

Table S187: Interaction energies (in kJ/mol) for different methods for the Polyene stack series in Fixed geometry using a cp-uncorrected cc-pVQZ basis set.

QZ	Cx_Cx		correlation							
System	D-Bonds	HF	MP2	MP3	MP2.5	dRPA	LNO-CCSD(T)	CCSD	CCSD(T)	CCSD(cT-fit)
							vvtight			
c2_c2	1	2.39	-3.04	-2.65	-2.84	-2.54	-2.71	-2.28	-2.69	-2.64
c4_c4	2	3.81	-7.30	-5.79	-6.55	-5.91	-6.18	-5.06	-6.11	-5.95
c6_c6	3	5.22	-12.03	-9.09	-10.56	-9.66	-10.00	-8.06	-9.83	-9.55
c8_c8	4	6.65	-16.94	-12.38	-14.66	-13.60	-13.97	-11.13	-13.66	-13.24
c10_c10	5	8.03	-21.94			-17.63	-17.98	-14.24	-17.55	-16.98
c12_c12	6	9.56	-26.97			-21.72	-22.02	-17.36		
c14_c14	7					-25.83	-26.01			

Table S188: Interaction energies (in kJ/mol) for different methods for the Polyene stack series in Fixed geometry using a cp-uncorrected cc-pV5Z basis set.

5Z	Cx_Cx		correlation							
System	D-Bonds	HF	MP2	MP3	MP2.5	dRPA	LNO-CCSD(T)	CCSD	CCSD(T)	CCSD(cT-fit)
							vvtight			
c2_c2	1	2.55	-3.20			-2.75	-2.89	-2.42	-2.87	-2.81
c4_c4	2	4.03	-7.51			-6.16	-6.45	-5.26	-6.38	-6.21
c6_c6	3	5.52	-12.36			-9.99	-10.33			
c8_c8	4	6.99				-13.96	-14.41			

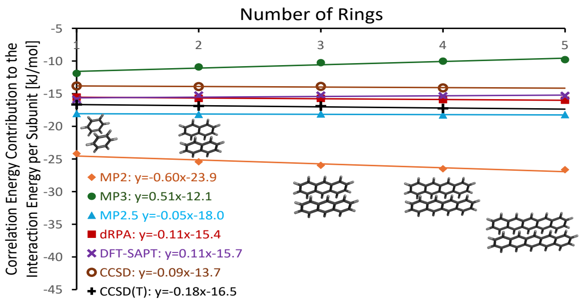

(a) Counterpoise-corrected DZ energies for the acene series.

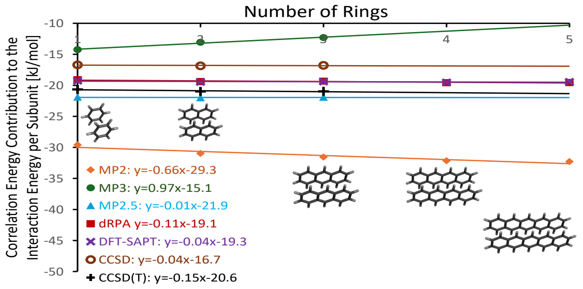

(b) Counterpoise-corrected TZ energies for the acene series.

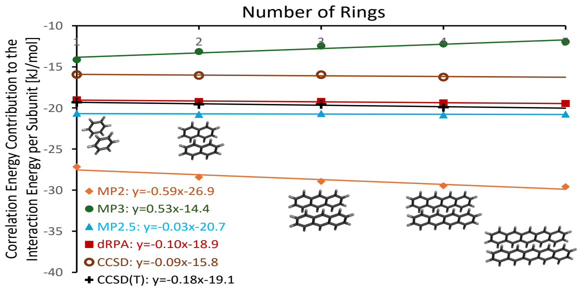

(c) Counterpoise-uncorrected DZ energies for the acene series.

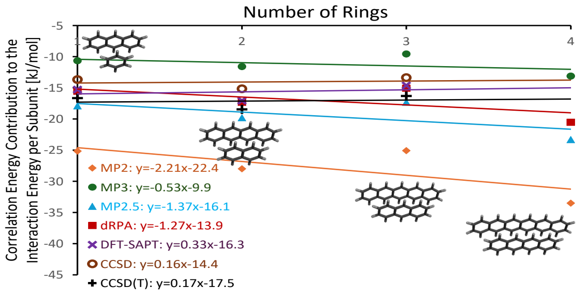

(d) Counterpoise-corrected DZ energies for the acene-acene-2 series.

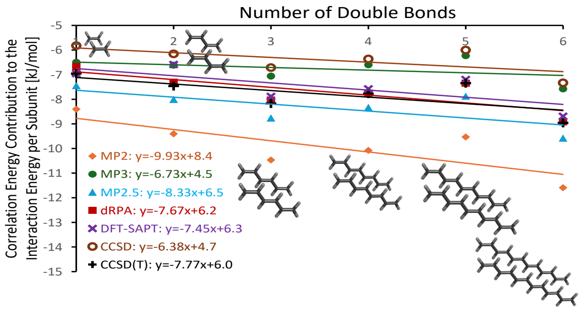

(e) Counterpoise-corrected TZ Energies for the ethylene stack series (relaxed geometry).

Figure S4: Difference in Correlation energies derived from approximate methods in kJ/mol vs. number of rings (acenes), number of atoms (acenes with acenes-2), or double-bonds (ethylene stacks).

Table S189: Slopes (in kJ/mol per segment) and Intercepts (in kJ/mol) for Figure 3.

Subfigure		Method	Slope	Intercept
a	DZ cp-corrected	MP2	-25.81	6.9
		MP3	-10.53	-3.4
		MP2.5	-18.17	1.8
		dRPA	-15.79	1.7
		DFT-SAPT	-15.40	3.9
		CCSD	-13.94	1.0
		CCSD(T)	-16.93	1.8
b	TZ cp-corrected	MP2	-31.38	7.5
		MP3	-13.18	-3.1
		MP2.5	-21.96	1.5
		dRPA	-19.39	1.9
		DFT-SAPT	-19.42	2.6
		CCSD	-16.79	1.1
		CCSD(T)	-20.88	1.9
c	DZ cp-uncorrected	MP2	-28.78	8.1
		MP3	-12.73	-2.4
		MP2.5	-20.75	2.9
		dRPA	-19.25	3.2
		CCSD	-16.04	2.1
		CCSD(T)	-19.58	3.1
d	DZ cp-corrected	MP2	-27.65	-14.0
		MP3	-11.09	-12.2
		MP2.5	-19.37	-13.1
		dRPA	-16.93	-10.8
		DFT-SAPT	-15.78	-12.5
		CCSD	-14.18	-11.5
		CCSD(T)	-17.26	-13.4
e	TZ cp-corrected	MP2	-9.93	8.4
		MP3	-6.73	4.5
		MP2.5	-8.33	6.5
		dRPA	-7.67	6.2
		DFT-SAPT	-7.45	6.3
		CCSD	-6.38	4.7
		CCSD(T)	-7.77	6.0

Table S190: Slopes (in kJ/mol per segment) and Intercepts (in kJ/mol) for Figure S3.

Subfigure		Method	Slope	Intercept
a	DZ cp-corrected	MP2	-0.60	-23.9
		MP3	0.51	-12.1
		MP2.5	-0.05	-18.0
		dRPA	-0.11	-15.4
		DFT-SAPT	0.11	-15.7
		CCSD	-0.09	-13.7
		CCSD(T)	-0.18	-16.5
b	TZ cp-corrected	MP2	-0.66	-29.3
		MP3	0.97	-15.1
		MP2.5	-0.01	-21.9
		dRPA	-0.11	-19.1
		DFT-SAPT	-0.04	-19.3
		CCSD	-0.04	-16.7
		CCSD(T)	-0.15	-20.6
c	DZ cp-uncorrected	MP2	-0.59	-26.9
		MP3	0.53	-14.4
		MP2.5	-0.03	-20.7
		dRPA	-0.10	-18.9
		CCSD	-0.09	-15.8
		CCSD(T)	-0.18	-19.1
d	DZ cp-corrected	MP2	-2.21	-22.4
		MP3	-0.53	-9.9
		MP2.5	-1.37	-16.1
		dRPA	-1.27	-13.9
		DFT-SAPT	0.33	-16.3
		CCSD	0.16	-14.4
		CCSD(T)	0.17	-17.5
e	TZ cp-corrected	MP2	-0.46	-8.3
		MP3	-0.11	-6.4
		MP2.5	-0.28	-7.4
		dRPA	-0.32	-6.6
		DFT-SAPT	-0.29	-6.5
		CCSD	-0.19	-5.7
		CCSD(T)	-0.27	-6.8

Rank-reduced Calculations for the Benzene and Naphtalene Dimers

Rank-reduced CCSDT and CCSDT(Q) calculations depend on two parameters ($N_{\rm svd}$ and $N_{\rm qua}$) that define the size of the excitation subspaces for triply- and quadruply-excited amplitudes. According to the recommendation from previous work [J. Chem. Theory Comput. 18, 6537-6556 (2022)], these parameters were related through the formula $N_{\rm qua} = \frac{2}{3}N_{\rm svd}$, which leaves $N_{\rm svd}$ as the only adjustable parameter. In calculation of the interaction energies, it is important that the values of this parameter are consistent between the dimer and the monomers. To assure that we applied the following procedure. The triple-excitation subspaces for the dimer and the monomers were found using the higher-order orthogonal iteration (HOOI) procedure as described elsewhere [J. Chem. Phys. 156, 064103 (2022)]. At each step of the HOOI algorithm, one calculates the singular value decomposition (SVD) of a certain rectangular matrix [see Eq. (49) in J. Chem. Phys. 156, 064103 (2022)]. Once the HOOI converged for the dimer, we calculated sum of squares of the first $N_{\rm svd}$ singular values ($\tau = \sum_k \sigma_k^2$) of this matrix for a given $N_{\rm svd}$. Next, when the HOOI procedure is used for the monomer, the size of the excitation subspace $N_{\rm svd}$ is adjusted to match half of the value of $\tau = \sum_k \sigma_k^2$ found for the dimer. In this way, for any given $N_{\rm svd}$ for the dimer, one is able to find the corresponding size of the excitation subspace for the monomer. Finally, to verify that the results are sufficiently stable with respect to the $N_{\rm svd}$, the calculations were repeated for a set of values of $N_{\rm svd}$ ranging from $N_{\rm MO}$ to $2N_{\rm MO}$, where $N_{\rm MO}$ is the number of active molecular orbitals in the system. The results are shown in Fig. S5 for the T-(T) correction and Fig. S6 for the (Q) correction. The uncertainty of the results was estimated as the standard deviation calculated from the five last points. Based on these results, best estimates for the T-(T) and (Q) correction are, respectively, 0.82(2) kJ/mol and $-0.5(2)$ kJ/mol for the benzene dimer, and 1.57(4) kJ/mol and $-0.9(4)$ kJ/mol for the naphtalene dimer. The numbers in parentheses in each case denote uncertainty estimates at the last digit shown. Note that these results correspond to the counterpoise-corrected calculations within the cc-pVDZ basis set.

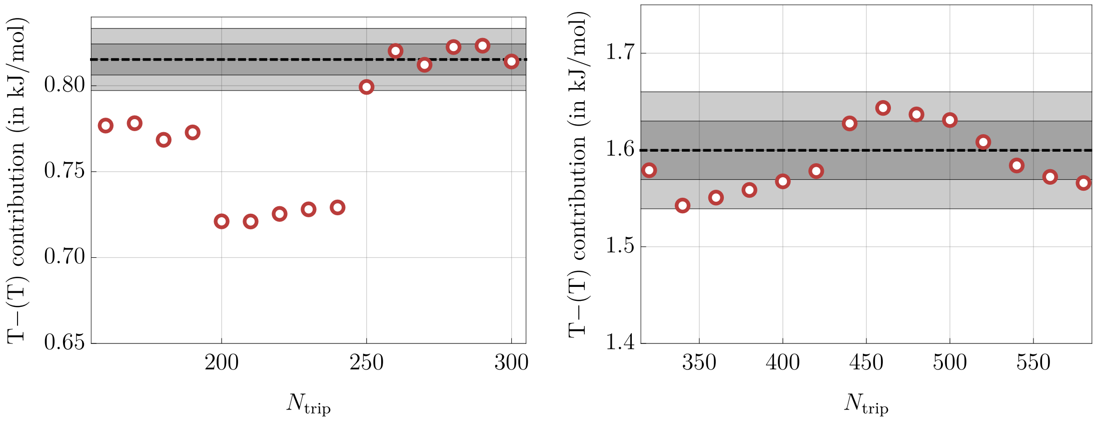

Figure S5: T-(T) correction for benzene dimer (left panel) and naphtalene dimer (right panel) as a function of N_{svd}. The shaded areas represent the estimated uncertainties at one sigma (dark grey) or two sigma (light grey) level.

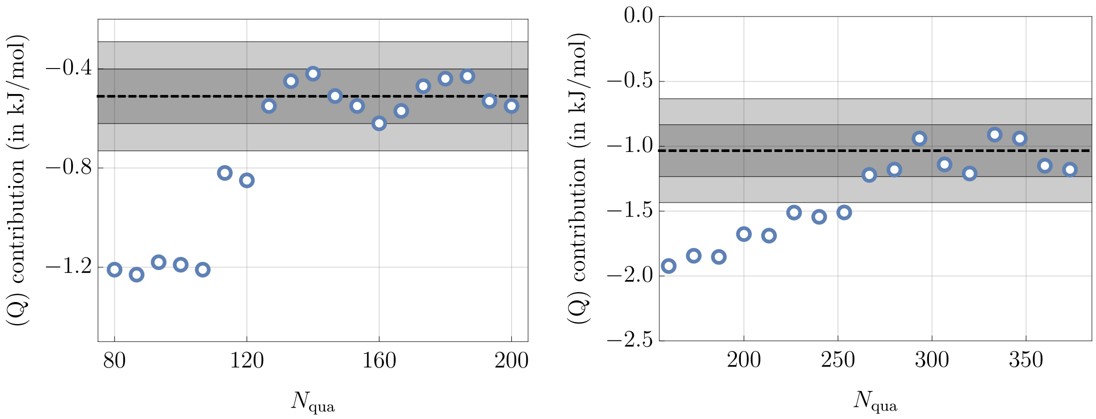

Figure S6: Same as Fig. S5 but for the (Q) correction.

Best Estimates of Slopes

We provide the correlation energy slopes of the fixed polyene stacks, as these are not displayed in the main text. They are about a factor of two smaller than the correlation interaction energies of the relaxed polyene stacks.

Table S191: Best available cp-corrected correlation energy slopes (kJ/mol per double bond), including CCSDT(Q) values, of the polyene stack fixed dimers. Numbers in italic are estimates.

		Method						
				CCSD(T)		post-CCSD(T)		
Slopes	Basis Set	MP2	CCSD	`vvTight`	canonical	CCSDT-2	CCSDT	CCSDT(Q)
1→2	DZ	-2.88	-1.99	-2.34	-2.31	-2.26	-2.26	-2.31
1→3	DZ	-3.08	-2.08	-2.49	-2.43	-2.37	-2.36	_-2.43_
1→4	DZ	-3.21	-2.13	-2.59	-2.50	-2.44	_-2.43_	_-2.50_
1→7	TZ	-4.37	-2.75	-3.52	-3.38	_-3.28_*	_-3.27_*	_-3.38_*
1→2	{T,Q}Z	-4.33	-2.83	-3.53	-3.51	_-3.43_*	_-3.42_*	_-3.51_*
1→4	{T,Q}Z	-4.71	-3.00	-3.83	-3.75	_-3.65_*	_-3.65_*	_-3.75_*
1→7	{T,Q}Z			-4.03	_-3.88_	_-3.79_*	_-3.78_*	_-3.88_*
1→2	a{Q,5}Z	-4.28	-2.80	-3.51	-3.46	_-3.38_*	_-3.38_*	_-3.46_*
1→4	a{Q,5}Z			-3.75	_-3.66_	_-3.57_*	_-3.56_*	_-3.66_*
1→7	a{Q,5}Z			_-3.94_	_-3.79_	_-3.70_*	_-3.69_*	_-3.79_*

* The post-CCSD(T) slopes have been scaled by a CCSDT-2-CCSD(T) difference for larger basis sets.

Table S192: Best available correlation energy slopes (kJ/mol per double bond), including CCSDT(Q) values, of the polyene stack fixed dimers. Numbers in italic are estimates.

		Method						
				CCSD(T)		post-CCSD(T)		
Slopes	Basis Set	MP2	CCSD	`vvTight`	canonical	CCSDT-2	CCSDT	CCSDT(Q)
1→2	DZ	-2.98	-1.99	-2.45	-2.33	-2.36	-2.35	-2.41
1→3	DZ	-3.18	-2.08	-2.57	-2.46	-2.43	-2.41	_-2.54_
1→4	DZ	-3.31	-2.14	-2.64	-2.53	-2.54	_-2.49_	_-2.62_
1→7	TZ	-4.61	-2.88	-3.65	-3.55	_-3.56_*	_-3.49_*	_-3.67_*
1→2	{T,Q}Z	-4.45	-2.91	-3.64	-3.59	_-3.63_*	_-3.62_*	_-3.71_*
1→4	{T,Q}Z	-4.83	-3.08	-3.94	-3.84	_-3.84_*	_-3.78_*	_-3.95_*
1→7	{T,Q}Z			-4.10	_-4.01_	_-4.01_*	_-3.94_*	_-4.12_*
1→2	a{Q,5}Z	-4.31	-2.80	-3.55	-3.48	_-3.51_*	_-3.51_*	_-3.59_*
1→4	a{Q,5}Z			-3.77	_-3.67_	_-3.67_*	_-3.61_*	_-3.78_*
1→7	a{Q,5}Z			_-3.93_	_-3.83_	_-3.84_*	_-3.77_*	_-3.95_*

* The post-CCSD(T) slopes have been scaled by a CCSDT-2-CCSD(T) difference for larger basis sets.

Assessment of the Results when extrapolating to larger Molecules

Our calculations on the CCSDT(Q) slopes have been done with a mere cc-pVDZ basis set. However, as we need CCSDT(Q) slopes at the basis set limit, we need to turn towards more approxi-

mate methods and larger basis sets in Tables S193 and S197 (cp-corrected and not cp-corrected, respectively). Note that here, we just report the slope of benzene to naphtalene in order to stay on the same footing. Next as we are interested in the difference of these approximate methods to the CCSD(T) slopes, we look at these for different basis sets in comparison to CCSD(T) in Tables S194 and S198 (cp-corrected and not cp-corrected, respectively), noticing that for larger basis sets, the difference in slopes also become increasingly large. Finally, we can evaluate these slope differences to CCSD(T) in comparison to the largest CCSD(T) basis set combination, the extrapolated cc-pV(T,Q)Z values, close to the basis set limit, in Tables S195 and S199: For example, the cp-corrected slopes of MP2 differ from CCSD(T) 19% at the cc-pVDZ basis set from those at the extrapolated cc-pV(T,Q)Z values. The non-corrected slopes of MP2 differ from CCSD(T) 18% at the cc-pVDZ basis set from those at the extrapolated cc-pV(T,Q)Z values. In contrast, the CCSD slopes are markedly different to CCSD(T) at 40% and 28% for the cp-corrected and non-cp-corrected results, respectively. Finally, as we have CCSDT-2 numbers in Table S194 for a small triple-zeta basis set, we can compare to that one in Table S196. Here, we get a deviation of CCSDT-2 from the CCSD(T) slopes of 23%, compared 15% of MP2 and 28% of CCSD. These values, thus, lead us to a conclusion we will probably obtained a cp-corrected basis set incompleteness of about 33% for difference of the CCSD(T)-CCSDT-2 slope differences for CCSDT(Q). Taking an even more conservative estimate of the CCSD(T)-CCSD maximum slope difference, we arrive at the value of 40% utilized in the main text of the paper, though overall, considering all results, the value is probably closer to 20%-30% for the benzene-naphtalene series.

Please note, however, that the overall slopes will be merely changed if we believe that we underestimated the CCSDT(Q)-CCSD(T) differences by just 10%: Here, our best correlation interaction energy slopes will change by -0.1 kJ/mol per ring to -22.68 (cp-corrected) and -22.45 (cp-uncorrected) kJ/mol per ring when comparing to Table 2 of the main text.

Table S193: Slopes for the smallest 1→2 species (benzene to naphtalene dimer) for several methods and basis sets in kJ/mol per ring, cp-corrected.

	MP2	RPA	DFT-SAPT	CCSD	CCSD(T)	CCSDT-2	CCSDT	CCSDT(Q)
DZ(d,s)	-23.40			-13.25	-16.05		-15.23	
DZ	-24.19	-15.53	-15.91	-13.83	-16.67	-16.17	-15.92	-16.32
TZ(f,p)	-29.45			-16.63	-20.57	-19.93		
TZ	-29.59	-19.09	-19.25	-16.70	-20.65			
D,TZ	-31.87	-20.59	-20.66	-17.90	-22.33			
QZ	-31.23	-20.35	-20.70	-17.72	-22.09			
T,QZ	-32.43	-21.27	-21.76	-18.46	-23.14			

Table S194: Difference to CCSD(T) slopes for the smallest 1→2 species (benzene to naphtalene dimer) for several methods and basis sets in kJ/mol per ring, cp-corrected.

	MP2	RPA	DFT-SAPT	CCSD	CCSDT-2	CCSDT
DZ(d,s)	-7.35			2.80		0.82
DZ	-7.52	1.13	0.76	2.83	0.50	0.75
TZ(f,p)	-8.89			3.94	0.64	
TZ	-8.94	1.74	1.39	3.96		
D,TZ	-9.53	1.87	1.38	4.43		
QZ	-9.15	1.74	1.39	4.37		
T,QZ	-9.30	1.87	1.38	4.68		

Table S195: Difference to CCSD(T) slopes compared to the largest available (T,Q)Z basis set for the smallest 1→2 species (benzene to naphtalene dimer) for several methods in %.

	MP2	RPA	DFT-SAPT	CCSD
DZ(d,s)	-20.9			-40.2
DZ	-19.1	-39.4	-45.1	-39.5
TZ(f,p)	-4.4			-15.9
TZ	-3.8	-7.0	0.7	-15.4
D,TZ	2.6	0.0	0.0	-5.3
QZ	-1.6	-7.0	0.7	-6.5

Table S196: Difference to CCSD(T) slopes compared to the TZ(f,p) basis set for the smallest 1→2 species (benzene to naphtalene dimer) for several methods in %.

	MP2	CCSD	CCSDT-2
DZ(d,s)	-17.3	-28.9	
DZ	-15.4	-28.1	-22.7
TZ	0.6	0.5	
D,TZ	7.3	12.6	
QZ	2.9	11.1	
T,QZ	4.6	18.9	

Table S197: Slopes for the smallest 1→2 species (benzene to naphtalene dimer) for several methods and basis sets in kJ/mol per ring

	MP2	RPA	CCSD	CCSD(T)	CCSDT	CCSDT(Q)
DZ(d,s)	-26.51		-15.53	-18.68	-17.97	
DZ	-27.16	-19.00	-15.93	-19.32	-18.54	-18.94
TZ	-32.23	-23.12	-18.76	-23.12		
D,TZ	-34.36	-24.86	-19.94	-24.73		
QZ	-32.48	-22.47	-18.55	-23.10		
T,QZ	-32.66	-21.99	-18.40	-23.09		

Table S198: Difference to CCSD(T) slopes for the smallest 1→2 species (benzene to naphtalene dimer) for several methods and basis sets in kJ/mol per ring, cp-corrected.

	MP2	RPA	CCSD
DZ	-7.84	0.32	3.38
TZ	-9.10	0.00	4.37
D,TZ	-9.63	-0.13	4.78
QZ	-9.37	0.64	4.55
T,QZ	-9.57	1.10	4.69

Table S199: Difference to CCSD(T) slopes compared to the largest available (T,Q)Z basis set for the smallest 1→2 species (benzene to naphtalene dimer) for several methods in %.

	MP2	RPA	CCSD
DZ	-18.0	-71.2	-27.8
TZ	-4.8	-99.7	-6.8
D,TZ	0.7	-111.7	2.0
QZ	-2.0	-42.1	-2.9

Table S200: Differences in kJ/mol of correlation part of the interaction energies between post-CCSD(T) and CCSD(T) results, cp-corrected cc-pVDZ basis set for the Polyene stack relaxed dimers.

D-Bonds	CCSDT-2	CCSDT-3	CCSDT	CCSDT(Q)
1	0.028	0.028	0.017	-0.007
2	0.145	0.137	0.130	-0.025
3	0.288	0.263	0.300	
4	0.453	0.404		

Table S201: Differences in kJ/mol of correlation part of the interaction energies between post-CCSD(T) and CCSD(T)$_\lambda$ results, cp-corrected cc-pVDZ basis set for the Polyene stack relaxed dimers.

D-Bonds	CCSDT-2	CCSDT-3	CCSDT	CCSDT(Q)
1	0.012	0.012	0.001	-0.023
2	0.063	0.055	0.048	-0.107
3	0.130	0.105	0.142	
4	0.210	0.161		

Table S202: Differences in kJ/mol of correlation part of the interaction energies between post-CCSD(T) and CCSD(T) results, cp-uncorrected cc-pVDZ basis set for the Polyene stack relaxed dimers.

D-Bonds	CCSDT-2	CCSDT-3	CCSDT	CCSDT(Q)
1	0.029	0.028	0.020	-0.002
2	0.157	0.147	0.151	-0.005
3	0.316	0.288	0.346	
4	0.501	0.446		

Table S203: Differences in kJ/mol of correlation part of the interaction energies between post-CCSD(T) and CCSD(T)$_\lambda$ results, cp-uncorrected cc-pVDZ basis set for the Polyene stack relaxed dimers.

D-Bonds	CCSDT-2	CCSDT-3	CCSDT	CCSDT(Q)
1	0.013	0.013	0.004	-0.018
2	0.072	0.062	0.066	-0.090
3	0.148	0.120	0.178	
4	0.238	0.183		

Table S204: Differences in kJ/mol of correlation part of the interaction energies between post-CCSD(T) and CCSD(T) results, cp-corrected cc-pVDZ basis set for the Polyene stack fixed dimers.

D-Bonds	CCSDT-2	CCSDT-3	CCSDT	CCSDT(Q)
1	0.028	0.028	0.017	-0.007
2	0.085	0.080	0.076	-0.006
3	0.154	0.140	0.162	
4	0.229	0.203		

Table S205: Differences in kJ/mol of correlation part of the interaction energies between post-CCSD(T) and CCSD(T)$_\lambda$ results, cp-corrected cc-pVDZ basis set for the Polyene stack fixed dimers.

D-Bonds	CCSDT-2	CCSDT-3	CCSDT	CCSDT(Q)
1	0.012	0.012	0.001	-0.023
2	0.041	0.036	0.033	-0.049
3	0.078	0.064	0.086	
4	0.119	0.092		

Table S206: Differences in kJ/mol of correlation part of the interaction energies between post-CCSD(T) and CCSD(T) results, cp-uncorrected cc-pVDZ basis set for the Polyene stack fixed dimers.

D-Bonds	CCSDT-2	CCSDT-3	CCSDT	CCSDT(Q)
1	0.029	0.028	0.020	-0.002
2	0.088	0.082	0.085	0.004
3	0.160	0.145	0.178	
4	0.239	0.211		

Table S207: Differences in kJ/mol of correlation part of the interaction energies between post-CCSD(T) and CCSD(T)$_\lambda$ results, cp-uncorrected cc-pVDZ basis set for the Polyene stack fixed dimers.

D-Bonds	CCSDT-2	CCSDT-3	CCSDT	CCSDT(Q)
1	0.013	0.013	0.004	-0.018
2	0.045	0.040	0.042	-0.038
3	0.084	0.069	0.103	
4	0.129	0.100		

Finally, we would like to estimate the interaction energies of the stacked ethylene and transbutadiene dimers, as well as those of the stacked benzene and naphtalene dimers. For the stacked ethylene and transbutadiene dimers, the estimate is rather easy to calculate, as all interaction energies can be fully computed at the CCSD(T)/aug-cc-pV(Q,5)Z level of theory.

In case of ethylene, the extrapolated correlation part of the interaction energy is -2.95 (cp-corrected) and -2.94 (not cp-corrected) kJ/mol, and for HF (using an l^{-5} extrapolation scheme) it is 2.57 and 2.60 kJ/mol- adding up to an interaction energy of 0.38 and 0.34 kJ/mol. Since the CCSD(T)-CCSDT(Q) difference is very small and less than 0.01 kJ/mol in both cases, the interaction energy of ethylene is 0.36 $\pm$ 0.02 kJ/mol.

For transbutadiene, a similar procedure can be applied, with a correlation part of the interaction energy of -10.93 and -10.89 kJ/mol (cp-corrected and uncorrected), a HF energy of 7.97 and 8.05 kJ/mol, and a resulting CCSD(T) interaction energy of -2.96 and -2.84 kJ/mol. Again, the CCSD(T) and CCSDT(Q) differences are rather small with -0.04 and -0.01 kJ/mol, arriving at a best estimate of -2.92 $\pm$ 0.1 kJ/mol.

For the benzene and naphtalene dimers, this is more difficult, as we were unable to compute e.g. the naphtalene dimer with canonical CCSD(T)/aug-cc-pV5Z. Because of this, we estimated the basis set limit CCSD(T) values from LNO-CCSD(T) using Tables S208 and ??. Together with a cp-corrected HF/aug-cc-pV(Q,5)Z energy of benzene (14.66 kJ/mol) and naphtalene (27.54 kJ/mol), we arrive at the CCSD(T) values and estimates from LNO-CCSD(T) of Table S210. To these values, we add the post-CCSD(T) contributions of 0.45 (cp-corrected) and 0.57 kJ/mol (cp-uncorrected), which are both scaled 1.4$\times$DZ CCSDT(Q)-CCSD(T) differences for benzene. In a similar fashion, for naphtalene, the post-CCSD(T) contributions are 0.94 and 1.11 kJ/mol respectively. Finally, we arrive at our best estimates of Table S211, which are 6.5 $\pm$ 0.2 kJ/mol for benzene and 15.5 $\pm$ 0.2 kJ/mol for naphtalene. If we would consider a scaling factor of 1.2$\times$DZ, these values would barely shift to 6.6 $\pm$ 0.2 kJ/mol for benzene and 15.6 $\pm$ 0.2 kJ/mol for naphtalene, showing the robustness of our results.

Table S208: Counter-poise corrected CCSD(T) and LNO-CCSD(T) correlation contributions to interaction energies of Benzene and Naphtalene at various basis sets.

	CCSD(T)		LNO-CCSD(T)					
	canonical		vvtight		vtight		tight	
	Benzene	Naphtalene	Benzene	Naphtalene	Benzene	Naphtalene	Benzene	Naphtalene
DZ	-15.28	-31.95	-15.63	-32.77	-15.77	-33.01	-17.22	-35.69
aDZ	-21.15	-43.46	-21.40	-43.91	-21.49	-44.12	-21.85	-45.43
TZ	-19.05	-39.70	-19.28	-40.52	-19.32	-40.59	-20.62	-44.05
aTZ	-21.48	-44.15	-21.70	-44.92	-21.76	-45.04	-22.28	-46.24
QZ	-20.55	-42.64	-20.79	-43.33	-20.93	-43.55	-22.08	-45.57
TQZ	-21.64	-44.78	-21.89	-45.38	-22.10	-45.71	-23.15	-46.68
aQZ	-21.62		-21.82		-21.91	-45.29	-22.29	-46.47
aTQZ	-21.73		-21.91		-22.02	-45.46	-22.29	-46.64
5Z	-21.31		-21.78		-21.85	-44.83	-22.64	-46.65
Q5Z	-22.12		-22.81		-22.81	-46.17	-23.23	-47.79
a5Z			-21.91		-22.08	-45.37	-22.69	-46.57
aQ5Z			-22.00		-22.27	-45.46	-23.10	-46.68

Table S209: CCSD(T) and LNO-CCSD(T) correlation contributions to interaction energies of Benzene and Naphtalene at various basis sets.

	CCSD(T)		LNO-CCSD(T)					
	canonical		vvtight		vtight		tight	
canonical	Benzene	Naphtalene	Benzene	Naphtalene	Benzene	Naphtalene	Benzene	Naphtalene
DZ	-16.66	-35.97	-17.09	-37.09	-17.23	-37.34	-17.93	-38.88
aDZ	-27.38	-58.18	-27.74	-58.62	-27.82	-58.81	-28.46	-60.34
TZ	-20.72	-43.84	-21.03	-44.68	-21.07	-44.83	-22.01	-46.77
aTZ	-24.76	-52.34	-24.98	-52.97	-25.08	-53.16	-25.57	-54.37
QZ	-21.30	-44.40	-21.58	-45.14	-21.70	-45.37	-22.66	-47.20
TQZ	-21.72	-44.81	-21.99	-45.47	-22.16	-45.77	-23.12	-47.50
aQZ	-22.66		-22.87		-23.00	-47.70	-23.55	-48.92
aTQZ	-21.13		-21.34		-21.49	-43.71	-22.07	-44.94
5Z	-21.69		-21.97		-22.06	-45.82	-22.88	-47.47
Q5Z	-22.09		-22.37		-22.44	-46.28	-23.12	-47.76
a5Z			-22.33		-22.47	-46.34	-22.96	-47.71
aQ5Z			-21.76		-21.91	-44.91	-22.34	-46.44

Table S210: Full CCSD(T) interaction energies of Benzene and Naphtalene at various basis set extrapolations. Estimates from LNO-CCSD(T) are in italic.

cp-corrected	Benzene	Naphtalene
TQZ cor + aQ5Z HF	-6.98	-17.24
aTQZ cor + aQ5Z HF	-7.07	*-16.52*
aQ5Z cor + aQ5Z HF	*-7.14*	*-16.60*
cp-uncorrected		
TQZ cor + aQ5Z HF	-7.06	-17.26
aTQZ cor + aQ5Z HF	-6.47	*-15.21*
aQ5Z cor + aQ5Z HF	*-6.89*	*-16.40*

Table S211: Best estimates for the CCSDT(Q) interaction energies of Benzene and Naphtalene.

cp-corrected	Benzene	Naphtalene
CCSD(T)	-7.14	-16.60
CCSDT(Q) difference	0.45	0.94
CCSD(T)+ CCSDT(Q) difference	-6.70	-15.66
cp-uncorrected	Benzene	Naphtalene
CCSD(T)	-6.89	-16.40
CCSDT(Q) difference	0.57	1.11
CCSD(T)+ CCSDT(Q) difference	-6.31	-15.30

Strong Correlation Diagnostic of the Acene Species

We performed a strong correlation diagnostic for the acene series based on total atomization energy (TAE) calculations obtained with the cc-pVTZ basis set, both with the exchange part of TPSS as well as TPSS@HF, as written in:
Jan M.L. Martin, Golokesh Santra, and Emmanouil Semidalas, "An Exchange-Based Diagnostic for Static Correlation", AIP Conference Proceedings 2611, 020014 (2022). [ICCMSE-2021 special issue. DOI: 10.1063/5.0119280; ArXiv preprint: http://arxiv.org/abs/2111.01879
We used the formulae from the paper:

$$\%TAE_X[\Delta X, TPSS] = 100\% \times \frac{(TAE[X, TPSS@HF] - TAE[X, HF])}{TAE[CCSD(T)]} \tag{1}$$

and

$$\%TAE_X[\Delta X, KS - HF(TPSS)] = 100\% \times \frac{(TAE[X, TPSS@TPSS] - TAE[X, TPSS@HF]}{TAE[CCSD(T)]} \tag{2}$$

In equation 1, the difference in the total atomization energies between the TPSS exchange using a HF exchange wavefunction ($TAE[X, TPSS@HF]$) and the Hartree-Fock exchange part ($TAE[X, HF]$) is considered, divided by the total atomization energy of CCSD(T). In equation 2, the Hartree-Fock exchange part was replaced by the TPSS exchange at the optimized TPSS wavefunction.
The results indicate a minimal static correlation around 2 % (for example, water has a larger strong correlation diagnostic), not changing when the acenes get bigger.

Table S212: Total Atomization energies (in kJ/mol) for the acene series using a cc-pVTZ basis set.

TZ	Cx_Cx	TAEs					
System	Rings	X_TPSS@TPSS	X_TPSS@HF	HF	HF	CCSD(T)	LNO-CCSD(T)
				ORCA	MRCC		Tight
c_c	1	2767.17	2639.08	4284.48	4361.12	5571.56	5572.96
c2_c2	2	4474.61	4276.47	6729.33	6857.07	8802.09	8804.18
c3_c3	3	6173.99	5906.41	9152.95	9331.05	12019.42	12019.59
c4_c4	4	7874.54	7532.07	11566.04	11795.06	15228.34	15228.26
c5_c5	5	9566.89	9156.30	13973.25	14253.15		18433.18
c6_c6	6	11265.16	10779.42	16376.74	16707.53		21636.70

Table S213: Strong Correlation Diagnostic (in %) derived from TAEs of the acene series calculated using a cc-pVTZ basis set.

	Cx_Cx				
System	Rings	(TPSS@HF-HF)	(TPSS@HF-HF)	(TPSS@TPSS-TPSS@HF)	(TPSS@TPSS-TPSS@HF)
		CCSD(T)	LNO-CCSD(T) Tight	CCSD(T)	LNO-CCSD(T) Tight
c_c	1	-29.53	-29.52	2.30	2.30
c2_c2	2	-27.87	-27.86	2.25	2.25
c3_c3	3	-27.01	-27.01	2.23	2.23
c4_c4	4	-26.49	-26.49	2.25	2.25
c5_c5	5		-26.13		2.23
c6_c6	6		-25.87		2.25
